# The History of African Gene Flow into Southern Europeans, Levantines, and Jews

**DOI:** 10.1371/journal.pgen.1001373

**Published:** 2011-04-21

**Authors:** Priya Moorjani, Nick Patterson, Joel N. Hirschhorn, Alon Keinan, Li Hao, Gil Atzmon, Edward Burns, Harry Ostrer, Alkes L. Price, David Reich

**Affiliations:** 1 Harvard Medical School, Department of Genetics, Boston, Massachusetts, United States of America; 2 Broad Institute, Cambridge, Massachusetts, United States of America; 3 Children's Hospital, Boston, Massachusetts, United States of America; 4 Department of Biological Statistics and Computational Biology, Cornell University, Ithaca, New York, United States of America; 5 Human Genetics Program, Department of Pediatrics, New York University School of Medicine, New York, New York, United States of America; 6 Department of Medicine, Albert Einstein College of Medicine, Bronx, New York, United States of America; 7 Harvard School of Public Health, Boston, Massachusetts, United States of America; University of Oxford, United Kingdom

## Abstract

Previous genetic studies have suggested a history of sub-Saharan African gene flow into some West Eurasian populations after the initial dispersal out of Africa that occurred at least 45,000 years ago. However, there has been no accurate characterization of the proportion of mixture, or of its date. We analyze genome-wide polymorphism data from about 40 West Eurasian groups to show that almost all Southern Europeans have inherited 1%–3% African ancestry with an average mixture date of around 55 generations ago, consistent with North African gene flow at the end of the Roman Empire and subsequent Arab migrations. Levantine groups harbor 4%–15% African ancestry with an average mixture date of about 32 generations ago, consistent with close political, economic, and cultural links with Egypt in the late middle ages. We also detect 3%–5% sub-Saharan African ancestry in all eight of the diverse Jewish populations that we analyzed. For the Jewish admixture, we obtain an average estimated date of about 72 generations. This may reflect descent of these groups from a common ancestral population that already had some African ancestry prior to the Jewish Diasporas.

## Author Summary

Southern Europeans and Middle Eastern populations are known to have inherited a small percentage of their genetic material from recent sub-Saharan African migrations, but there has been no estimate of the exact proportion of this gene flow, or of its date. Here, we apply genomic methods to show that the proportion of African ancestry in many Southern European groups is 1%–3%, in Middle Eastern groups is 4%–15%, and in Jewish groups is 3%–5%. To estimate the dates when the mixture occurred, we develop a novel method that estimates the size of chromosomal segments of distinct ancestry in individuals of mixed ancestry. We verify using computer simulations that the method produces useful estimates of population mixture dates up to 300 generations in the past. By applying the method to West Eurasians, we show that the dates in Southern Europeans are consistent with events during the Roman Empire and subsequent Arab migrations. The dates in the Jewish groups are older, consistent with events in classical or biblical times that may have occurred in the shared history of Jewish populations.

## Introduction

The history of human migrations from Africa into West Eurasia is only partially understood. Archaeological and genetic evidence indicate that anatomically modern humans arrived in Europe from an African source at least 45,000 years ago, following the initial dispersal out of Africa [1], [2]. However, it is known that Southern Europeans and Levantines (people from modern day Palestine, Israel, Syria and Jordan) have also inherited genetic material of African origin due to subsequent migrations. One line of evidence comes from Y-chromosome [3] and mitochondrial DNA analyses [4]–[6]. These have identified haplogroups that are characteristic of sub-Saharan Africans in Southern Europeans and Levantines but not in Northern Europeans [7]. Auton et al. [8] presented nuclear genome-based evidence for sharing of sub-Saharan African ancestry in some West Eurasians, by identifying a North-South gradient of haplotype sharing between Europeans and sub-Saharan Africans, with the highest proportion of haplotype sharing observed in south/southwestern Europe. However, none of these studies used genome-wide data to estimate the proportion of African ancestry in West Eurasians, or the date(s) of mixture. Throughout this report, we use “African mixture” to refer to gene flow into West Eurasians since the divergence of the latter from East Asians; thus, we are not referring to the much older dispersal out of Africa ∼45,000 years ago but instead to migrations that have occurred since that time.

## Results

We assembled data on 6,529 individuals drawn from 107 populations genotyped at hundreds of thousands of single nucleotide polymorphisms (SNPs) (Table S1). This included 3,845 individuals from 37 European populations in the Population Reference Sample (POPRES) [9], [10], 940 individuals from 51 populations in the Human Genome Diversity Cell Line Panel (HGDP-CEPH) [11], [12], 1,115 individuals from 11 populations in the third phase of the International Haplotype Map Project (HapMap3) [13], 392 individuals who self reported as having Ashkenazi Jewish ancestry from the InTraGen Population Genetics Database (IBD) [14] and 237 individuals from 7 populations in the Jewish HapMap Project [15]. For most analyses, we used HapMap3 Utah European Americans (CEU) to represent Northern Europeans and HapMap3 Yoruba Nigerians (YRI) to represent sub-Saharan Africans, although we also verified the robustness of our inferences using alternative populations.

We curated these data using Principal Components Analysis (PCA) [16] (Table S2), with the most important steps being: (i) Removal of 140 individuals as outliers who did not cluster with the bulk of samples of the same group, (ii) Removal of all 8 Greek samples as they separated into sub-clusters in PCA so that it was not clear which of these clusters was most representative, (iii) Splitting the Bedouins into two genetically discontinuous groups, and (iv) Reclassifying the 5 Italian groups into three ancestry clusters (Sardinian, Northern-Italy, and Southern-Italy) (see details in Text S1, Figure S1). A comparison of results before and after this curation is presented in Table S3, where we show that this data curation does not affect our qualitative inferences.

**Figure 1:**
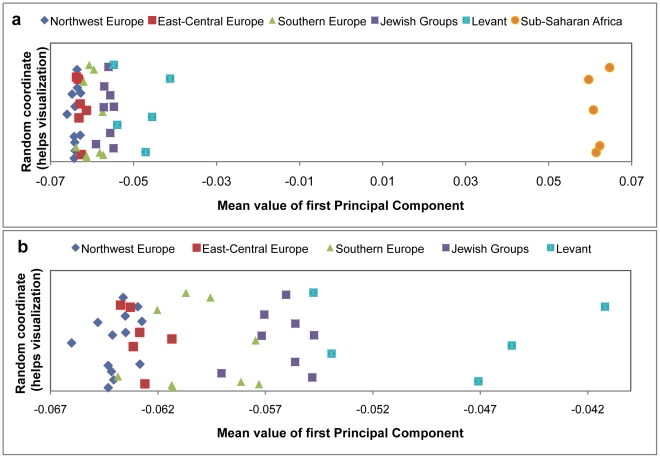
PCA Projection. PCA was performed using genome-wide SNP data from East Asians (HapMap3- CHB) and South Africans (HGDP-CEPH- San). All West Eurasians populations with samples sizes of *n* ≥ 5 were then projected onto these PCs. (a) The first panel presents data for all populations and (b) the second panel provides a higher resolution view of West Eurasians after removing sub-Saharan Africans. Each point on this graph indicates the mean value of the first PC for a projected population. West Eurasians populations are colored by 5 regional groupings—“Northwest Europe”, “East-Central Europe”, “Southern Europe”, “Levant”, “Jewish Groups” (the assignments of populations to groups is shown in Table 1). The grouping “Sub-Saharan Africa” refers to six populations from the HGDP-CEPH panel: Kenyan Bantu, South African Bantu, Mandenka, Mbuti Pygmy, Biaka Pygmy and Yoruba.

To study the signal of African gene flow into West Eurasian populations, we began by computing principal components (PCs) using San Bushmen (HGDP-CEPH- San) and East Eurasians (HapMap3 Han Chinese- CHB), and plotted the mean values of the samples from each West Eurasian population onto the first PC, a procedure called “PCA projection” [17], [18]. The choice of San and CHB, which are both diverged from the West Eurasian ancestral populations [19], [20], ensures that the patterns in PCA are not affected by genetic drift in West Eurasians that has occurred since their common divergence from East Eurasians and South Africans. We observe that many Levantine, Southern European and Jewish populations are shifted towards San compared to Northern Europeans, consistent with African mixture, and motivating formal testing for the presence of African ancestry (Figure 1, Figure S2).

To formally test for the presence of African mixture, we first performed the *4 Population Test* (Figure S3). This test is based on the insight that if populations *A* and *B* form sister groups relative to *C* and *D*, the allele frequency differences (p_A_-p_B_) and (p_C_-p_D_) should be uncorrelated as they represent independent periods of random genetic drift [21]. Applying the *4 Population Test* to the proposed relationship (YRI,(Papuan,(CEU,*X*))) where *X* is a range of West Eurasian populations, we find significant violations for all Southern European, Jewish and Levantine populations but not for Northern Europeans (Table 1). The results remain unchanged even when we use alternate topologies replacing YRI with other African populations (Text S2, Table S4). We further verified these inferences with the *3 Population Test* [21], which capitalizes on the insight that for any 3 populations (*X; A, B*), the product of the allele frequency differences (p_X_-p_A_) and (p_X_-p_B_) is expected to be negative only if population *X* descends from a mixture of populations related to populations *A* and *B* [21] (Figure S3). We verified that this method is robust to SNP ascertainment bias by carrying out simulations showing that the *3 Population Test* detects real admixture even if all SNPs used in the analysis are discovered in population *A*, population *B*, or in both populations *A* and *B* (Text S3; Table S5; Figure S4). Application of the test to each West Eurasian population (using *A* = YRI and *B* = CEU) finds little or no evidence of mixture in North Europeans but highly significant evidence in many Southern European, Levantine and Jewish groups (Table 1).

**Table 1:** Formal tests for population mixture.

Population (*X*)	Samples	Region	Dataset	Z-score for *4 Pop. Test* ((P_x_-P_CEU_),(P_Papuan_-P_YRI_))	Z-score for *3 Pop. Test* ((P_x_-P_CEU_),(P_x_-P_YRI_))
African Americans	49	n/a	HapMap3	**−85.1**	**−108.9**
Palestine	43	L	HGDP-CEPH	**−27.9**	**−24.7**
Turkey	6	L	POPRES	**−1**	**−3.4**
Bedouin-g1	15	L	HGDP-CEPH	**−36**	**−40.7**
Bedouin-g2	30	L	HGDP-CEPH	**−25.8**	>0
Druze	41	L	HGDP-CEPH	**−14.6**	>0
Spain	137	SE	POPRES	**−12.3**	**−21.1**
Portugal	134	SE	POPRES	**−14.9**	**−29**
Romania	14	SE	POPRES	−0.5	**−5.1**
Croatia	6	SE	POPRES	0.7	>0
Bosnia-Herzegovina	9	SE	POPRES	−0.6	−1.5
Sardinia	27	SE	HGDP-CEPH	**−9.3**	>0
Southern-Italy	121	SE	POPRES	**−10.7**	**−14.2**
Northern-Italy	90	SE	POPRES	**−5.7**	**−5.7**
Austria	14	ECE	POPRES	−0.2	−2.4
Poland	22	ECE	POPRES	1.3	>0
Hungary	19	ECE	POPRES	0.4	**−5.6**
Czech Republic	11	ECE	POPRES	0.5	>0
Adygei	17	ECE	HGDP-CEPH	2.9	>0
Russia	6	ECE	POPRES	0.6	−0.2
Russia	25	ECE	HGDP-CEPH	11.4	>0
Swiss-French	759	I	POPRES	**−3.2**	**−6.1**
France	92	I	POPRES	−1.9	**−3.7**
France	28	I	HGDP-CEPH	−1.9	−2.9
Basque	24	I	HGDP-CEPH	−1.2	>0
Belgium	43	I	POPRES	−0.9	−2.2
Orkney	15	I	POPRES	3.2	>0
United Kingdom	388	I	POPRES	1.5	>0
Ireland	62	I	POPRES	1.7	>0
Scotland	5	I	POPRES	3.3	>0
Netherlands	17	I	POPRES	1.0	>0
Swiss-German	84	I	POPRES	−1	−2.6
Germany	74	I	POPRES	−0.9	−2.8
Sweden	11	I	POPRES	1.6	0
Ashkenazi Jews	323	n/a	IBD	**−11.6**	>0
Ashkenazi Jews	34	n/a	Jewish HapMap	**−9.5**	−2.2
Syrian Jews	25	n/a	Jewish HapMap	**−10.1**	−2.3
Iranian Jews	24	n/a	Jewish HapMap	**−5.9**	>0
Iraqi Jews	36	n/a	Jewish HapMap	**−8.5**	>0
Sephardic Greek Jews	39	n/a	Jewish HapMap	**−13.7**	**−15.2**
Sephardic Turkey Jews	27	n/a	Jewish HapMap	**−13.6**	**−17.1**
Italian Jews	27	n/a	Jewish HapMap	**−11.4**	>0

Notes: We analyzed data from all West Eurasian populations with ≥5 samples. Regions are abbreviated: I – Northwest Europe, ECE – East-Central Europe, SE – Southern Europe and L – Levant. We used a *Block Jackknife* (block size of 5cM) to correct for LD among SNPs and to estimate a *Z*-score that reports the number of approximately normally distributed standard deviations that the correlation coefficient differs from 0. For the *4 Population Test*, we interpret |Z|>3 as significant evidence for mixture (we test the tree ((P_x_-P_CEU_)(P_Papuan_-P_YRI_), and do not show the tests of the two alternative trees, although all |Z|-scores are >16). For the *3 Population Test,* we interpret Z<−3 as significant evidence for mixture; a positive score for the *3 Population Test* is possible even in the presence of population mixture, since genetic drift after mixture can mask the signal (for example, Bedouin-g2). Scores that are significant are highlighted in bold. For further study of sub-Saharan African mixture, we chose populations with a significantly negative score by the *4 Population Test* (bold).

**Figure 2:**
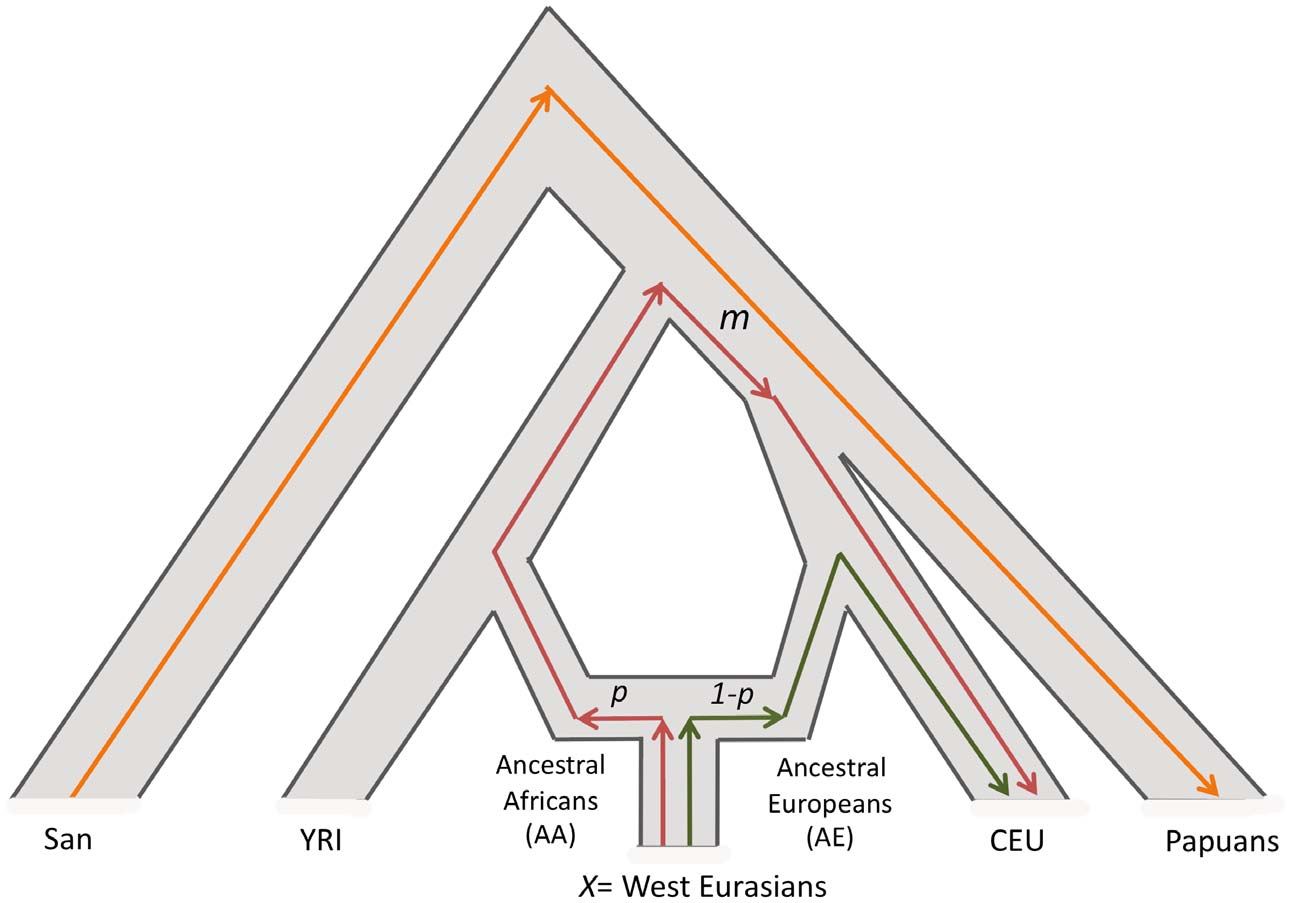
Estimation of African ancestry using *f_4_ Ancestry Estimation*. *f_4_ Ancestry Estimation* computes the quantity [(San-Papuan).(*X*–CEU)/[(San-Papuan).(YRI-CEU)]; where *X*  =  any West Eurasian population. The denominator is proportional to the genetic drift *m* that occurred in the ancestors of West or East Africans since their divergence from San but prior to their divergence from West Eurasians (intersection of red and orange lines). The numerator is proportion to p*(Ancestral Africans-YRI) + (1-*p*)*(Ancestral Europeans-CEU). Since the branches connecting (San, Papuan) and (CEU, *X*) do not overlap each other, the quantity (1-*p*)*(*X*-CEU)  =  0 and hence the numerator is expected to equal *pm*. Thus, the ratio of the numerator and denominator is expected to equal *p* (Ancestral African mixture proportion). This figure is adapted from reference [21], where we first developed *f_4_ Ancestry Estimation*, and where we reported computer simulations demonstrating its robustness.

To estimate the proportion of sub-Saharan African ancestry in the various West Eurasian populations that showed significant evidence of mixture, we used *f_4_ Ancestry Estimation* [21], a method which produces accurate estimates of ancestry proportions, even in the absence of data from the true ancestral populations. This method estimates mixture proportions by fitting a model of mixture between two ancestral populations, followed by (possibly large) population-specific genetic drift. Briefly, we calculate a statistic that is proportional to the correlation in the allele frequency difference between West Eurasians and sub-Saharan Africans, and divide it by the same statistic for a population of sub-Saharan African ancestry, like YRI (Figure 2). This method has been shown through simulation to be robust to ascertainment bias on the SNP arrays and deviations from the assumed model of mixture (e.g. date and number of mixture events) [21].

Application of *f_4_ Ancestry Estimation* suggests that the highest proportion of African ancestry in Europe is in Iberia (Portugal 3.2±0.3% and Spain 2.4±0.3%), consistent with inferences based on mitochondrial DNA [6] and Y chromosomes [7] and the observation by Auton et al. [8] that within Europe, the Southwestern Europeans have the highest haplotype-sharing with Africans. The proportion decreases to the north and we find no evidence for mixture in Russia, Sweden and Scotland (Table 2, Figure S5). We also detect about 3-5% sub-African ancestry in all the Jewish populations, a finding that is novel as far as we are aware, and certainly has not been unambiguously demonstrated or quantified. For Levantines, the proportions are often higher: 9.3%±0.4% in Palestinians and >10% in the Bedouins (standard errors were calculated using a Block Jackknife as described in Materials and Methods). Table 2 presents the ancestry estimates that we obtain for all West Eurasian populations with significant evidence of mixture by the *4 Population Test* (*Z-*score < -3). To test if our inferences are dependent on the sub-Saharan African population that was used as the reference group, we also repeated analyses with other sub-Saharan African populations replacing YRI. This analysis shows that our estimates of mixture proportions do not change significantly based on the ancestral population used (Text S2c, Table S6). We obtained similar estimates when we applied STRUCTURE 2.2 [22] to estimate the mixture proportions using ∼13,900 independent markers (that were not in linkage disequilibrium (LD) with each other) (Table 2, Figure S6).

**Table 2:** Estimates of mixture proportions and date of mixture.

Population (X)	Dataset	Region	Sam-ples	West African ancestry proportion ± standard error	West African ancestry proportion using STRUCTURE	Estimated date of admixture (generations ± standard error)	Bias from simulations (generations)*	Estimated date of admixture after bias correction
African Americans	HapMap3	n/a	49	79.4%±0.3%	77.2%	6±1	0	6±1
Palestinian	HGDP-CEPH	L	43	9.3%±0.4%	11.0%	34±2	1	33±2
Bedouin-g1	HGDP-CEPH	L	15	14.5%±0.4%	15.6%	34±3	2	32±3
Bedouin-g2	HGDP-CEPH	L	30	10.1%±0.4%	11.6%	33±2	2	31±2
Druze	HGDP-CEPH	L	41	4.4%±0.4%	5.6%	54±7	10	44±7
Spain	POPRES	SE	137	2.4%±0.3%	1.1%	55±3	0	55±3
Portugal	POPRES	SE	134	3.2%±0.3%	2.1%	45±5	0	45±5
Sardinian	HGDP-CEPH	SE	27	2.9%±0.5%	0.2%	96±28	25	71±28
Southern-Italy	POPRES	SE	121	2.7%±0.3%	1.7%	62±6	0	62±6
Northern-Italy	POPRES	SE	90	1.1%±0.3%	0.2%	154±27	−26	180±27
Swiss-French	POPRES	I	759	0.5%±0.2%	0.1%	71±6	n/a	n/a
Ashkenazi Jews	IBD	n/a	323	2.8%±0.3%	2.6%	91±11	n/a	n/a
Ashkenazi Jews	Jewish HapMap	n/a	34	3.2%±0.4%	2.6%	76±13	23	53±13
Syrian Jews	Jewish HapMap	n/a	25	3.9%±0.5%	4.1%	99±23	27	72±23
Iranian Jews	Jewish HapMap	n/a	24	2.6%±0.6%	4.6%	129±34	59	70±34
Iraqi Jews	Jewish HapMap	n/a	36	3.8%±0.5%	4.5%	153±22	38	115±22
Sephardic Greek Jews	Jewish HapMap	n/a	39	4.8%±0.4%	3.7%	82±8	20	62±8
Sephardic Turkey Jews	Jewish HapMap	n/a	27	4.5%±0.4%	4.3%	89±11	16	73±11
Italian Jews	Jewish HapMap	n/a	27	4.9%±0.5%	4.0%	88±19	15	73±19

Note: Estimates of the proportions and dates of mixture for all populations that give statistically significant evidence of mixture in Table 1 (*4 Population Test* Z<−3). Regions are abbreviated as: I – Northwest Europe, SE – Southern Europe and L – Levant. Mixture proportion estimates are based on *f_4_ Ancestry Estimation* using San, Yoruba, CEU and Papuan as the reference populations. The *ROLLOFF* estimated date of mixture uses CEU and YRI as the proposed ancestral populations (in the supplementary materials, we show that very similar inferences are obtained when the analysis is repeated with other ancestral populations, such as East Africans Luhya instead of Yoruba). Standard errors are computed using a Block Jackknife.

*Our simulations show that *ROLLOFF* produces a bias in the date estimates for small sample sizes, small mixture proportions, and old mixture dates. For each row of this table, we carried out a simulation to assess the expected bias for the inferred parameters (Table S12) and we computed the bias as (average - true date) in generations. Based on the simulation results, we have corrected the estimate in the last column as (estimated date - bias). We do not report a correction for the two rows marked “n/a” because our simulator cannot accommodate this large sample size.

**Figure 3:**
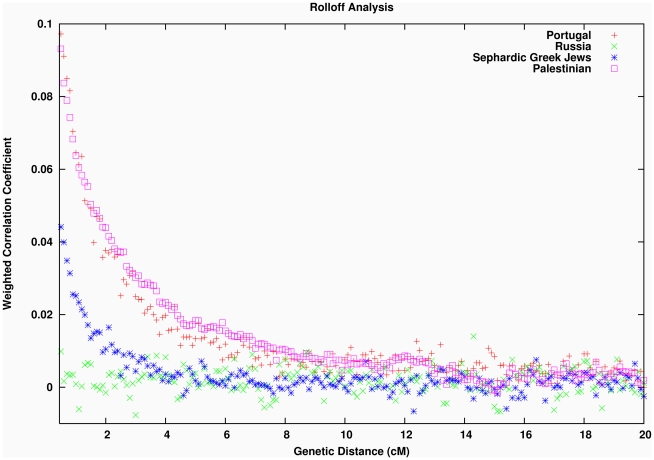
Testing for LD due to African admixture in West Eurasians. To generate these plots, we used the *ROLLOFF* software to calculate the LD between all pairs of markers in each population, weighted by their frequency difference between YRI and CEU to make the statistic sensitive to admixture LD. We plot the correlation as a function of genetic distance for Portuguese, Russians, Sephardic Greek Jews and Palestinians. We do not show inter-SNP intervals of <0.5cM since we have found that at this distance admixture LD begins to be confounded by background LD, and so inferences are not reliable (exponential curve fitting does not include inter-SNP intervals at this scale).

The finding of sub-Saharan African ancestry in West Eurasians predicts that there will be a signature of admixture LD in the populations that experienced this mixture. That is, there will be LD between all markers that are highly differentiated between the two ancestral populations and the allele will be strongly correlated to the local ancestry [23]. Hence, there will be chromosomal segments of African ancestry with lengths that reflect the number of recombination events that have occurred since mixture, and thus can be used to estimate an admixture date. Figure 3 shows that this expected pattern is observed empirically in the decay of LD in four example West Eurasian populations, where we enhance the effects of admixture LD by weighting the SNP comparisons by frequency difference between the ancestral Africans (YRI) and ancestral West Eurasians (CEU). In the Southern European, Jewish and Levantine populations, this procedure produces clear evidence of admixture LD (Figure 3). However, Northern Europeans (Russians in Figure 3) do not show any evidence of African gene flow, consistent with the *4 Population* and *3 Population Test* results and Figure 1. Similar results are seen for other West Eurasian and Jewish populations that show evidence of mixture in the *4 Population Test*.

To estimate a date for the mixture event, we developed a novel method *ROLLOFF* that computes the time since mixture using the rate of exponential decline of admixture LD in plots such as Figure 3. *ROLLOFF* computes the correlation between a (signed) statistic for LD between a pair of markers and a weight that reflects their allele frequency differentiation in the ancestral populations. By examining the correlation between pairs of markers as they become separated by increasing genetic distance and fitting an exponential distribution to this rolloff by least squares, we obtain an estimate of the date (see Materials and Methods and Text S4). *ROLLOFF* also computes an approximately normally distributed standard error by carrying out Weighted Jackknife analysis [24], where we drop one chromosome in each run and study the fluctuation of the statistic in order to assess the stability of the estimate.

**Figure 4:**
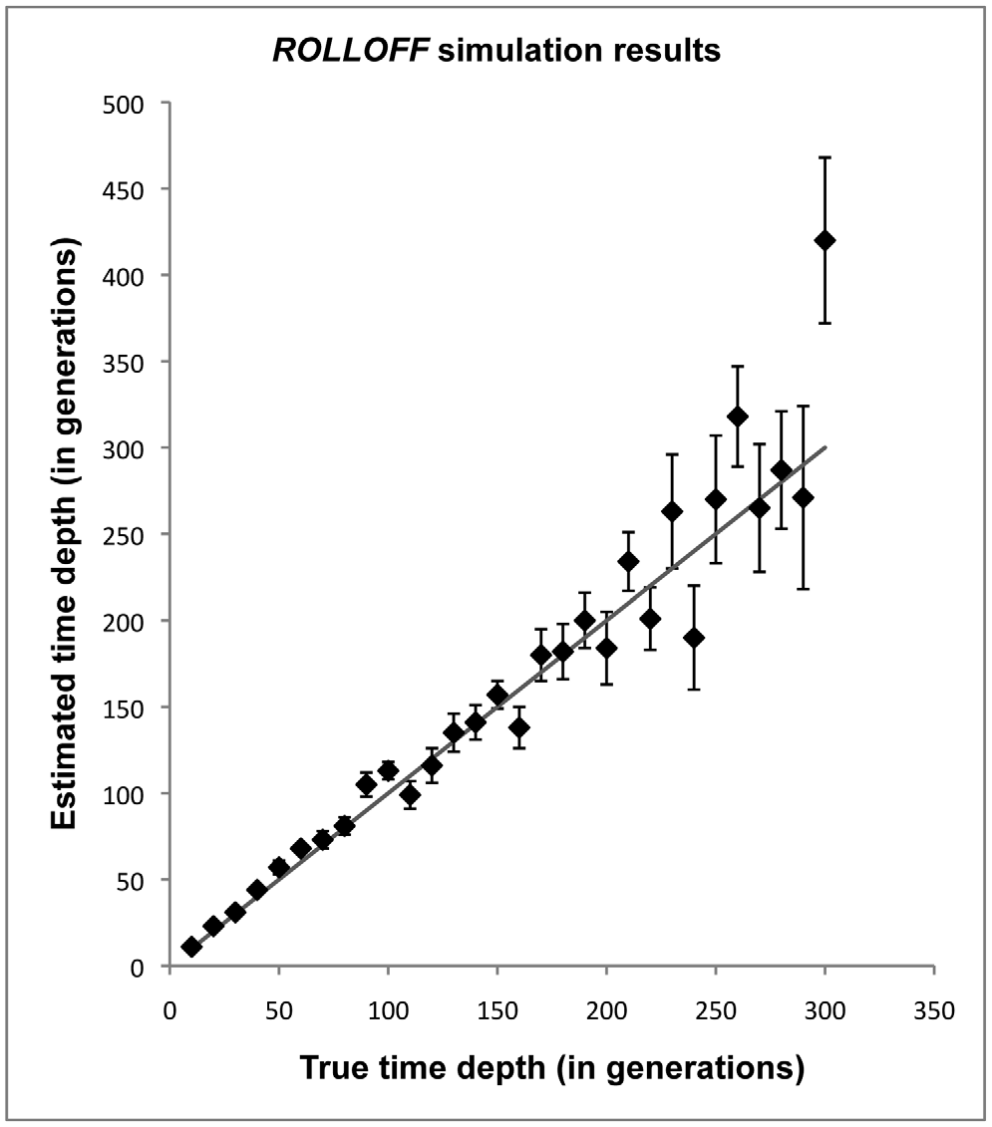
*ROLLOFF* simulation results. We constructed 10 individuals of mixed African and European ancestry (where individuals had 20% European ancestry) for various time depths ranging from 10–300 generations (with intervals of 10 generations). We performed *ROLLOFF* analysis using another independent dataset of European Americans and Nigerian Yoruba individuals as reference populations. We plot the true time depth (that was used for the simulations) against the estimated time depth computed by *ROLLOFF*. The expected time depth is shown as a dotted grey line. Standard errors were calculated using the *Weighted Block Jackknife* described in the Materials and Methods.

To verify the accuracy and sensitivity of *ROLLOFF*, we carried out extensive simulations by constructing the genomes of individuals of mixed ancestry by sampling haplotypes from North Europeans (CEU) and West Africans (YRI) (see Materials and Methods). We verified that *ROLLOFF* produces accurate estimates of the date of mixture, even in the case of old admixture (up to 300 generations – Figure 4) and is robust to substantially inaccurate ancestral populations as well as fine scale errors in the genetic map (Text S4; Figure S7; Figure S8; Table S7; Table S8). In addition, to test the robustness of our inferences, we applied all the methods to African Americans and obtained consistent results for the proportion of mixture (79.4±0.3%) and date of mixture (6±1), which is in agreement with previous reports [25], [26]. However, in the case of low mixture proportion and old admixture dates, we observed that there is a slight bias in the estimated date (Text S4d, Table S9). This effect is related to the weakness of the signal: it attenuates as the sample size or admixture proportion becomes larger (Text S4d, Table S10, Table S11).

An important concern was how *ROLLOFF* would perform when the true history of admixture involved multiple pulses of gene exchange, rather than the single pulse of gene exchange that we modeled. To explore this, we first simulated two distinct gene flow events, and then estimated the date using a single exponential distribution. The simulations show that *ROLLOFF*'s estimate of the date tends to correspond reasonably well to the more recent admixture event, with a slight upward bias towards the older date. Second, we performed simulations under a continuous gene flow model and found that the estimated dates are intermediate between the start and end of the gene flow, as expected (Figure S9; Figure S10; Table S12). To explore if we could obtain a better inference of the range of dates, we tried fitting sum of multiple exponential distributions, but this did not work reliably, which may be related to the well-known difficulty of fitting a sum of exponentials to data with even a small amount of noise [27] (Text S4). Pool and Nielsen recently showed that multi-marker haplotype data could be useful for distinguishing a single pulse of gene exchange from changing migration rates over time [28]. However, a complication with applying this approach to relatively old dates is that haplotype-based methods need to model background LD. In the case of old mixture events (dozens or hundreds of generations), inaccurate modeling of background LD can bias estimates [26], [29]. We are not aware of any published method that can produce accurate date estimates while modeling background LD correctly for mixture dates as old as those that have been explored by *ROLLOFF* in Figure 4.

We applied *ROLLOFF* to all the West Eurasian populations that gave significant signals of mixture by the *4 Population Test*, fitting a single exponential decay in each case. We estimate that the date of sub-Saharan African mixture in Portugal is 45±5 generations and in Spain is 55±3 generations. We estimate a more recent date of 34±3 for Bedouin-g1, 33±2 for Bedouin-g2, and 34±2 generations for Palestinians. We estimate older dates of ∼70–150 generations in the various Jewish populations, with wide and in most cases overlapping confidence intervals (Table 2; Figure S11). Averaging the mixture dates over all populations from each region (weighted by the inverse of the squared standard error), we obtain an average of 55 generations for Southern Europeans, 34 for Levantines and 89 for Jews.

As described above, in our simulations to explore the behavior of *ROLLOFF* we detect an upward bias in the date estimates that grew worse with older mixture dates, small mixture proportions, and small sample sizes (but does not appear to be affected by use of inaccurate ancestral populations). To assess the degree to which this bias might be affecting our date estimates, we performed simulations for each population in Table 2 separately, in which we set the number of samples, mixture proportion and time since mixture to match the parameters estimated from the real data. We repeated our simulations 100 times for each parameter setting and estimated the bias of our estimated date from the true (simulated) date. The bias is very small for the most of the Southern European and Levantine samples, which generally had large sample sizes, recent dates, and high mixture proportions. However, the bias is larger for the Jewish groups (Table 2, Table S13). Correcting for the bias inferred in our simulation of Table S12, we obtain corrected estimates of the average date of 55 generations for Southern Europeans, 32 for Levantines, and 72 for Jews. A caveat about these regional date estimates is that they reflect weighted averages across the populations in each region. However, the admixture events detected within each region may not reflect the same historical events; for example, it is plausible that the sub-Saharan African admixture in Spain and Italy have different historical origins.

## Discussion

The finding of African ancestry in Southern Europe dating to ∼55 generations ago, or ∼1,600 years ago assuming 29 years per generation [30], needs to be placed in historical context. The historical record documents multiple interactions of African and European populations over this period. One potential opportunity for African gene flow was during the period of Roman occupation of North Africa that lasted until the early 5^th^ century AD, and indeed tomb inscriptions and literary references suggest that trade relations continued even after that time [31], [32]. North Africa was also a supplier of goods and products such as wine and olive oil to Italy, Spain and Gaul from 200–600 AD, and Morocco was a major manufacturer of the processed fish sauce condiment, garum, which was imported by Romans [33]. In addition, there was slave trading across the western Sahara during Roman times [7], [34]. Another potential source of some of the African ancestry, especially in Spain and Portugal, is the invasion of Iberia by Moorish armies after 711 AD [35], [36]. If the Moors already had some African ancestry when they arrived in Southern Europe, and then admixed with Iberians, we would expect the admixture date to be older than the date of the invasion, as we observe.

The signal of African mixture that we detect in Levantines (Bedouins, Palestinians and Druze) – an average of 32 generations or ∼1000 years ago – is more recent than the signal in Europeans, which might be related to the migrations between North Africa and Middle East that have occurred over the last thousand years, and the proximity of Levantine groups geographically to Africa. Syria and Palestine were under Egyptian political control until the 16^th^ century AD when they were conquered by the Ottoman Empire. This is in concordance with our proposed dates. In addition, the Arab slave trade is responsible for the movement of large numbers of people from Africa across the Red Sea to Arabia from 650 to 1900 AD and probably even prior to the Islamic times [7], [37]. We caution that our sampling of the Middle East is sparse, and it will be of interest to study African ancestry in additional groups from this region.

A striking finding from our study is the consistent detection of 3–5% sub-Saharan African ancestry in the 8 diverse Jewish groups we studied, Ashkenazis (from northern Europe), Sephardis (from Italy, Turkey and Greece), and Mizrahis (from Syria, Iran and Iraq). This pattern has not been detected in previous analyses of mitochondrial DNA and Y chromosome data [7], and although it can be seen when re-examining published results of STRUCTURE-like analyses of autosomal data, it was not highlighted in those studies, or shown to unambiguously reflect sub-Saharan African admixture [15], [38]. We estimate that the average date of the mixture of 72 generations (∼2,000 years assuming 29 years per generation [30]) is older than that in Southern Europeans or other Levantines. The point estimates over all 8 populations are between 1,600–3,400 years ago, but with largely overlapping confidence intervals. It is intriguing that the Mizrahi Irani and Iraqi Jews—who are thought to descend at least in part from Jews who were exiled to Babylon about 2,600 years ago [39], [40]—share the signal of African admixture. (An important caveat is that there is significant heterogeneity in the dates of African mixture in various Jewish populations.) A parsimonious explanation for these observations is that they reflect a history in which many of the Jewish groups descend from a common ancestral population which was itself admixed with Africans, prior to the beginning of the Jewish diaspora that occurred in 8^th^ to 6^th^ century BC [41]. The dates that emerge from our *ROLLOFF* analysis in the non-Mizrahi Jews could also reflect events in the Greek and Roman periods, when there were large communities of Jews in North Africa, particularly Alexandria [34], [42]. We detect a similar African mixture proportion in the non-Jewish Druze (4.4±0.4%) although the date is more recent (54±7 generations; 44±7 after the bias correction). Algorithms such as PCA and STRUCTURE show that various Jewish populations cluster with Druze [15], which coupled with the similarity in mixture proportions, is consistent with descent from a common ancestral population. Importantly, the other Levantine populations (Bedouins and Palestinians) do not share this similarity in the African mixture pattern with Jews and Druze, making them distinct in their admixture history.

A caveat to these results is that we estimated dates assuming instantaneous mixture, but in fact we have not distinguished between the patterns expected for instantaneous admixture and continuous gene flow over a long period. In Text S4f, we report simulations showing that for continuous gene flow, the dates from *ROLLOFF* reflect the average of mixture dates over a range of times, and so the date should be interpreted only as an average number.

A potential issue that could in theory influence our findings is that the exact population contributing to African ancestry in West Eurasians is unknown. To gain insight into the African source populations, we carried out PCA analyses, which suggested that the African ancestry in West Eurasians is at least as closely related to East Africans (e.g. Hapmap3 Luhya (LWK)) as to West Africans (e.g. Nigerian Yoruba (YRI)) (the same analyses show that there is no evidence of relatedness to Chadic populations like Bulala) (Text S5 and Figure S12). We also used the *4 Population Test* to assess whether the tree ((LWK, YRI),(West Eurasian, CEU)) is consistent with the data, and found no evidence for a violation, which is consistent with a mixture of either West African or East African ancestors or both contributing to the African ancestry in West Eurasians (Table S14; Figure S13). Historically, a mixture of West and East African ancestry is plausible, since African gene flow into West Eurasia is documented from both West Africa during Roman times [34] and from East Africa during migrations from Egypt [7]. It is important to point out, however, that the difficulty of pinpointing the exact African source population is not expected to bias our inferences about the total proportion and date of mixture. The *f_4_ Ancestry Estimation* method is unbiased even when we use a poor surrogates for the true ancestral African population (as long as the phylogeny is correct), as we confirmed by repeating analyses replacing YRI with LWK, and obtaining similar results (Table S15). Our *ROLLOFF* admixture date estimates are also similar whether we use LWK or YRI to represent ancestral African population (Table S15), as predicted by the theory.

In summary, we have documented a contribution of sub-Saharan African genetic material to many West Eurasian populations in the last few thousand years. A priority for future work should be to identify the source populations for this admixture.

## Materials and Methods

### Datasets

We analyzed individuals of West Eurasian ancestry from several sources: The Population Reference Sample (POPRES) [9]–[10] (n = 3,845 samples from 37 populations genotyped on an Affymetrix 500K array), the Human Genome Diversity Cell Line Panel (HGDP*-*CEPH) [12] (n = 940 samples from 51 populations genotyped on an Illumina 650K array), The International Haplotype Map (HapMap) Phase 3 [13] (n = 1,115 samples from 11 populations genotyped on an Illumina 1M array), the InTraGen Population Genetics Database (IBD) [14] (n = 392 Ashkenazi Jews genotyped on an Illumina 300K array) and the Jewish HapMap Project [15] (n = 237 from 7 Jewish populations genotyped on an Affymetrix 6.0 array). We created a merged dataset containing 6,529 individuals -out of which 3,614 individuals of West Eurasian, African and Eastern Eurasian ancestry were used for the final analysis. Detailed information about the number of individuals and markers included in each analysis is provided in Table S1. We used NCBI Build 35 to determine physical position and the Oxford LD-based map genetic to determine genetic positions of all SNPs [43].

### Methods for characterizing mixture

#### Principal Component Analysis (PCA)

PCA was performed using *smartpca,* part of the EIGENSOFT 3.0 package [16]. For the PCA Projection analysis, the *poplistname* flag was used to compute Principal Components (PCs) on only a subset of populations from the dataset [17]–[18]. The merged dataset M with 36,175 SNPs was used for this analysis (Table S1).

#### 4 Population Test

For any 4 populations (*A*, *B*, *C*, *D*), there are three possible unrooted phylogenetic trees. If the tree ((*A*, *B*), (*C*, *D*)) is correct, then the genetic drift separating *A* and *B* should not be correlated to the drift separating *C* and *D*. However, if mixture occurred, then the correlation might be non-zero (Figure S3). We compute the correlation as in reference [21], and use a *Block Jackknife* [24], [44] that drops 5 centimorgan (cM) blocks of the genome in each run, to compute a standard error of the statistic. We convert the correlation into a *Z*-score and test for mixture by assessing whether the *Z*-score is more than 3 standard deviations different from 0. To test for sub-Saharan African mixture in West Eurasians, we tested the unrooted phylogenetic tree ((YRI,Papuan),(CEU,*X*)) where *X* is a range of West Eurasian populations. For this analysis, we intersected the HGDP-CEPH and HapMap3 data with all other datasets (POPRES, IBD, Jewish HapMap) to preserve the maximum number of SNPs. The merged datasets G, J, K and L with ∼606 K, ∼85 K, ∼284 K and ∼118 K SNPs respectively were used for these analyses (Table S1).

#### 3 Population Test

The *3 Population Test* can verify if population *X* is related to populations *A* and *B* through a simple tree or has arisen due to mixture. For a simple tree, the product of the frequencies differences between *A* and *X*, and *B* and *X*, is expected to be positive [21]. We compute a *Z*-score reporting the number of standard deviations that the statistic differs from 0, using the same Block Jackknife procedure as described above. A significantly negative value provides an unambiguous signal for mixture in *X* related to populations *A* and *B* [21] (also see Figure S3). For this analysis, we intersected HapMap3 dataset individually with all other datasets (HGDP-CEPH, POPRES, IBD, Jewish HapMap). The merged datasets F, G, H, I containing ∼347 K, ∼606 K, ∼284 K and ∼466 K SNPs respectively were used for the analysis (Table S1).

#### f_4_ Ancestry Estimation

We assume the population relationships shown in Figure 2 and denote the allele frequency of SNP *i* in each population as *p_San_^i^, p_Papuan_^i^ p_YRI_^i^ p_CEU_^i^* and *p_X_^i^* (*X*  =  any West Eurasian population). To estimate the proportion of sub-Saharan African ancestry in population *X*, we compute the ratio of two *4 Population Test* statistics:
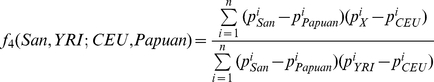
This quantity is summed over all markers and the standard errors are computed using the Block Jackknife [24], [44] (block size of 5 cM). The numerator is proportional to the amount of sub-Saharan African-related ancestry in population *X*, while the denominator is the same quantity for a population of entirely sub-Saharan African ancestry (YRI). Thus, the ratio estimates the mixture proportion [21] (Figure 2). The merged datasets G, J, K and L with ∼606 K, ∼85 K, ∼284 K and ∼118 K SNPs respectively were used for this analysis (Table S1).

#### STRUCTURE 2.2

To obtain an independent estimate of mixture proportions, we applied the model based clustering algorithm implemented in STRUCTURE 2.2 [22] to all populations that showed evidence of admixture using the *4 Population Test* (Table 1). As a control, we also added HapMap3 African Americans (ASW) and two Northern European populations, Russia and Sweden. To make the run tractable, we thinned the dataset to 13,877 SNPs by excluding all the SNPs that were in LD with other in a window of 0.1 cM. We ran STRUCTURE without any prior population assignment (unsupervised mode), with K = 2 and with 10,000 iterations for burn-in and 10,000 follow-on iterations. We used the INFERALPHA option under the admixture model.

### Estimating the date of admixture

#### Overview of ROLLOFF

To estimate dates of ancient admixture, we developed a method, *ROLLOFF*, which examines pairs of SNPs and assesses how admixture related LD decreases with genetic distance. The method is based on a novel LD statistic that weights SNPs according to their allele frequency differentiation between two populations that are genetically ‘close’ to the ancestral mixing populations.

Suppose that we have an admixed population and for simplicity assume that the population is homogeneous and that the mixture occurred over a short time span, ideally only a few generations. Call the two admixing populations *A*, *B*, and suppose that the admixture event occurred *n* generations before the present. If we consider two SNPs that are a distance *d* Morgans apart on a chromosome in an admixed individual, then with probability *e^-nd^* the alleles at these SNPs derived from a single admixing individual. If the mixing proportions are *p_A_* and *p_B_* respectively (*p_A_* + *p_B_*  =  1), then we see that:

1. With probability *e^-nd^p_A_*, both alleles belong to population *A*.
2. With probability *e^-nd^p_B_* both alleles belong to population *B*.
3. With probability (1-*e^-nd^*) the alleles belong to populations *A* or *B* independently.

We next suppose that we have a weight function at each SNP that is positive when the variant allele is more likely to be in population *A* than *B* and negative in the reverse situation. If *w(s)* is the weight of SNP *s*, then for any pair of SNPs *s_1_*, *s_2_*, we aim to compute an LD-based score *z(s_1_,s_2_)* that is asymptotically standard normal and positive if the two variant alleles are in admixture LD. As we explain below, the score *z(s_1_,s_2_)* and the product of the weight functions *w(s_1_)*·*w(s_2_)* are expected to be correlated, and to have a correlation coefficient exactly proportional to *e^-nd^*.

To convert the *z*-scores between all possible pairs of SNPs into an estimate of mixture age, we bin the *z*-scores based on the distance separations *d*, and compute the correlation coefficient between *z(s_1_,s_2_)* and *w(s_1_)*·*w(s_2_)* in each bin. Fitting an exponential distribution to the fall-off of the correlation coefficient with distance, we compute the admixture date from the fitted exponent. Our simulations show that the optimal bin size is at least 0.05 cM; smaller bins result in very short inter-SNP intervals so that analysis becomes confounded by background LD. In practice, we use a bin size of 0.1 cM.

#### Mathematical details of the ROLLOFF weight function

If we have data from two populations *A*' and *B*' that are genetically close to the admixing populations, then if *a*, *b* are the empirical allele frequencies at an allele for a SNP *s* in the two populations, we propose the weight function 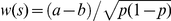 where *p  =  (a+b)/2*. A valuable feature of our *ROLLOFF* method is that we can also calculate useful weights even when no suitable surrogate parental populations are available (making it impossible to obtain direct estimates of the ancestral allele frequencies), by simply choosing a weight function that is proportional to the allele frequency difference, even if the absolute values cannot be computed directly.

#### Mathematical details of the ROLLOFF LD score z(s_1_, s_2_)

To compute an LD score 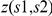 for two SNPs *s_1_* and *s_2_* we use the following procedure:

1. We compute the Pearson correlation coefficient 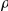 for the diploid genotypes at *s_1_* and *s_2_*. Samples with missing data at either marker are ignored. Let *N* be the number of samples with non-missing data. Setting 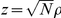 would probably be satisfactory but we slightly refine this. We insist that *N≥*4.
2. We ‘clip’ 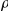 to fall within the interval [−0.9, 0.9].
3. We set 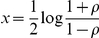, which is Fisher's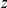-transformation.
4. We finally set 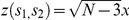

If the 2 markers (s_1_, s_2_) are unlinked, then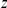 is roughly standard normal because of Fisher's *z-*transformation. Note that if the markers are unlinked, no matter how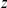 is defined, our weight function will be uncorrelated. This suggests that our method is robust to any reasonable definition of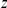.

#### Estimation of standard errors

We implemented a *Weighted Block Jackknife Test* [24], [44] where we drop one chromosome in each run and study the fluctuation of the statistic in the 22 runs. The statistic estimated in each run is weighted by the number of SNPs excluded in that run. By studying the variability of the estimated date, we compute the uncertainty in the inferred quantity via the theory of the jackknife [24]. These standard errors should be viewed with some caution as they reflect only 22 independent outcomes.

The reason we have chosen to carry out the jackknife on the scale of an entire chromosome is that we are concerned that LD due to admixture may extend sufficiently far for some populations that jackknifing by much smaller blocks (e.g. 10 Mb) may not completely remove the correlation among segments. We have therefore taken a conservative approach and set the block sizes to be equal to a chromosome. However, for a key West Eurasian population (Spain), we repeated the analysis with block sizes of 5 cM, 10 cM and 20 cM, as well as whole chromosomes and observed that the standard errors are similar (Table S16).

#### Simulation framework to test ROLLOFF

We simulated individuals of mixed European and African ancestry such that the genome of each individual is a mosaic of haplotypes from both the ancestral populations. The method we used is adapted from the simulation method that we previously described in reference [26]. Briefly, our simulations are based on two parameters: (a) the mixture proportion (θ) that gives the probability that a particular sampled haplotype comes from European or African gene pool, and (b) the time of mixture (λ) which can be viewed as the number of generations since mixture. We jointly phased data for 113 CEU individuals and 107 YRI individuals using fastPHASE [45] to create an ancestral haplotype pool of 226 haploid CEU and 214 haploid YRI genomes, which served as the source data for our simulations.

To simulate the genome of an admixed individual, we start at the beginning of each chromosome and sample European haplotypes with probability (θ) and African haplotypes with probability (1-θ). At each marker, we resample ancestry with probability of 1-e^-λg^ where *g* is the genetic distance in Morgans to determine if an event has occurred and then resample ancestry based on θ. Once the ancestry is chosen, a chromosomal segment of a randomly picked individual of that ancestry is then copied to the genome of the admixed individual and the process is continued until the end of chromosome is reached. This procedure is repeated to create the genomes of 20 admixed individuals, taking care that no chromosomal segment is reused (sampling without replacement). We combined pairs of haploid individuals to construct 10 diploid admixed individuals. This algorithm has one limitation that it requires more than 2*n* ancestral haplotypes for generating data for *n* diploid admixed individuals. Hence, in cases when we needed to simulate data for *n*≥50, we made a slight modification to the algorithm such that each admixed haploid genome is constructed from one haploid CEU and one haploid YRI genome, without reusing any chromosomal segments.

In order to test the performances for *ROLLOFF* at varying time depths, we performed 30 simulations. In each simulation, we constructed 10 diploid genomes of individuals of mixed European and African ancestry where we set λ = 10, 20…300 (interval  =  10 generations) and θ = 20%. We performed *ROLLOFF* analysis (for each of the simulations) using a non-overlapping dataset of 1,107 European American and 737 Nigerian Yoruba individuals as reference samples to compute the allele frequency in the ancestral populations. All analyses were restricted to 339,171 SNPs and the fine scale recombination map by Myers et al. [43] was used for mapping the genetic distance.

#### ROLLOFF analysis of West Eurasian populations

We ran *ROLLOFF* for various West Eurasian populations using the HapMap3 CEU and YRI as reference populations. The correlation between SNPs was plotted as a function of genetic distance. To estimate a date, we fitted an exponential distribution to the decay of the correlation coefficients. The merged datasets F, G, H, I with ∼347 K, ∼606 K, ∼284 K and ∼466 K SNPs respectively were used for this analysis (Table S1).

### Software

Source code and executables for the *ROLLOFF* software are available on request from NP.

## Supporting Information

Figure S1 PCA-based search for outliers and sub-structure. PCA was performed using YRI, CEU and X (where X  =  any West Eurasian population). A plot of the first and second PCs is shown all West Eurasian populations. Outliers (if any) are shown in pink boxes and labeled as X.Outlier. In three populations - Bedouins, Italians and Ashkenazi Jews - we observe significant population structure. The populations have been divided into multiple groups and PCA results both before outlier removal and reclassification are shown.
(1.68 MB DOC)

Figure S2 PCA Projection with Adygei and Kenyan Bantu. PCA was performed using genome-wide SNP data from Adygei and Kenyan Bantu. All West Eurasians populations with samples sizes greater than or equal to 5 were then projected onto these PCs. (a) The first panel presents data for all populations, (b) while the second provides a higher resolution view of West Eurasians after removing Sub-Saharan Africans. Each point on this graph indicates the mean value of the first PC for a projected population and West Eurasians populations are colored by 5 regional groupings-“Northwest Europe”, “East-Central Europe”, “Southern Europe”, “Levant”, “Jewish Groups”-with the assignments of populations to groups as shown in Table 1. The grouping “Sub-Saharan Africa” refers to six populations from the HGDP-CEPH panel: Kenyan Bantu, South African Bantu, Mandenka, Mbuti Pygmy, Biaka Pygmy and Yoruba. A qualitatively similar pattern is seen as in Figure 1.
(0.13 MB DOC)

Figure S3 Formal tests of admixture.
(1.73 MB DOC)

Figure S4 Demographic model to test the effect of ascertainment bias on 3 Pop. Test. We performed coalescent simulations using Hudson's ms [1] to generate data for two ancestral populations, Population A and Population B. For the simulation, we use a two-population demography where the effective population size of Pop A is N_0_  =  10,000 and the effective population size of Pop B varies from 0.25N_0_ to 0.85N_0_ such that the frequency differentiation F_ST_(A,B) = 0.15 and the divergence time varies from 45,000–100,000 years. Using data for Population A and B, we create Population C where individuals have mixed Population A and B ancestry. We set the mixture proportion to be 80%/20% and the time since mixture to be 10 generations.
(1.32 MB DOC)

Figure S5 Geographic gradient of African ancestry in Europeans. Sub-Saharan African ancestry proportions were estimated using f4 Ancestry Estimation. Populations in grey are estimated to have sub-Saharan African ancestry between 1–4%. The * in Switzerland indicates that the three populations available from this country have variable estimates: Swiss-Germans show no evidence of African mixture, Swiss-French 0.5±0.2% and Swiss-Italians 1.6±0.2%. The ‘+’ sign in Italy indicates that multiple samples were available but all show evidence of African mixture. No data are available from countries filled with diagonal lines. The map was downloaded from- http://www.ecozon.com/images/europe_map.jpg
(0.22 MB DOC)

Figure S6 Estimation of African ancestry using STRUCTURE. We applied STRUCTURE 2.2 to estimate the mixture proportions using ∼13,900 markers (selected to not be in LD with each other) and K = 2. Each individual is represented by a single line with the length of the different colors reflecting the individual ancestry proportions.
(0.18 MB DOC)

Figure S7 *ROLLOFF* simulation for a scenario similar to African Americans. We constructed genomes of 10 individuals with mixed European and African ancestry. We set the time since mixture (λ) at 6 generations and the European ancestry proportion (θ) was sampled from a beta distribution with mean 20% and standard deviation 10%. We performed *ROLLOFF* analysis with a non-overlapping dataset of European Americans and Yoruba Nigerians as reference populations. We plot the decay of weighted correlation coefficient as a function of genetic distance and estimate the date of admixture as 6±1 generations, by fitting an exponential distribution to the data.
(0.43 MB DOC)

Figure S8 *ROLLOFF* analysis in cases of no gene flow related to the tested ancestral populations. We performed *ROLLOFF* analysis for East Asian Uygurs, who have both West Eurasians and East Eurasian ancestry. We used YRI and Pygmies (Mbuti and Biaka Pygmies) as the reference populations in *ROLLOFF* and saw no evidence of mixture. To show that this is not because of an inability to detect mixture when YRI and Pygmy-related groups are the true ancestral populations, we simulated 10 individuals of mixed Pygmy and Yoruba ancestry, with Yoruba mixture proportion (θ)  =  80% and time since mixture (λ)  =  10 generations (10 individuals) and θ = 80% and λ = 100 generations (10 individuals). We plot the *ROLLOFF* weighted correlation coefficient against genetic distance and observe clear evidence of mixture in these samples, with fairly accurately estimated dates of 10 and 90 generations respectively.
(0.48 MB DOC)

Figure S9 *ROLLOFF* analysis for double admixture event. We simulated double admixture scenarios (two events of gene flow) in which a 50%/50% mixture of CEU and YRI mixture occurred at λ = 30 generations, followed by a 50%/50% mixture of that admixed population and YRI at λ = 10 generations. We performed *ROLLOFF* analysis using a non-overlapping dataset of Yoruba Nigerians and European Americans as reference populations. In the left panel, we fit a single exponential distribution to the output and estimate the date of the admixture event as 11 generations. In the right panel, we fit a sum of two exponentials and estimate the dates of admixture as 35 and 9 generations. In both cases, we accurately estimate the date of the most recent mixture event.
(0.23 MB DOC)

Figure S10 A demographic model for continuous admixture. To test the performance of *ROLLOFF* under continuous admixture scenarios, we simulate data for individuals with mixed ancestry using data for two ancestral populations CEU and YRI, where the gene flow occurs in an interval I  =  [a,b] where 0 < a ≤ b and the time is in generations. In each generation during I, we allow a proportion m (computed based on mixture proportion (θ)) of YRI lineages to migrate, yielding a total of 20% average African ancestry in the resulting admixed samples.
(0.06 MB DOC)

Figure S11 *ROLLOFF* analysis for West Eurasians. We performed *ROLLOFF* analysis for each West Eurasian population X that showed significant evidence of admixture in the 4 Population Test using YRI and CEU as reference populations. We plot the decay of admixture LD as a function of genetic distance and estimate the date of admixture by fitting an exponential distribution to the data. Standard errors were calculated using a Weighted Block Jackknife as described in the Materials and Methods.
(1.52 MB DOC)

Figure S12 Establishment of the axes of variation within Africa using PCA. To study the relationship of sub-Saharan African populations to each other and filter out populations with West Eurasian ancestry, we performed the following three analyses: (A) PCA of 15 sub-Saharan African groups using EIGENSOFT (B) PCA of 15 sub-Saharan African groups along with HapMap Chinese (CHB) and San Bushmen, and (C) PCA of 14 sub-Saharan African groups (excluding Kenyan Maasai).
(0.38 MB DOC)

Figure S13 Source of African ancestry in West Eurasians may include some East African ancestors. In order to identify the source of the African ancestry in Levantines, Southern Europeans and Jews, we performed PCA Projection with all three possible pairs of African populations (Bulala, Kenyan Luhya (LWK) and Yoruba (YRI)) along with HapMap Chinese (CHB), and then plotted the mean values of all the samples from each West Eurasian population, African Americans (ASW) and North African (Mozabites) onto the first PC and second PC. These admixed West Eurasian populations align along a gradient that points more at (a) YRI than Bulala, and (b) LWK than Bulala suggesting little evidence for Chadic ancestry in West Eurasians, (c) The PCA suggests more relatedness to LWK than to YRI, suggesting that there may be some East African related ancestry in these West Eurasian populations.
(0.23 MB DOC)

Table S1 Summary of datasets.
(0.06 MB DOC)

Table S2 Outlier samples removed based on PCA curation.
(0.04 MB DOC)

Table S3 Comparison of test statistics before and after PCA-based curation.
(0.09 MB DOC)

Table S4 4 Pop. Test using alternate ancestral populations compared to Table 1.
(0.10 MB DOC)

Table S5 Simulation to test the effect of ascertainment bias on 3 Pop. Test results.
(0.04 MB DOC)

Table S6 f4 Ancestry Estimation using different ancestral populations compared to Table 2.
(0.06 MB DOC)

Table S7 ROLLOFF Simulations: Effect of variations in bin sizes and genetic map.
(0.04 MB DOC)

Table S8 ROLLOFF Simulations: Effect of inaccurate ancestral populations.
(0.04 MB DOC)

Table S9 ROLLOFF Simulations: Effect of inaccurate ancestral populations in the case of low mixture proportions and old mixture dates.
(0.07 MB DOC)

Table S10 ROLLOFF Simulations: Effect of number of admixed samples.
(0.03 MB DOC)

Table S11 ROLLOFF Simulations: Effect of mixture proportions.
(0.03 MB DOC)

Table S12 ROLLOFF Simulations: Continuous admixture scenarios.
(0.04 MB DOC)

Table S13 ROLLOFF analysis of West Eurasians: bias in the estimated date for empirically estimated parameters.
(0.07 MB DOC)

Table S14 4 Population Test to distinguish between East & West African ancestry.
(0.05 MB DOC)

Table S15 Estimated mixture proportion and date using East Africans as reference.
(0.05 MB DOC)

Table S16 ROLLOFF Analysis for different jackknife block sizes: example Spain.
(0.03 MB DOC)

Text S1 PCA-based search for outliers and sub-structure.
(0.03 MB DOC)

Text S2 Robustness of inferences to the choice of ancestral populations.
(0.04 MB DOC)

Text S3 Effect of SNP ascertainment bias on results of 3 Population Test.
(0.03 MB DOC)

Text S4 Robustness of the ROLLOFF method for estimating mixture dates.
(0.09 MB DOC)

Text S5 Searching for the source of African ancestry in West Eurasians.
(0.05 MB DOC)
