# Supplementary material for: The History of African Gene Flow into Southern Europeans, Levantines, and Jews
Source: PLoS Genet. 2011 Apr 21;7(4):e1001373. doi: 10.1371/journal.pgen.1001373 (PMC3080861; doi:10.1371/journal.pgen.1001373)
Supplement: Figure S1 — PCA-based search for outliers and sub-structure. PCA was performed using YRI, CEU and X (where X = any West Eurasian population). A plot of the first and second PCs is shown all West Eurasian populations. Outliers (if any) are shown in pink boxes and labeled as X.Outlier. In three populations - Bedouins, Italians and Ashkenazi Jews - we observe significant population structure. The populations have been divided into multiple groups and PCA results both before outlier removal and reclassification are shown. (1.68 MB DOC) [file pgen.1001373.s001.doc]

**Figure S1.** **PCA-based search for outliers and substructure in West Eurasians**

**A. Northwest Europe**

**
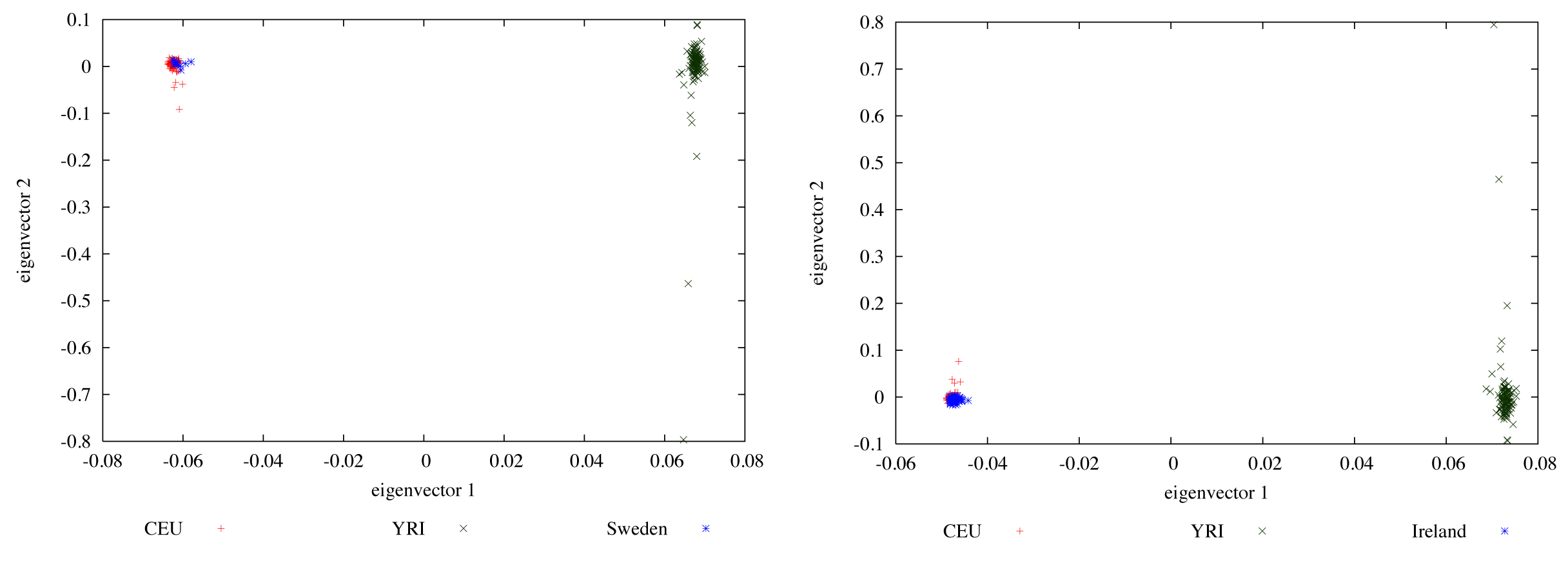
**

**
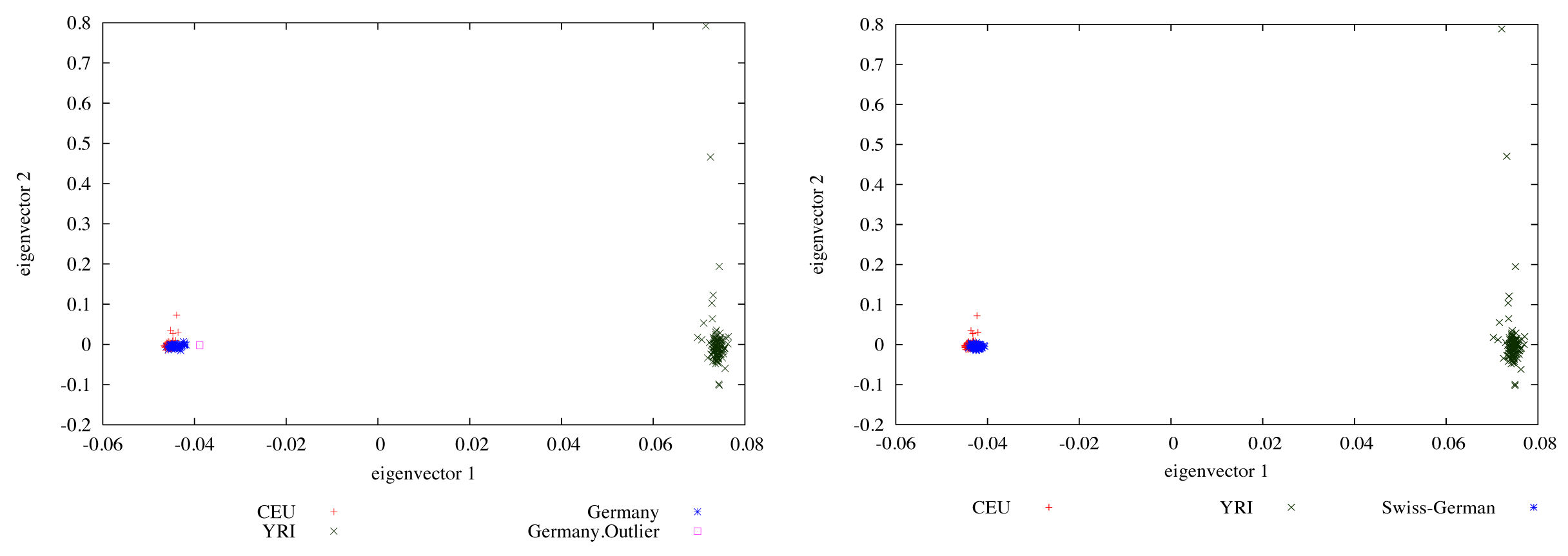
**

**
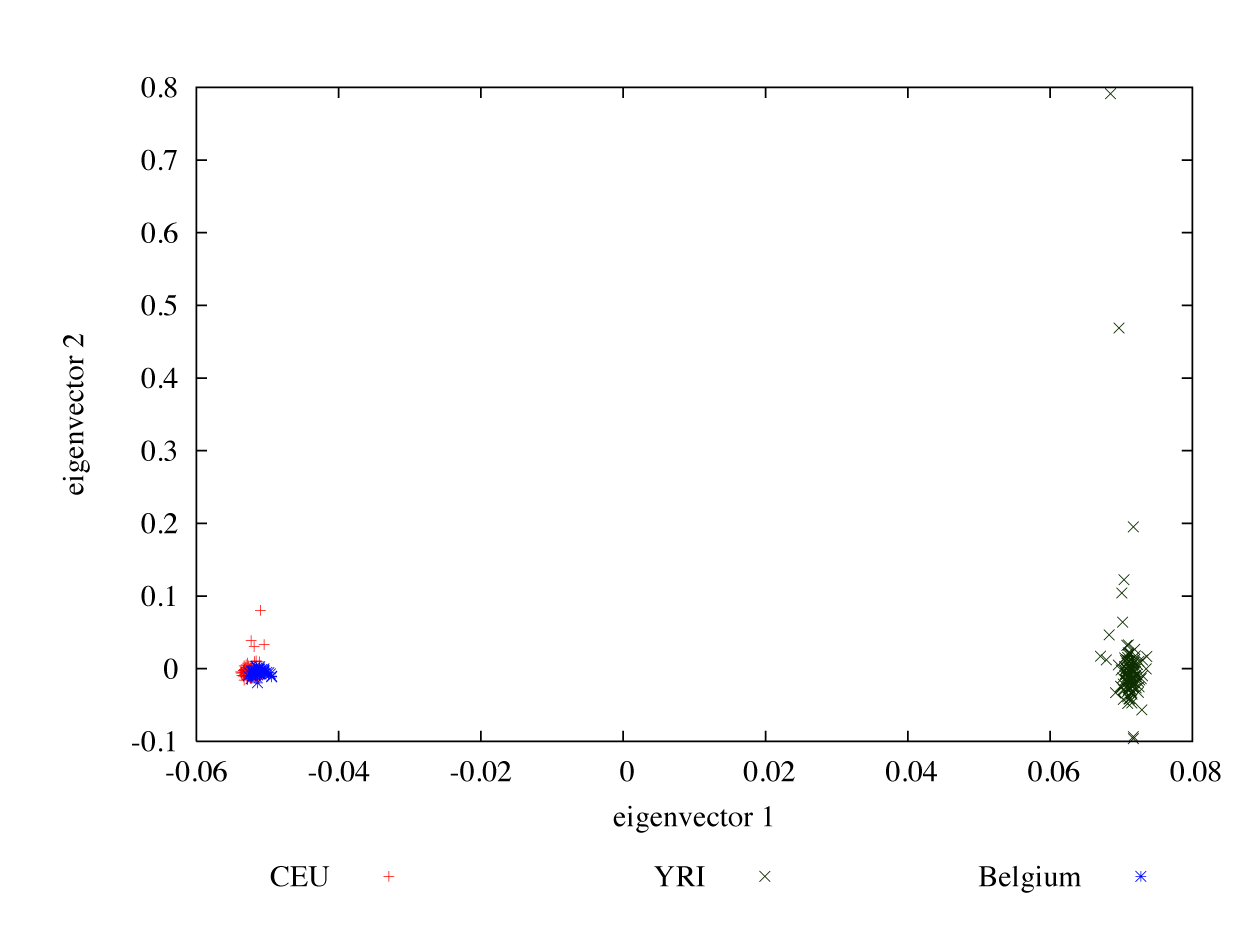

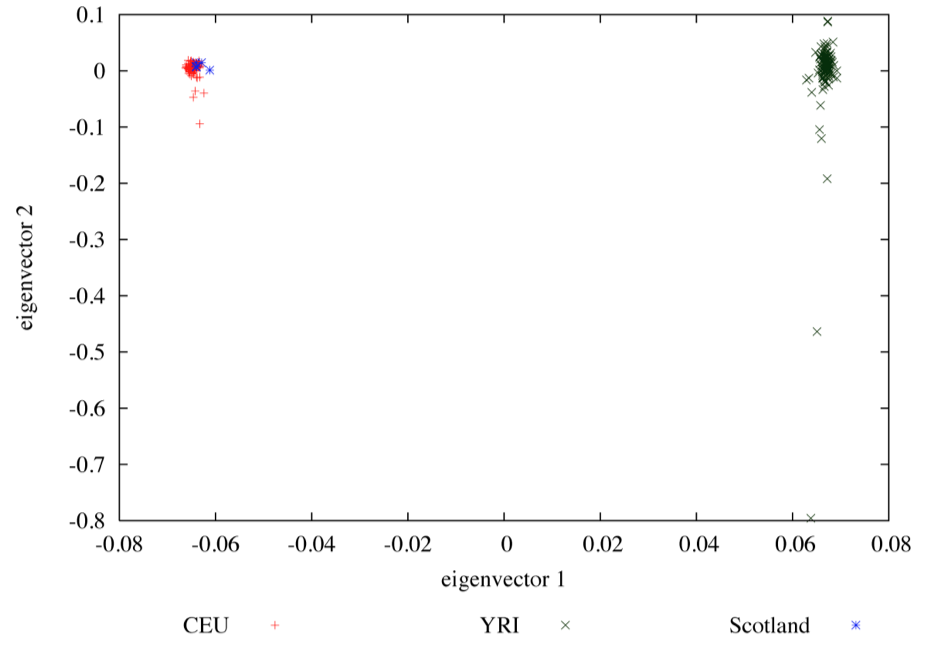
**

**
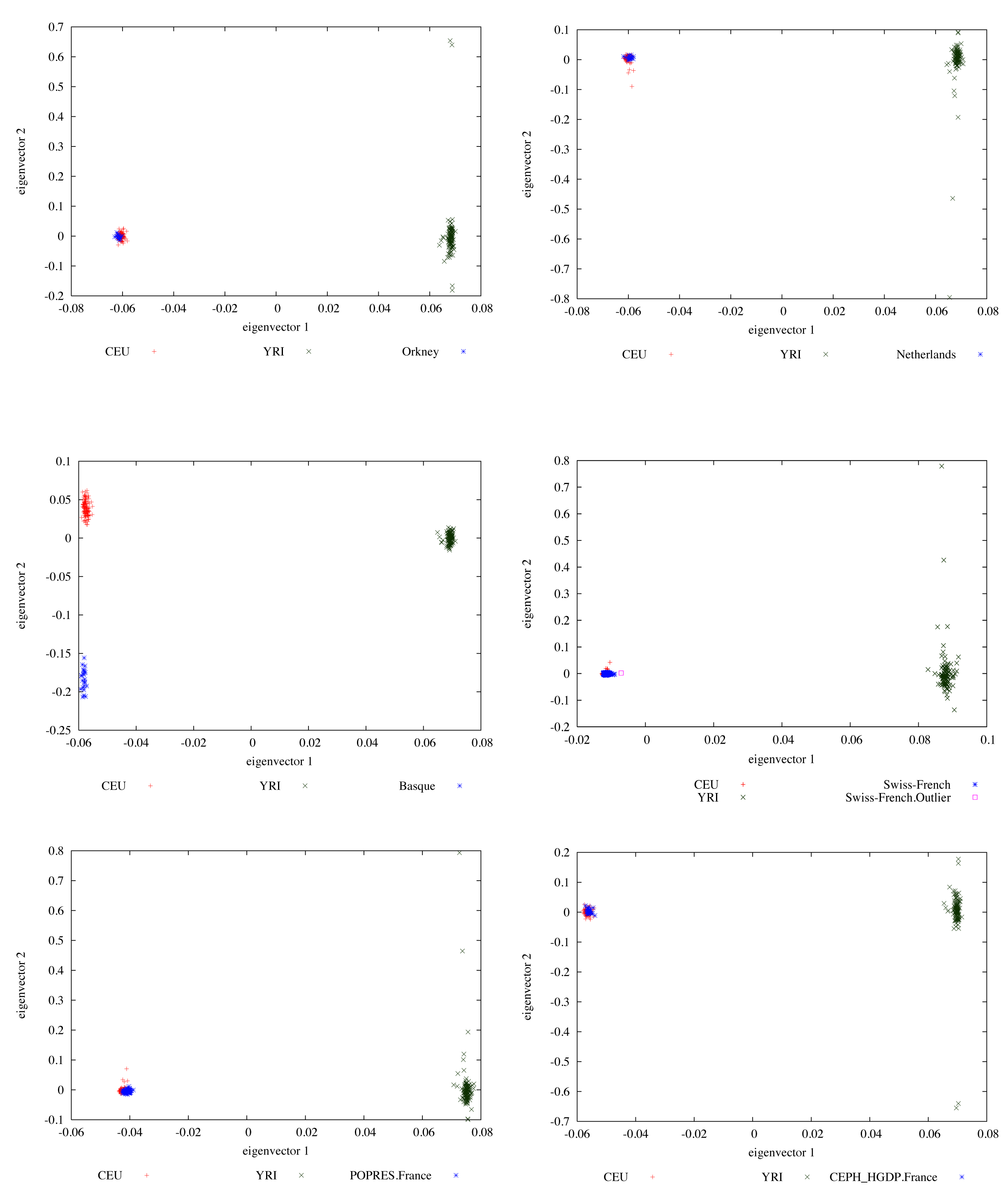
**

**B. East-Central Europe**

**
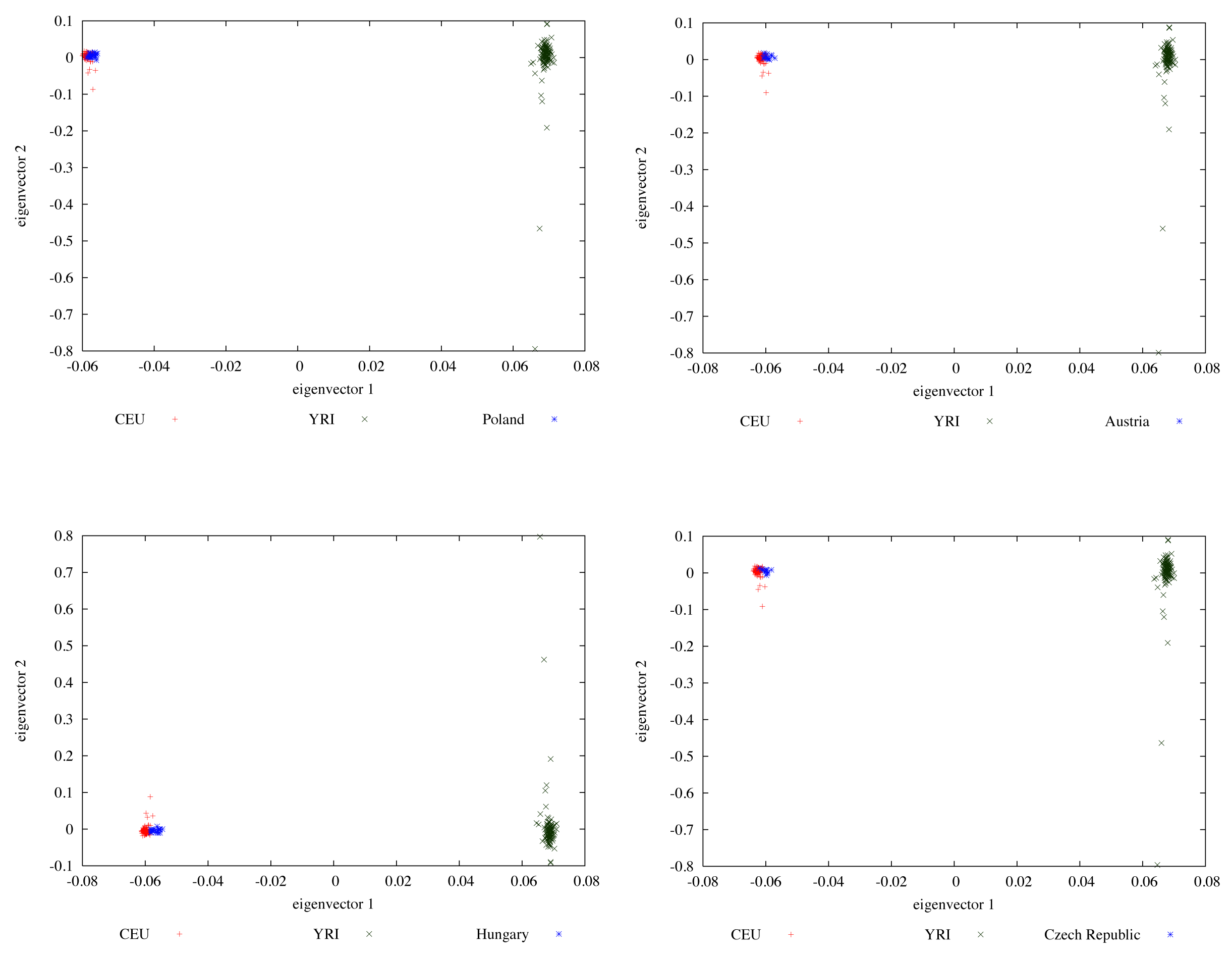
**

**
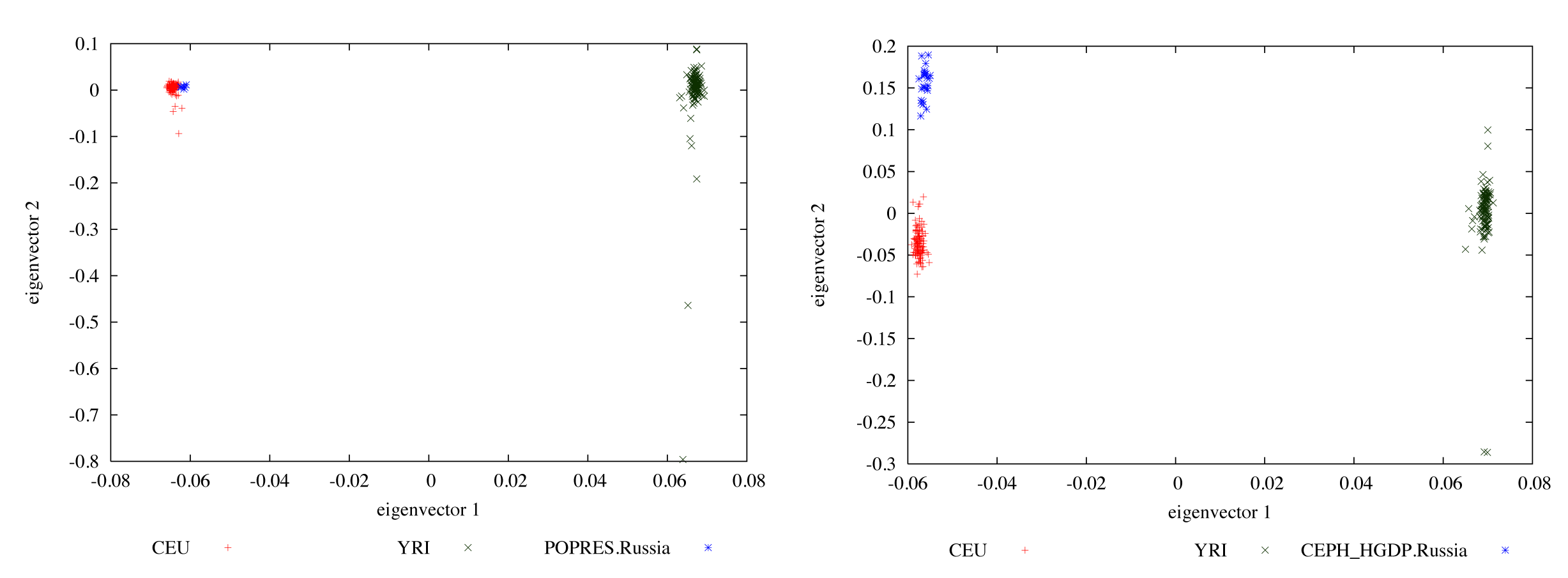
**

**C. Southern Europe**

**
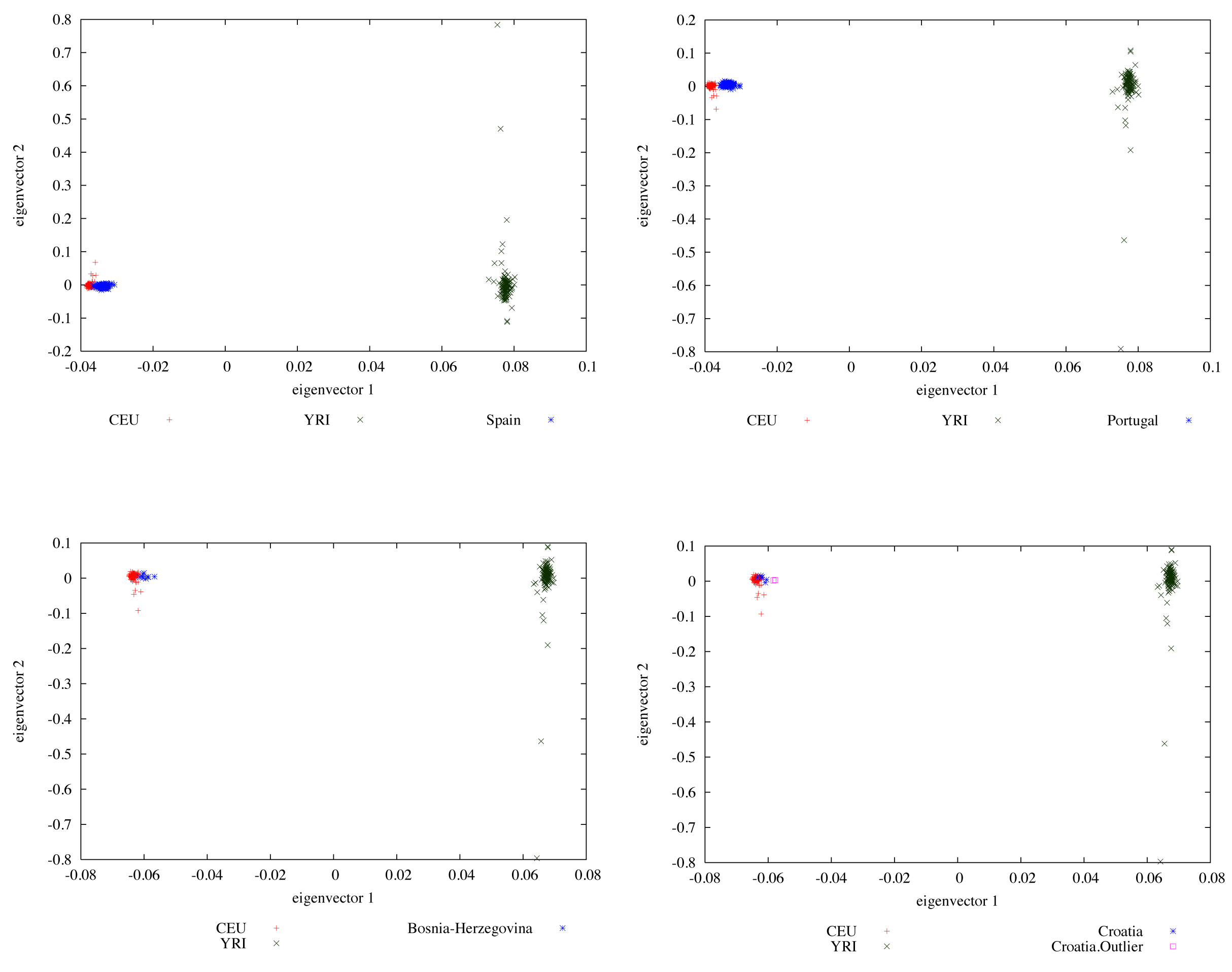
**

**
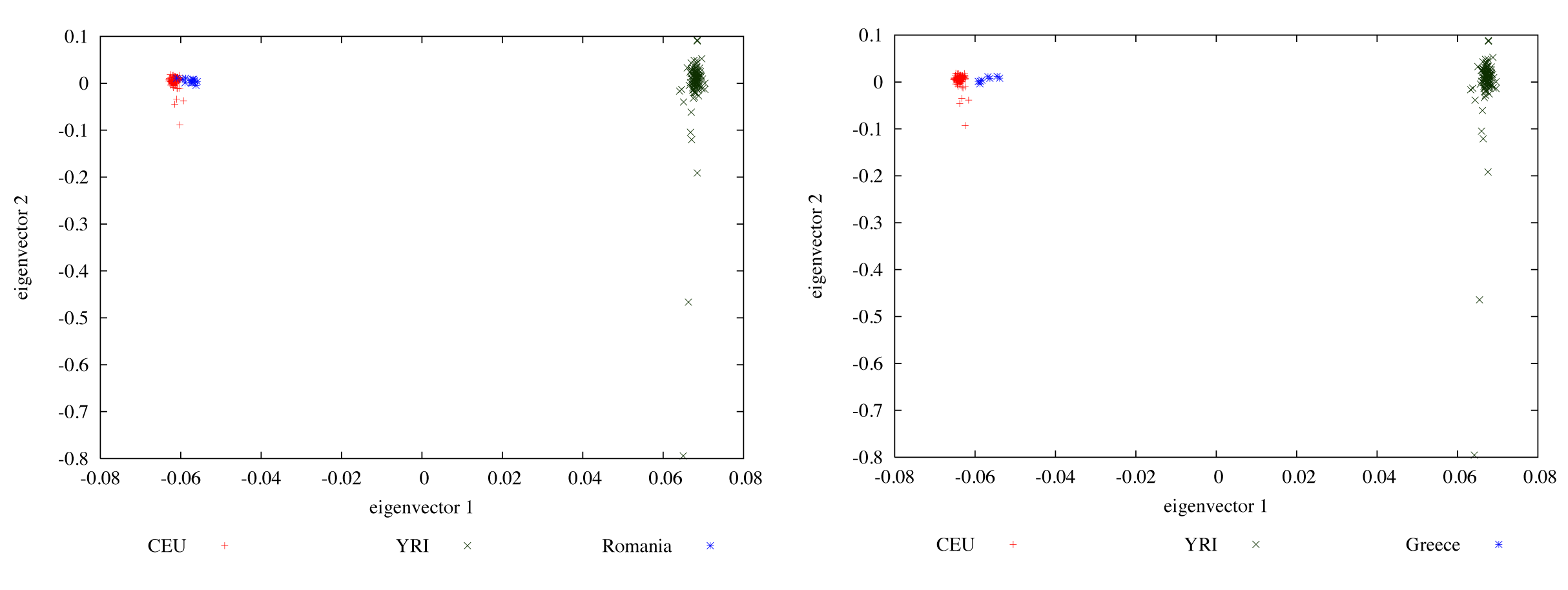
**

**
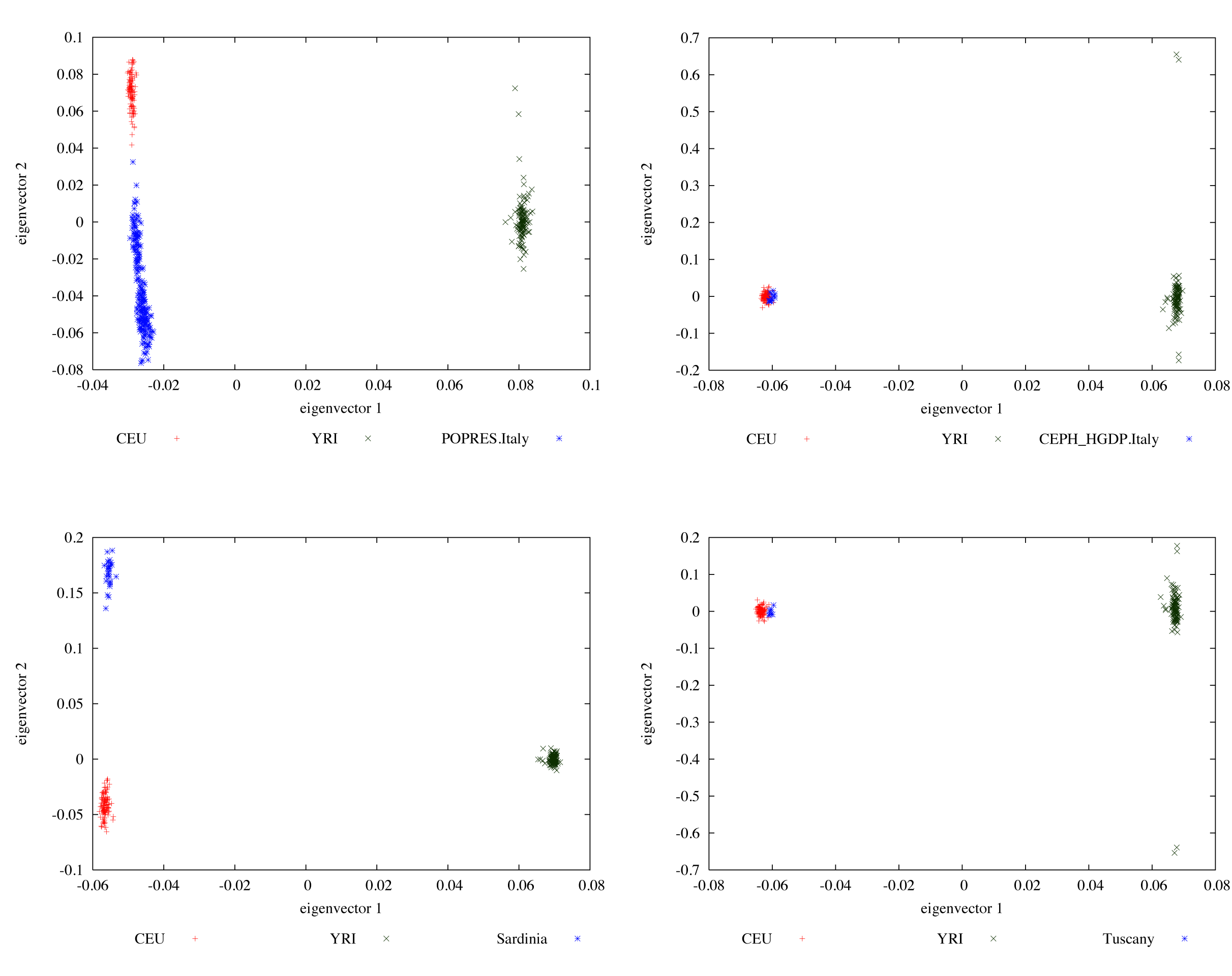
**

**
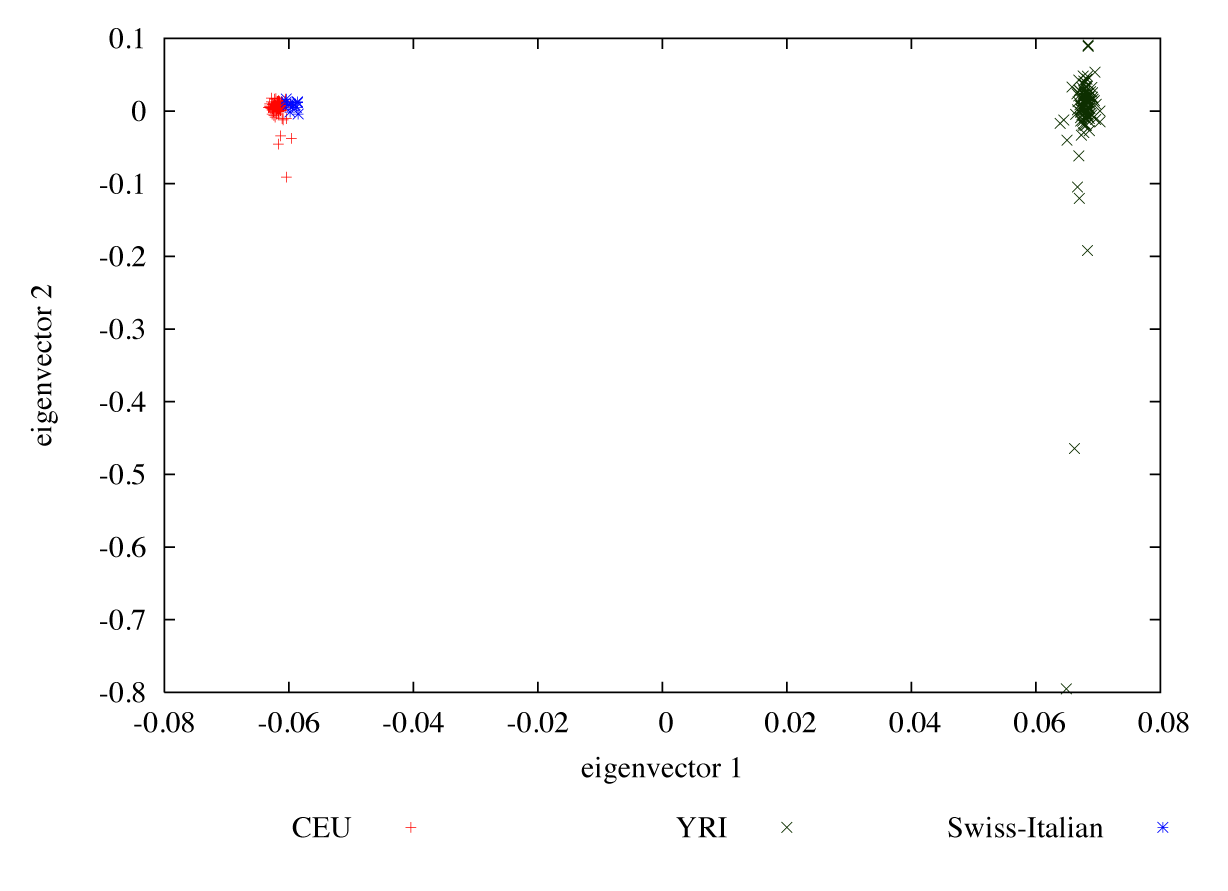
**

**Combined PCA analysis for all Italian populations:**

**a. Before outlier removal b. After outlier removal and relabeling**

**
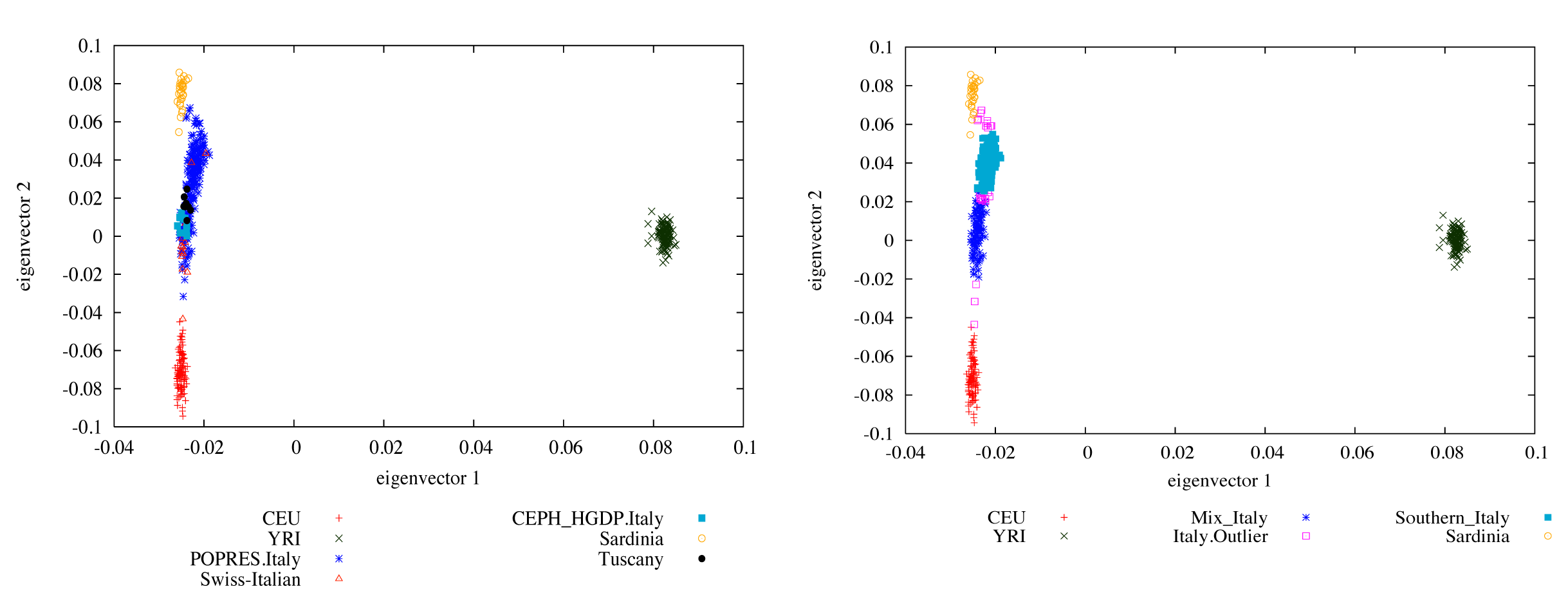
**

**D. Levant**

**
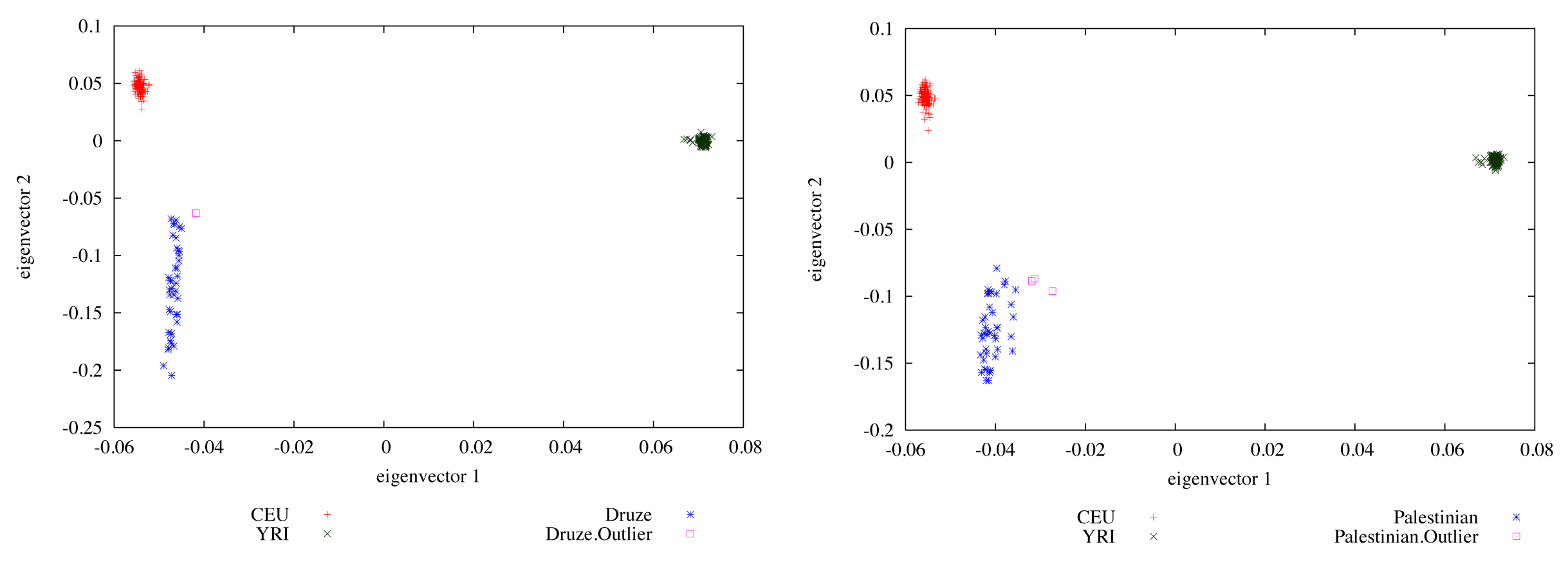
**

**
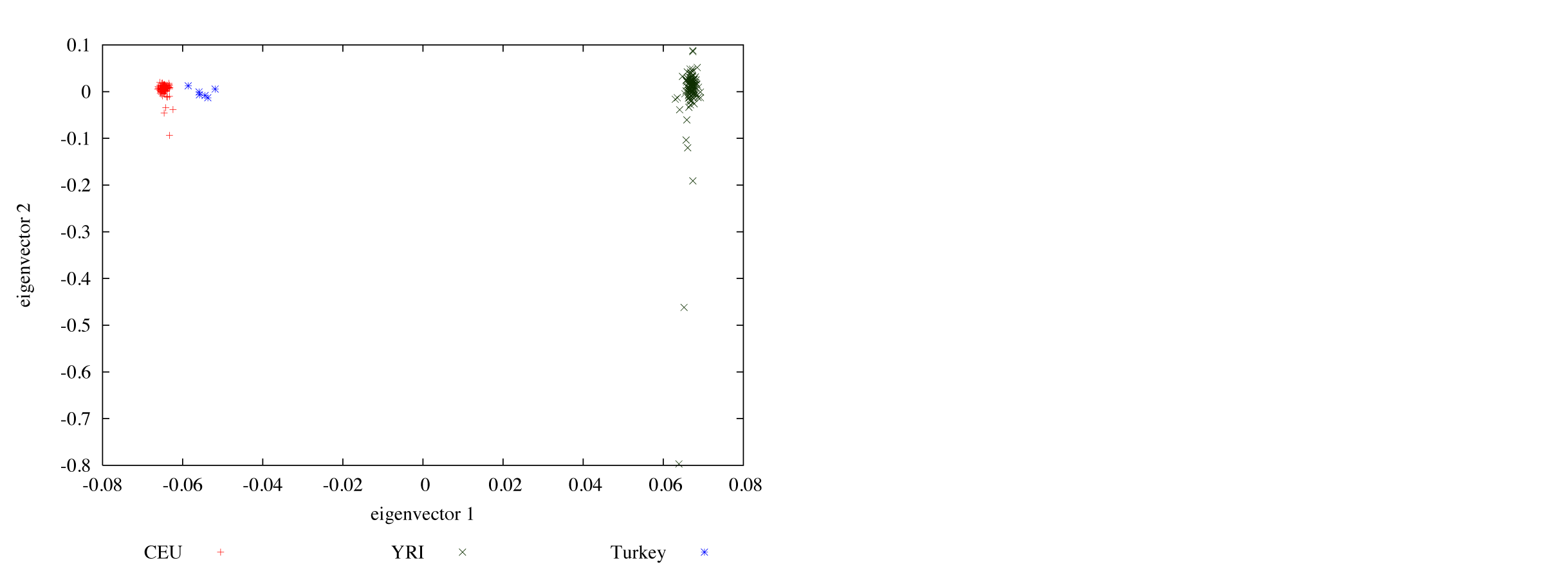
**

**Bedouin**

**a. Before outlier removal b. PCA-based classification**

**
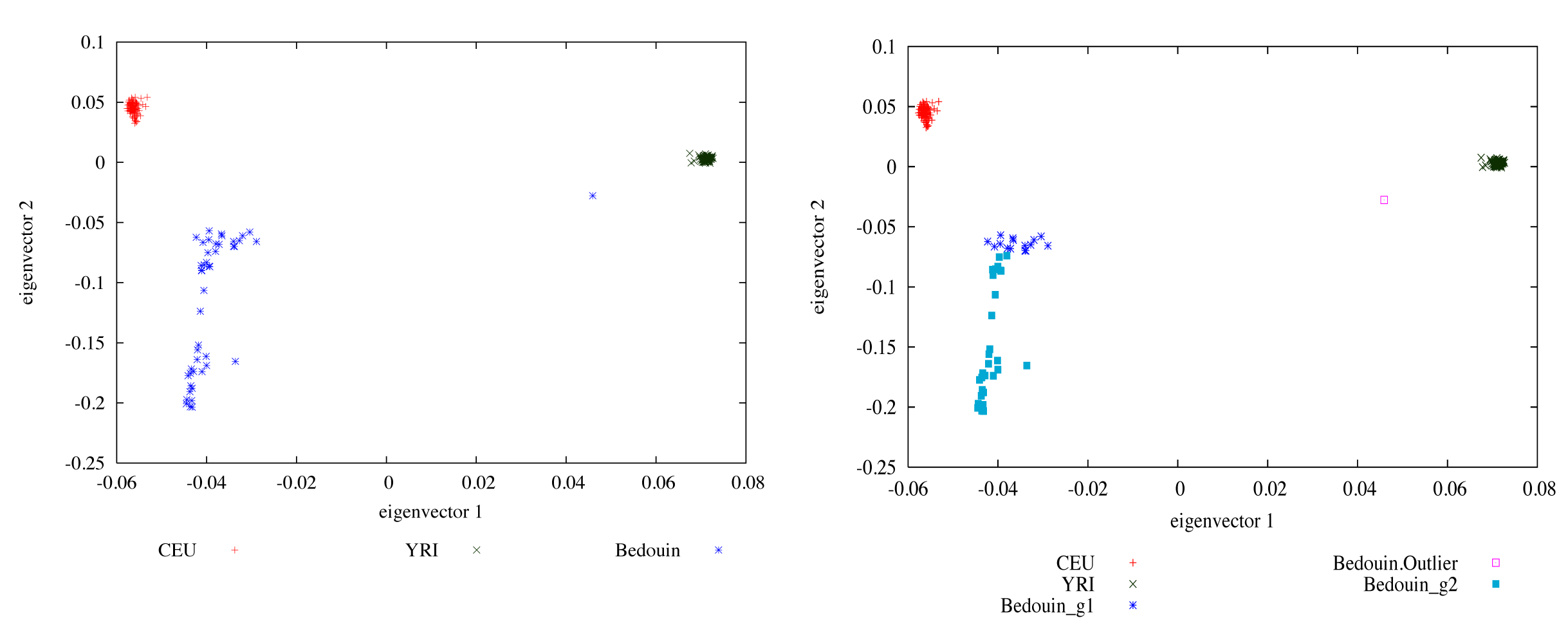
**

**E. Jewish Groups**

**IBD.Ashkenazi Jews**

**a. Before outlier removal b. PCA-based classification**

**
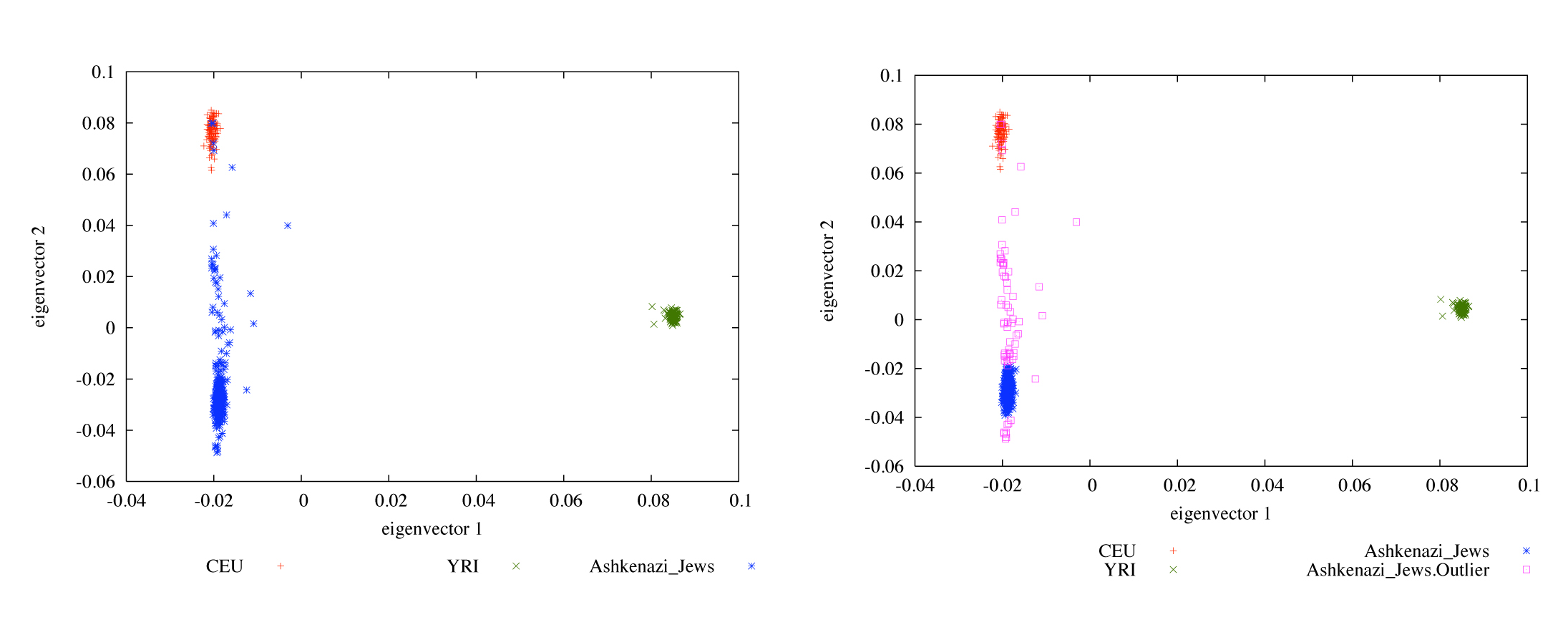
**

**Jewish HapMap Project**

**Combined PCA analysis for all Jewish groups:**

**a. Before outlier removal b. After outlier removal**

**
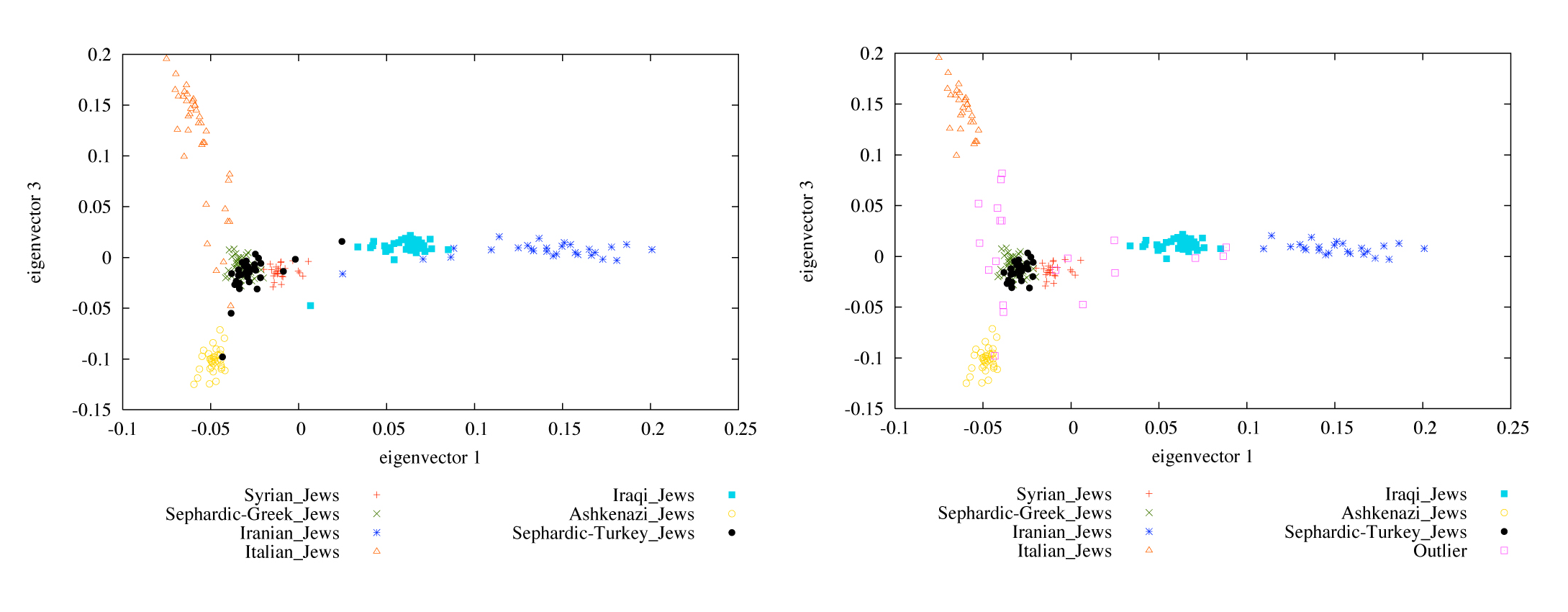
**
