# Supplementary figures and images for: The History of African Gene Flow into Southern Europeans, Levantines, and Jews
Source: PLoS Genet. 2011 Apr 21;7(4):e1001373. doi: 10.1371/journal.pgen.1001373 (PMC3080861; doi:10.1371/journal.pgen.1001373)

**Figure S2.** **PCA Projection with Adygei and Kenyan Bantu**


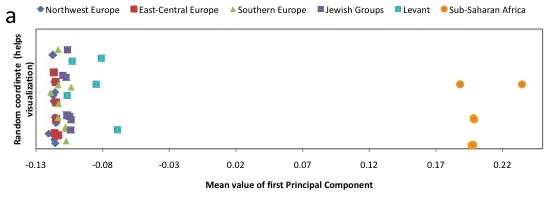

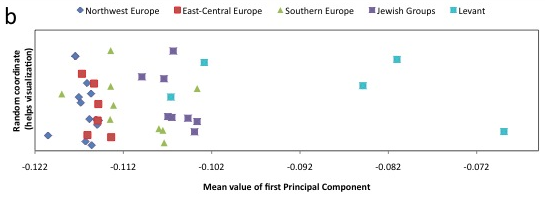

Supplement: Figure S2 — PCA Projection with Adygei and Kenyan Bantu. PCA was performed using genome-wide SNP data from Adygei and Kenyan Bantu. All West Eurasians populations with samples sizes greater than or equal to 5 were then projected onto these PCs. (a) The first panel presents data for all populations, (b) while the second provides a higher resolution view of West Eurasians after removing Sub-Saharan Africans. Each point on this graph indicates the mean value of the first PC for a projected population and West Eurasians populations are colored by 5 regional groupings-“Northwest Europe”, “East-Central Europe”, “Southern Europe”, “Levant”, “Jewish Groups”-with the assignments of populations to groups as shown in Table 1. The grouping “Sub-Saharan Africa” refers to six populations from the HGDP-CEPH panel: Kenyan Bantu, South African Bantu, Mandenka, Mbuti Pygmy, Biaka Pygmy and Yoruba. A qualitatively similar pattern is seen as in Figure 1. (0.13 MB DOC) [file pgen.1001373.s002.doc]

**Figure S3. Formal Tests of admixture.**

*4 Population Test*


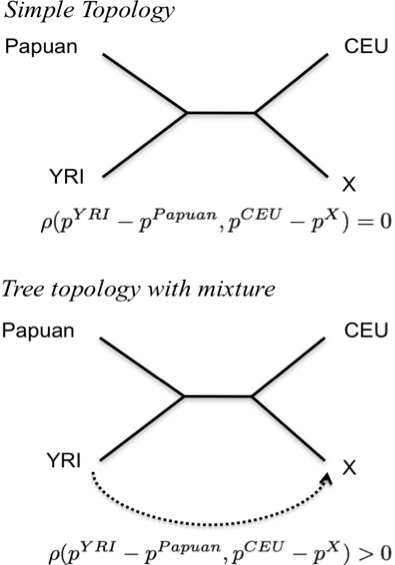


*3 Population Test*


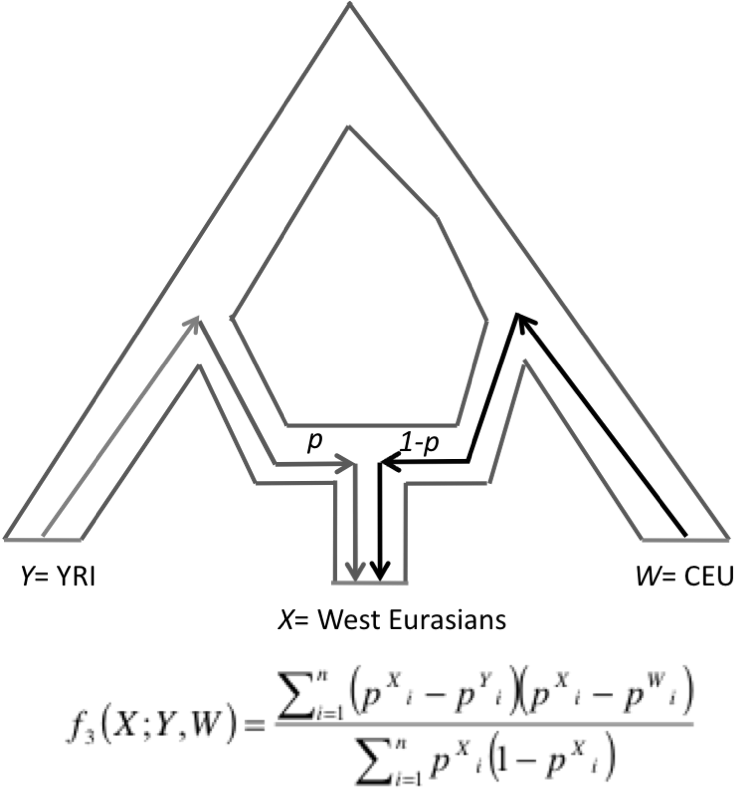

Supplement: Figure S3 — Formal tests of admixture. (1.73 MB DOC) [file pgen.1001373.s003.doc]

**Figure S5. Estimation of African ancestry using STRUCTURE.**

**
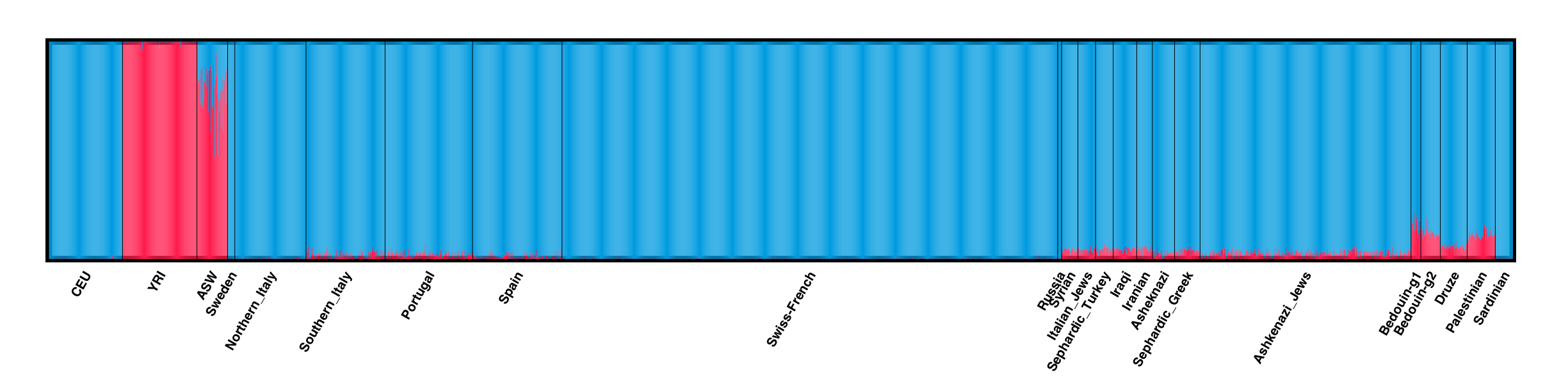
**

Supplement: Figure S6 — Estimation of African ancestry using STRUCTURE. We applied STRUCTURE 2.2 to estimate the mixture proportions using ∼13,900 markers (selected to not be in LD with each other) and K = 2. Each individual is represented by a single line with the length of the different colors reflecting the individual ancestry proportions. (0.18 MB DOC) [file pgen.1001373.s006.doc]

**Figure S7. *ROLLOFF* simulations for a scenario similar to African Americans**

**
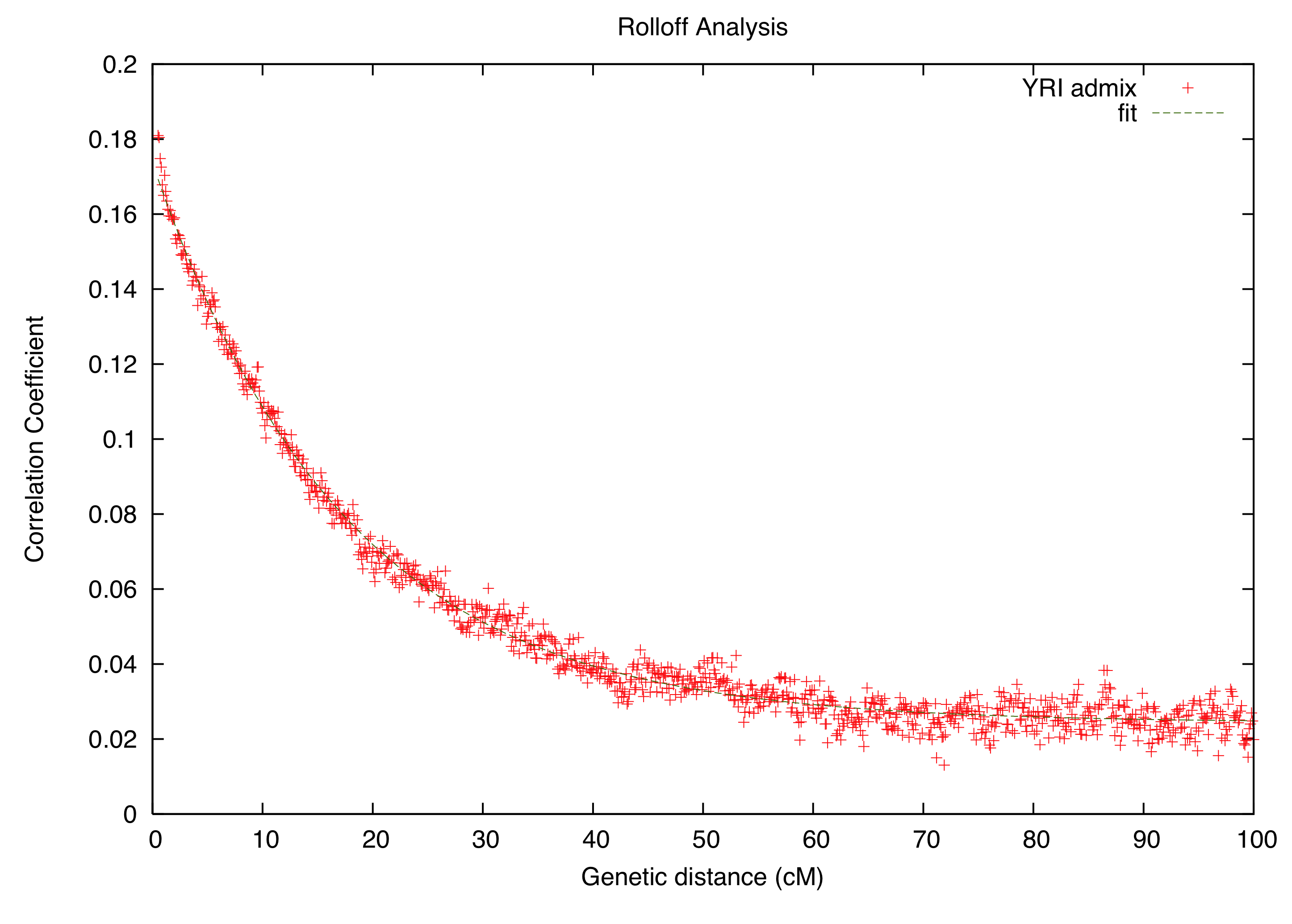
**

Supplement: Figure S7 — ROLLOFF simulation for a scenario similar to African Americans. We constructed genomes of 10 individuals with mixed European and African ancestry. We set the time since mixture (λ) at 6 generations and the European ancestry proportion (θ) was sampled from a beta distribution with mean 20% and standard deviation 10%. We performed ROLLOFF analysis with a non-overlapping dataset of European Americans and Yoruba Nigerians as reference populations. We plot the decay of weighted correlation coefficient as a function of genetic distance and estimate the date of admixture as 6±1 generations, by fitting an exponential distribution to the data. (0.43 MB DOC) [file pgen.1001373.s007.doc]

**Figure S11. *ROLLOFF* analysis in cases of no gene flow related to the tested ancestral populations.**


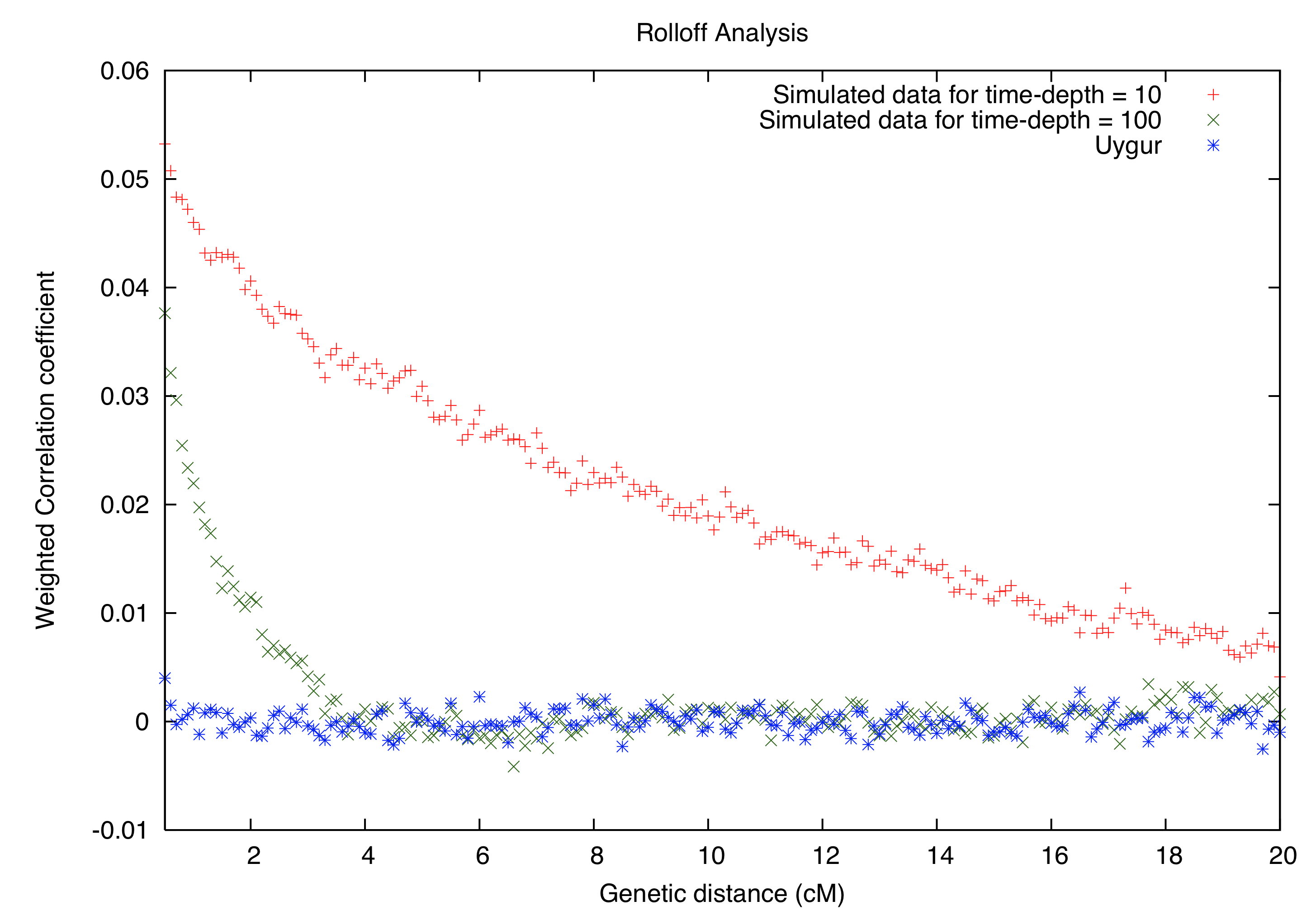

Supplement: Figure S8 — ROLLOFF analysis in cases of no gene flow related to the tested ancestral populations. We performed ROLLOFF analysis for East Asian Uygurs, who have both West Eurasians and East Eurasian ancestry. We used YRI and Pygmies (Mbuti and Biaka Pygmies) as the reference populations in ROLLOFF and saw no evidence of mixture. To show that this is not because of an inability to detect mixture when YRI and Pygmy-related groups are the true ancestral populations, we simulated 10 individuals of mixed Pygmy and Yoruba ancestry, with Yoruba mixture proportion (θ) = 80% and time since mixture (λ) = 10 generations (10 individuals) and θ = 80% and λ = 100 generations (10 individuals). We plot the ROLLOFF weighted correlation coefficient against genetic distance and observe clear evidence of mixture in these samples, with fairly accurately estimated dates of 10 and 90 generations respectively. (0.48 MB DOC) [file pgen.1001373.s008.doc]

**Figure S8. *ROLLOFF* analysis for double admixture event.**

**
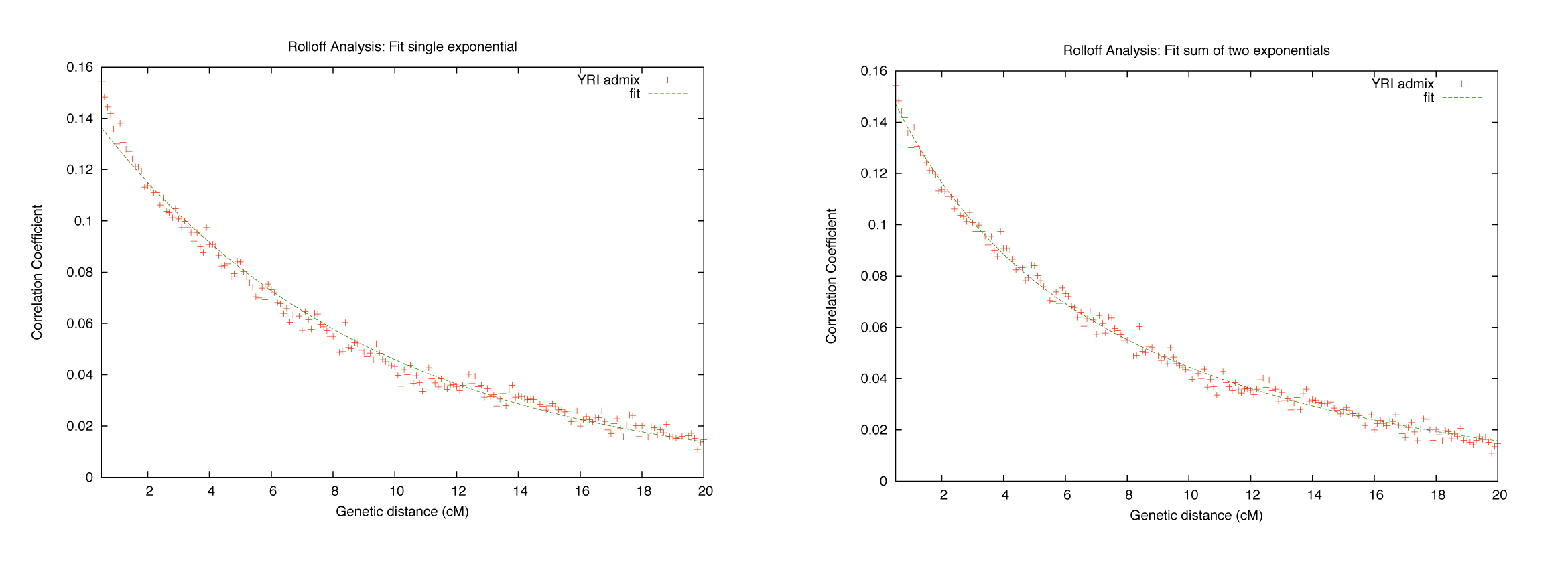
**

Supplement: Figure S9 — ROLLOFF analysis for double admixture event. We simulated double admixture scenarios (two events of gene flow) in which a 50%/50% mixture of CEU and YRI mixture occurred at λ = 30 generations, followed by a 50%/50% mixture of that admixed population and YRI at λ = 10 generations. We performed ROLLOFF analysis using a non-overlapping dataset of Yoruba Nigerians and European Americans as reference populations. In the left panel, we fit a single exponential distribution to the output and estimate the date of the admixture event as 11 generations. In the right panel, we fit a sum of two exponentials and estimate the dates of admixture as 35 and 9 generations. In both cases, we accurately estimate the date of the most recent mixture event. (0.23 MB DOC) [file pgen.1001373.s009.doc]

**Figure S9. A demographic model for continuous admixture scenarios.**


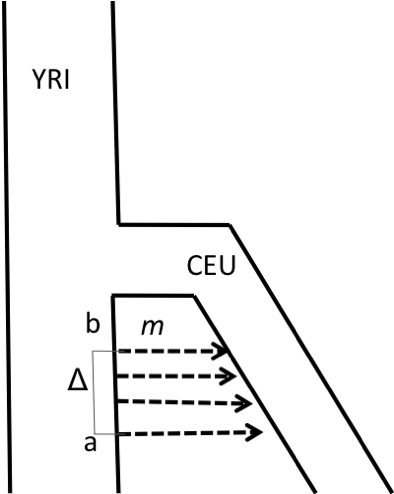

Supplement: Figure S10 — A demographic model for continuous admixture. To test the performance of ROLLOFF under continuous admixture scenarios, we simulate data for individuals with mixed ancestry using data for two ancestral populations CEU and YRI, where the gene flow occurs in an interval I = [a,b] where 0 < a ≤ b and the time is in generations. In each generation during I, we allow a proportion m (computed based on mixture proportion (θ)) of YRI lineages to migrate, yielding a total of 20% average African ancestry in the resulting admixed samples. (0.06 MB DOC) [file pgen.1001373.s010.doc]

**Figure S12. Establishment of the axes of variation within Africa using PCA.**


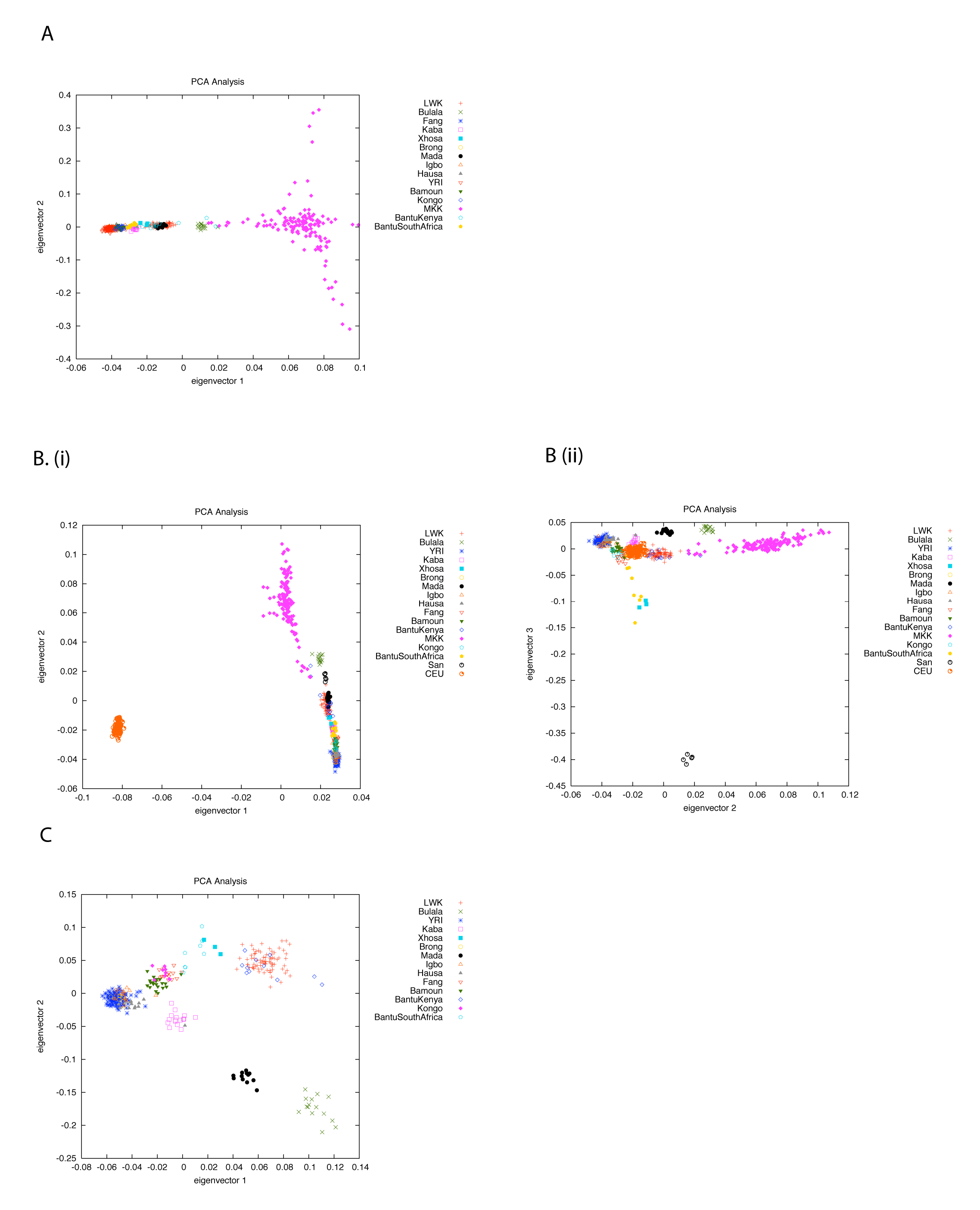

Supplement: Figure S12 — Establishment of the axes of variation within Africa using PCA. To study the relationship of sub-Saharan African populations to each other and filter out populations with West Eurasian ancestry, we performed the following three analyses: (A) PCA of 15 sub-Saharan African groups using EIGENSOFT (B) PCA of 15 sub-Saharan African groups along with HapMap Chinese (CHB) and San Bushmen, and (C) PCA of 14 sub-Saharan African groups (excluding Kenyan Maasai). (0.38 MB DOC) [file pgen.1001373.s012.doc]
