# Supplementary material for: The History of African Gene Flow into Southern Europeans, Levantines, and Jews
Source: PLoS Genet. 2011 Apr 21;7(4):e1001373. doi: 10.1371/journal.pgen.1001373 (PMC3080861; doi:10.1371/journal.pgen.1001373)
Supplement: Figure S4 — Demographic model to test the effect of ascertainment bias on 3 Pop. Test. We performed coalescent simulations using Hudson's ms [1] to generate data for two ancestral populations, Population A and Population B. For the simulation, we use a two-population demography where the effective population size of Pop A is N0 = 10,000 and the effective population size of Pop B varies from 0.25N0 to 0.85N0 such that the frequency differentiation FST(A,B) = 0.15 and the divergence time varies from 45,000–100,000 years. Using data for Population A and B, we create Population C where individuals have mixed Population A and B ancestry. We set the mixture proportion to be 80%/20% and the time since mixture to be 10 generations. (1.32 MB DOC) [file pgen.1001373.s004.doc]

**Figure S4. Demographic model to test the effect of ascertainment bias on *3 Population Test*.**


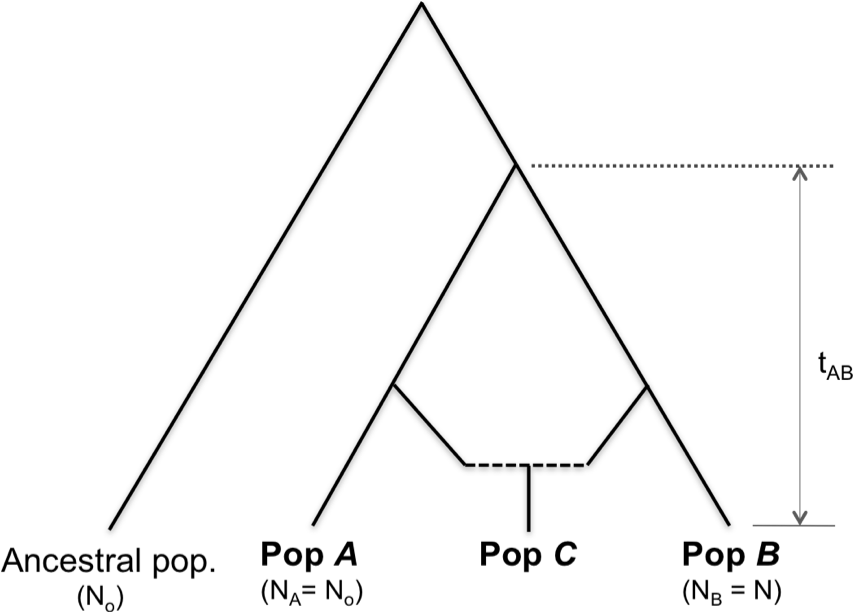


NOTE: Simulation framework was adapted from reference [1] where the authors were simulating data to test effect of SNP ascertainment bias on calculation of F-statistics similar to *3 Population Test* statistics.

***References:***

1. Sun J, Mullikin J, Patterson N, Reich D (2009) Microsatellites are molecular clocks that support accurate inferences about history. Molecular biology and evolution 26: 1017.
