# Supplementary material for: The History of African Gene Flow into Southern Europeans, Levantines, and Jews
Source: PLoS Genet. 2011 Apr 21;7(4):e1001373. doi: 10.1371/journal.pgen.1001373 (PMC3080861; doi:10.1371/journal.pgen.1001373)
Supplement: Figure S5 — Geographic gradient of African ancestry in Europeans. Sub-Saharan African ancestry proportions were estimated using f4 Ancestry Estimation. Populations in grey are estimated to have sub-Saharan African ancestry between 1–4%. The * in Switzerland indicates that the three populations available from this country have variable estimates: Swiss-Germans show no evidence of African mixture, Swiss-French 0.5±0.2% and Swiss-Italians 1.6±0.2%. The ‘+’ sign in Italy indicates that multiple samples were available but all show evidence of African mixture. No data are available from countries filled with diagonal lines. The map was downloaded from- http://www.ecozon.com/images/europe_map.jpg (0.22 MB DOC) [file pgen.1001373.s005.doc]

**Figure S6. Geographic gradient of African ancestry in Europeans.**

**
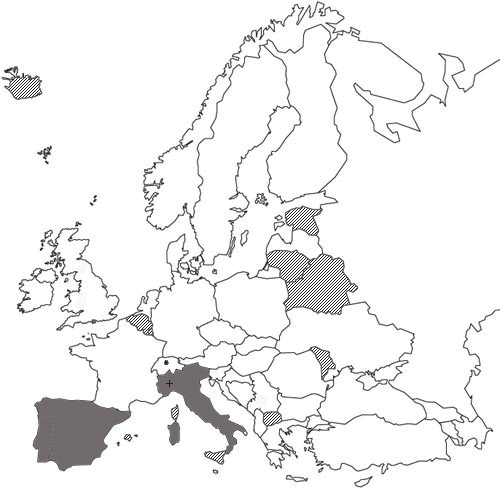
**

NOTE: The map was downloaded from- <http://www.ecozon.com/images/europe_map.jpg>
