# Supplementary material for: The History of African Gene Flow into Southern Europeans, Levantines, and Jews
Source: PLoS Genet. 2011 Apr 21;7(4):e1001373. doi: 10.1371/journal.pgen.1001373 (PMC3080861; doi:10.1371/journal.pgen.1001373)
Supplement: Figure S11 — ROLLOFF analysis for West Eurasians. We performed ROLLOFF analysis for each West Eurasian population X that showed significant evidence of admixture in the 4 Population Test using YRI and CEU as reference populations. We plot the decay of admixture LD as a function of genetic distance and estimate the date of admixture by fitting an exponential distribution to the data. Standard errors were calculated using a Weighted Block Jackknife as described in the Materials and Methods. (1.52 MB DOC) [file pgen.1001373.s011.doc]

**Figure S10. *ROLLOFF* analysis for West Eurasians.**

**A. Northwest Europe**

**
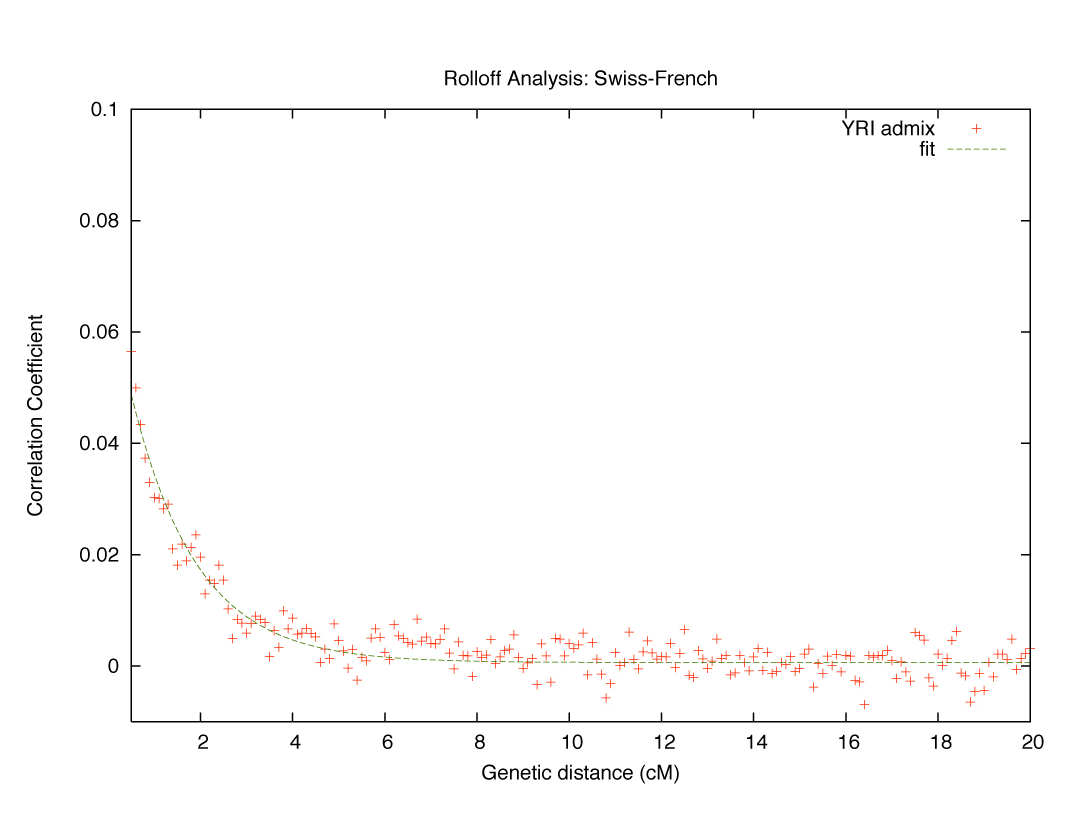
**

**B. Southern Europe**

**
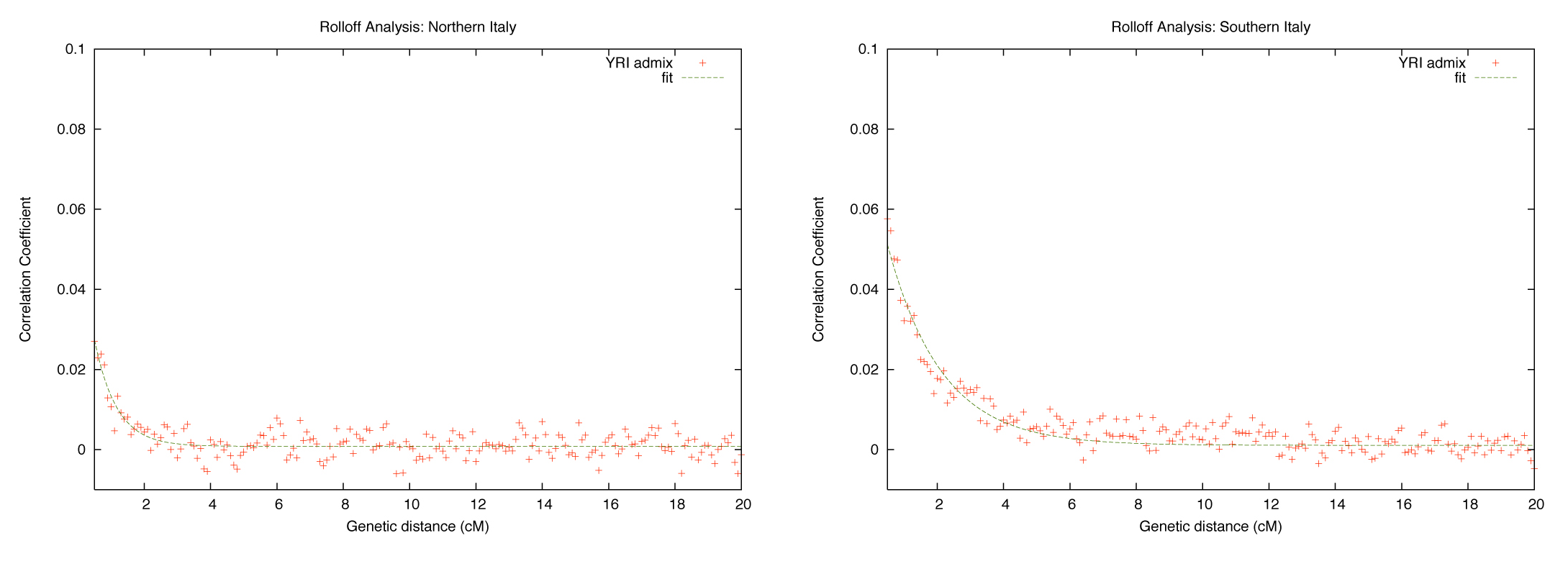
**


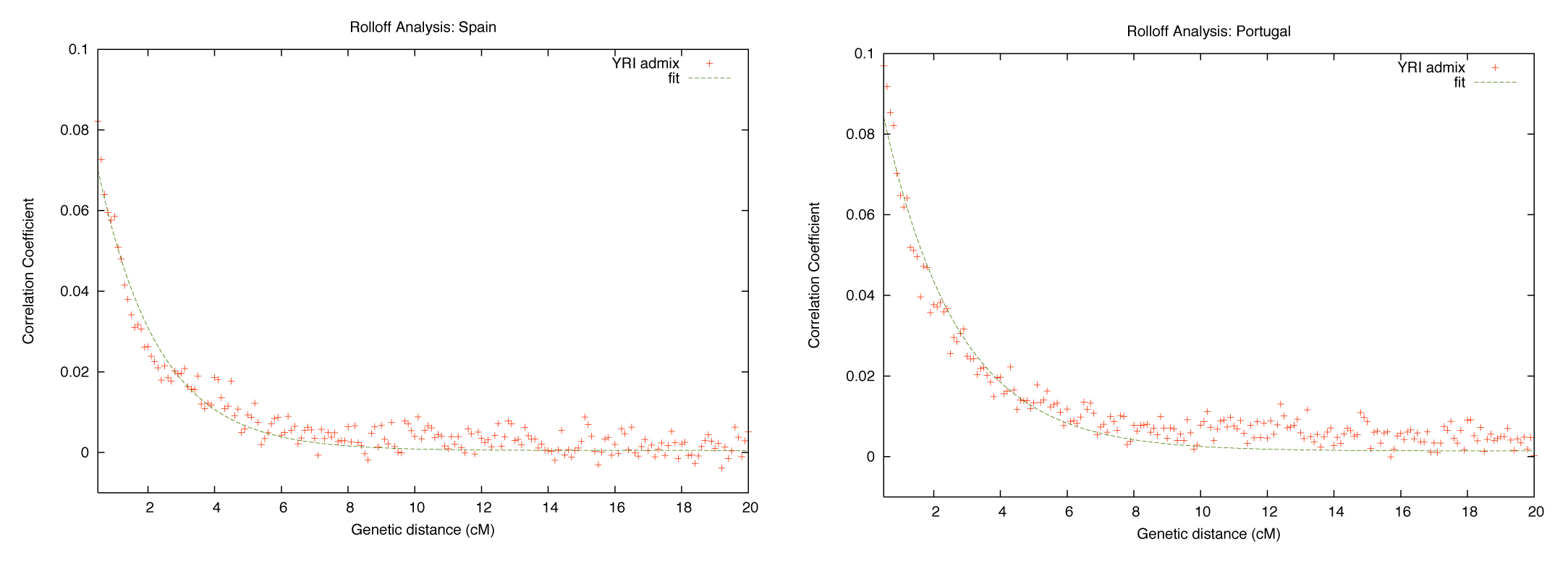


**C. Levantine Populations**

**
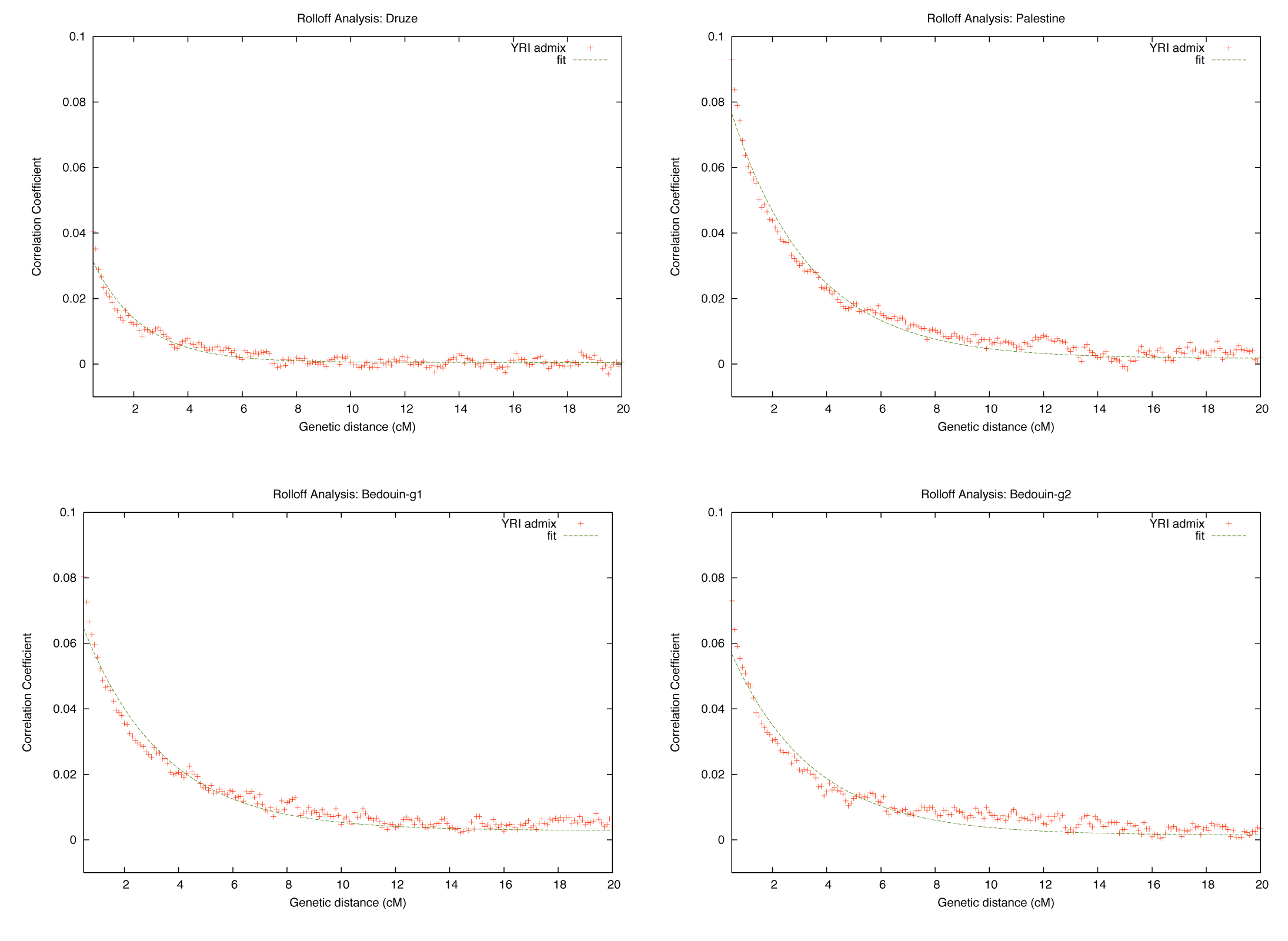
**

**D. Jewish Groups**

**
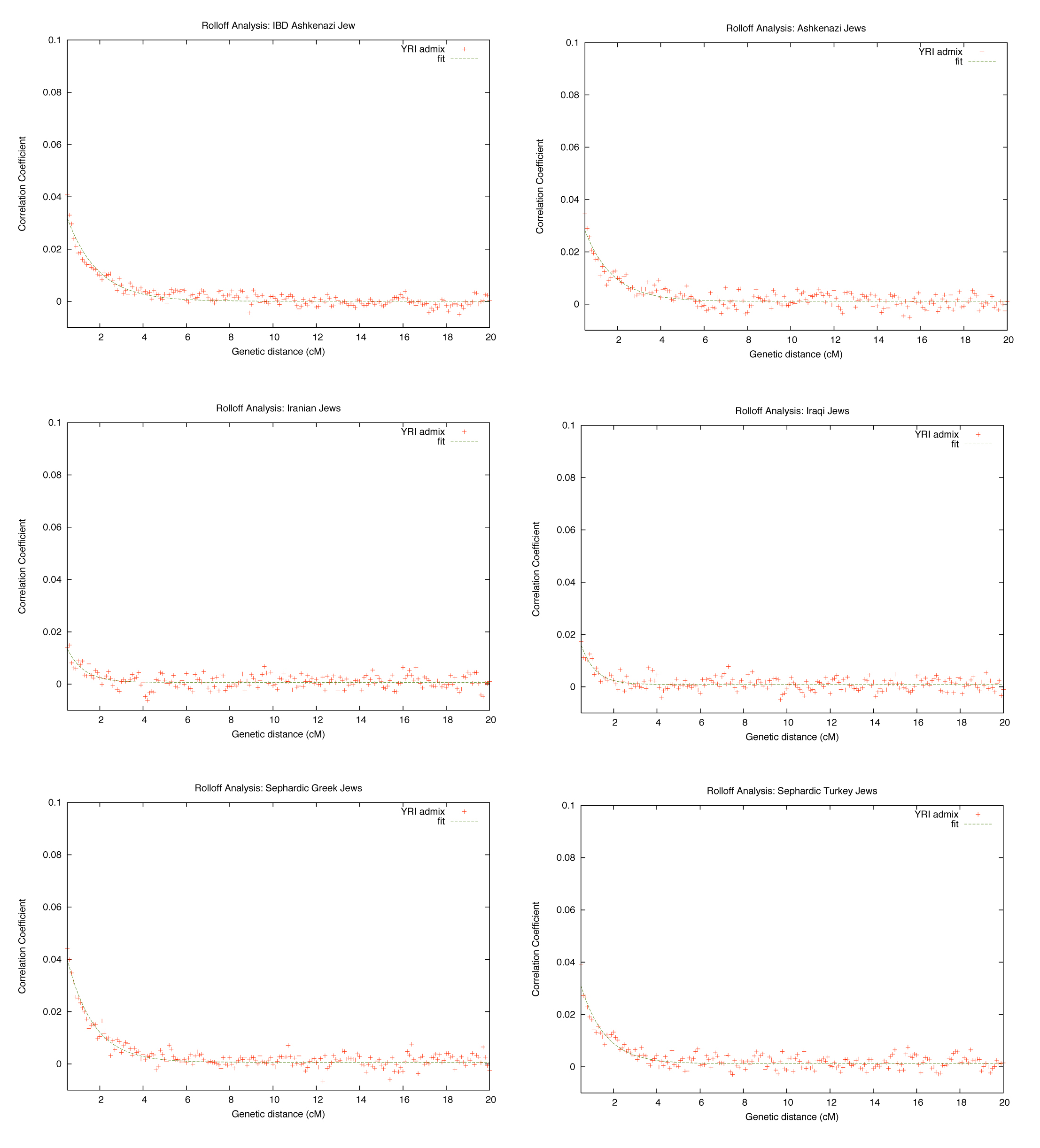
**

**
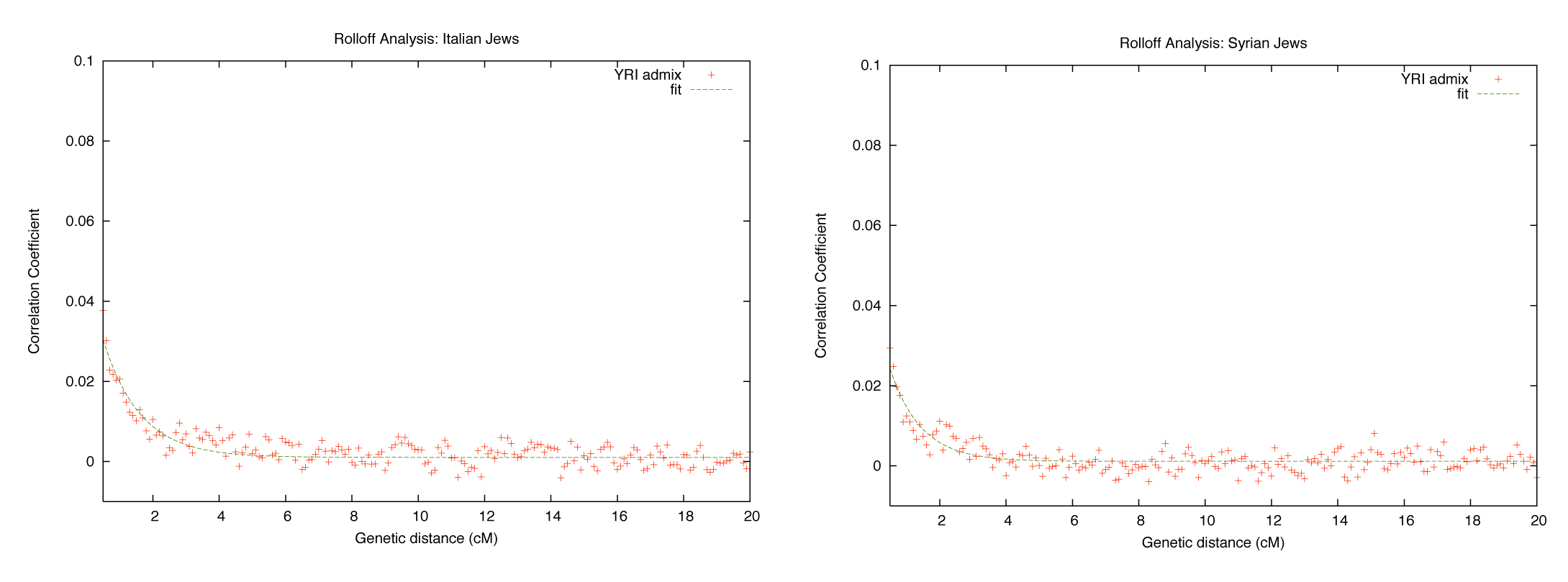
**
