# Supplementary material for: The History of African Gene Flow into Southern Europeans, Levantines, and Jews
Source: PLoS Genet. 2011 Apr 21;7(4):e1001373. doi: 10.1371/journal.pgen.1001373 (PMC3080861; doi:10.1371/journal.pgen.1001373)

**Figure S13. Source of African ancestry in West Eurasians is likely to include some East African ancestors.**


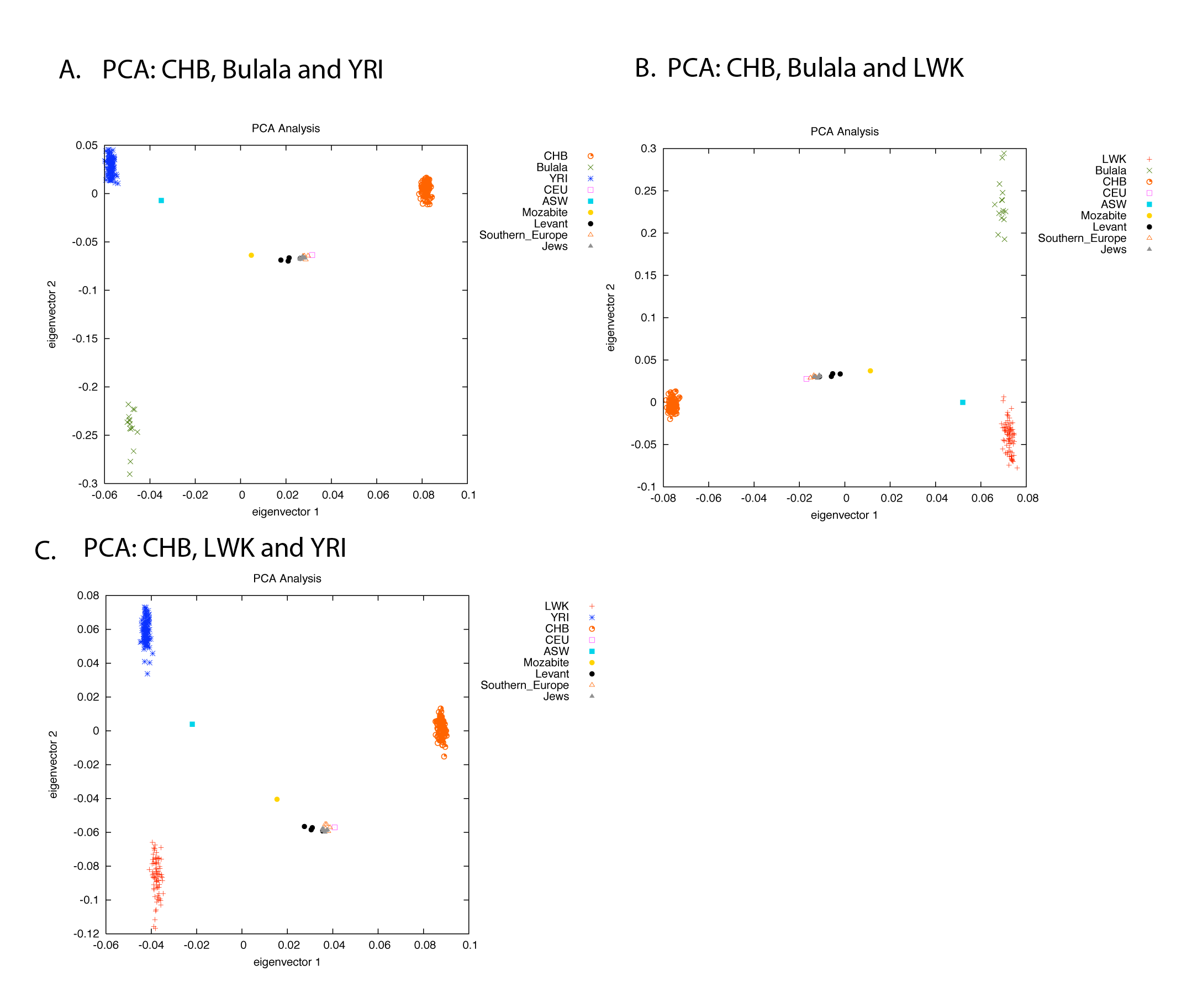

Supplement: Figure S13 — Source of African ancestry in West Eurasians may include some East African ancestors. In order to identify the source of the African ancestry in Levantines, Southern Europeans and Jews, we performed PCA Projection with all three possible pairs of African populations (Bulala, Kenyan Luhya (LWK) and Yoruba (YRI)) along with HapMap Chinese (CHB), and then plotted the mean values of all the samples from each West Eurasian population, African Americans (ASW) and North African (Mozabites) onto the first PC and second PC. These admixed West Eurasian populations align along a gradient that points more at (a) YRI than Bulala, and (b) LWK than Bulala suggesting little evidence for Chadic ancestry in West Eurasians, (c) The PCA suggests more relatedness to LWK than to YRI, suggesting that there may be some East African related ancestry in these West Eurasian populations. (0.23 MB DOC) [file pgen.1001373.s013.doc]
