# Supplementary material for: The History of African Gene Flow into Southern Europeans, Levantines, and Jews
Source: PLoS Genet. 2011 Apr 21;7(4):e1001373. doi: 10.1371/journal.pgen.1001373 (PMC3080861; doi:10.1371/journal.pgen.1001373)
Supplement: Table S1 — Summary of datasets. (0.06 MB DOC) [file pgen.1001373.s014.doc]

***Table S1. Summary of Datasets***

| **Merged Dataset** | **Dataset included** | **#Inds** | **#SNPs** | **Analyses in which this data set is used** |
| --- | --- | --- | --- | --- |
| A | POPRES | 3,845 | ~500,000 |  |
| B | HGDP-CEPH | 940 | ~650,000 |  |
| C | HapMap 3 | 1,115 | >1M |  |
| D | IBD | 392 | ~300,000 |  |
| E | Jewish HapMap | 232 | ~1M |  |
| F | POPRES + HapMap3 | 4,960 | 347,315 | *3 Population Test* |
|  |  |  |  | *ROLLOFF* Analysis |
|  |  |  |  | *PCA Analysis* |
| G | HGDP-CEPH + HapMap3 | 2,055 | 606,071 | *3 Population Test* |
|  |  |  |  | *ROLLOFF* Analysis |
|  |  |  |  | *PCA Analysis* |
| H | IBD + HapMap3 | 1,507 | 284,951 | *3 Population Test* |
|  |  |  |  | *ROLLOFF* analysis |
|  |  |  |  | *PCA Analysis* |
| I | Jewish HapMap +HapMap3 | 1,347 | 466,580 | *3 Population Test* |
|  |  |  |  | *ROLLOFF* analysis |
|  |  |  |  | *PCA Analysis* |
| J | POPRES + HGDP-CEPH + HapMap3 | 5,900 | 85,628 | *4 Population Test* |
|  |  |  |  | *f_4_ Ancestry Estimation* |
| K | IBD + HGDP-CEPH + HapMap3 | 2,447 | 284262 | *4 Population Test* |
|  |  |  |  | *f_4_ Ancestry Estimation* |
| L | Jewish HapMap +HapMap3 + HGDP-CEPH | 2,287 | 118,364 | *f_4_ Ancestry Estimation* |
|  |  |  |  | *4 Population Test* |
| M | Jewish HapMap +HapMap3 + HGDP-CEPH + IBD + POPRES | 3,614 | 36,175 | *PCA Projection* |
