# Supplementary material for: The History of African Gene Flow into Southern Europeans, Levantines, and Jews
Source: PLoS Genet. 2011 Apr 21;7(4):e1001373. doi: 10.1371/journal.pgen.1001373 (PMC3080861; doi:10.1371/journal.pgen.1001373)
Supplement: Table S2 — Outlier samples removed based on PCA curation. (0.04 MB DOC) [file pgen.1001373.s015.doc]

***Table S2. Outlier samples removed based on PCA curation***

| **Population (X)** | **Dataset** | **Number of outliers removed** |
| --- | --- | --- |
| Bedouin | HGDP-CEPH | 1 |
| Swiss-Italian | POPRES | 3 |
| Swiss-French | POPRES | 1 |
| Palestine | HGDP-CEPH | 3 |
| Druze | HGDP-CEPH | 1 |
| Germany | POPRES | 1 |
| Croatia | POPRES | 2 |
| Greece | POPRES | 8 |
| Italy* | POPRES | 24 |
| Sardinia | POPRES | 1 |
| Tuscany | POPRES | 1 |
| Ashkenazi Jews* | IBD | 69 |
| Iraqi Jews | Jewish HapMap | 1 |
| Iranian Jews | Jewish HapMap | 4 |
| Italian Jews | Jewish HapMap | 10 |
| Sephardic Greek Jews | Jewish HapMap | 7 |
| Sephardic Turkey Jews | Jewish HapMap | 3 |

* Due to evidence of population sub-structure, many individuals were excluded from these populations so that we were left with populations that were homogeneous in PCA.
