# Supplementary material for: The History of African Gene Flow into Southern Europeans, Levantines, and Jews
Source: PLoS Genet. 2011 Apr 21;7(4):e1001373. doi: 10.1371/journal.pgen.1001373 (PMC3080861; doi:10.1371/journal.pgen.1001373)
Supplement: Table S3 — Comparison of test statistics before and after PCA-based curation. (0.09 MB DOC) [file pgen.1001373.s016.doc]

***Table S3. Comparison of test statistics before and after PCA-based curation***

| **Population (X)** | **Dataset** | ***Before data cleaning*** | | | ***After data cleaning*** | | |
| --- | --- | --- | --- | --- | --- | --- | --- |
|  |  | **Sam-ples (N)** | **West African ancestry proportion ± standard error** | **Estimated date of admixture (generations) ± standard error** | **Sam-ples (n)** | **West African ancestry proportion ± standard error** | **Estimated date of admixture (generations) ± standard error** |
| African American | HapMap3 | 49 | 79.4% ± 0.3% | 6 ± 1 | 49 | 79.4% ± 0.3% | 6 ± 1 |
| Palestinian | HGDP-CEPH | 46 | 10.1% ± 0.4% | 29 ± 2 | 43 | 9.3% ± 0.4% | 34 ± 2 |
| Bedouin | HGDP-CEPH | 46 | 13.0% ± 0.4% | 33 ± 4 | -- | -- | -- |
| Bedouin-g1* | HGDP-CEPH | -- | -- | -- | 15 | 14.5% ± 0.4% | 34 ± 3 |
| Bedouin-g2* | HGDP-CEPH | -- | -- | -- | 30 | 10.1% ± 0.4% | 33 ± 3 |
| Druze | HGDP-CEPH | 41 | 4.2% ± 0.4% | 53 ± 6 | 40 | 4.4% ± 0.4% | 54 ± 7 |
| Spain | POPRES | 137 | 2.4% ± 0.3% | 55 ± 3 | 137 | 2.4% ± 0.3% | 55 ± 3 |
| Portugal | POPRES | 134 | 3.2% ± 0.3% | 45 ± 5 | 134 | 3.2% ± 0.3% | 45 ± 5 |
| Italy | POPRES | 225 | 2.2% ± 0.3% | 68 ± 7 | -- | -- | -- |
| Swiss-Italian | POPRES | 13 | 1.6% ± 0.5% | 117 ± 41 | -- | -- | -- |
| Sardinian | HGDP-CEPH | 28 | 2.9% ± 0.4% | 95 ± 26 | 27 | 2.9% ± 0.4% | 96 ± 28 |
| Bergamo | HGDP-CEPH | 12 | 1.6% ± 0.4% | 214 ± 58 | -- | -- | -- |
| Tuscan | HGDP-CEPH | 8 | 1.3% ± 0.4% | 81 ± 62 | -- | -- | -- |
| Southern-Italy* | POPRES | -- | -- | -- | 121 | 2.7% ± 0.3% | 62 ± 6 |
| Northern-Italy* | POPRES | -- | -- | -- | 90 | 1.1% ± 0.3% | 154 ± 27 |
| Swiss-French | POPRES | 760 | 0.5% ± 0.2% | 71 ± 5 | 759 | 0.5% ± 0.2% | 71 ± 6 |
| Ashkenazi Jews | IBD | 392 | 2.8% ± 0.3% | 54 ± 8 | 323 | 2.8% ± 0.3% | 91 ± 11 |
| Ashkenazi Jews | Jewish HapMap | 34 | 3.2% ± 0.4% | 76 ± 13 | 34 | 3.2% ± 0.4% | 76 ± 13 |
| Syrian Jews | Jewish HapMap | 25 | 3.9% ± 0.5% | 99 ± 23 | 25 | 3.9% ± 0.5% | 99 ± 23 |
| Iranian Jews | Jewish HapMap | 28 | 2.4% ± 0.6% | 127 ± 44 | 24 | 2.6% ± 0.6% | 129 ± 34 |
| Iraqi Jews | Jewish HapMap | 37 | 3.8% ± 0.5% | 155 ± 20 | 36 | 3.8% ± 0.5% | 153 ± 22 |
| Sephardic Greek Jews | Jewish HapMap | 39 | 4.8% ± 0.4% | 82 ± 8 | 39 | 4.8% ± 0.4% | 82 ± 8 |
| Sephardic Turkey Jews | Jewish HapMap | 32 | 4.1% ± 0.4% | 84 ± 11 | 27 | 4.5% ± 0.4% | 89 ± 11 |
| Italian Jews | Jewish HapMap | 37 | 4.7% ± 0.5% | 92 ± 18 | 27 | 4.9% ± 0.5% | 88 ± 19 |

Note: ‘*‘ indicates that new population label based on PCA curation. Mixture proportion estimates are based on *f_4_ Ancestry Estimation* using San, Yoruba, CEU and Papuan as the reference populations. The *ROLLOFF* estimated date of mixture uses CEU and YRI as the proposed ancestral populations
