# Supplementary material for: The History of African Gene Flow into Southern Europeans, Levantines, and Jews
Source: PLoS Genet. 2011 Apr 21;7(4):e1001373. doi: 10.1371/journal.pgen.1001373 (PMC3080861; doi:10.1371/journal.pgen.1001373)
Supplement: Table S4 — 4 Pop. Test using alternate ancestral populations compared to Table 1. (0.10 MB DOC) [file pgen.1001373.s017.doc]

***Table S4. 4 Pop. Test using different ancestral pops. compared to Table 1***

| **Population (*X*)** | **Region** | **Dataset** | **Z-score for *4 Population test*** | | |
| --- | --- | --- | --- | --- | --- |
|  |  |  |  |  |  |
|  |  |  |  |  |  |
|  |  |  | **(P_Papuan_-P_YRI_) × (P_Adygei_-P_X_)** | **(P_Papuan_-P_Mandenka_) × (P_CEU_-P_X_)** | **(P_Papuan_-P_BantuKenya_) × (P_CEU_-P_X_)** |
|  |  |  |  |  |  |
| African American | n/a | HapMap3 | **-85.6** | **-85.1** | **-77.7** |
| Palestine | L | HGDP-CEPH | **-28.1** | **-27.3** | **-27.8** |
| Turkey | L | POPRES | -2.2 | -1.4 | -1.4 |
| Bedouin-g1 | L | HGDP-CEPH | **-37.3** | **-35.6** | **-35.4** |
| Bedouin-g2 | L | HGDP-CEPH | **-25.7** | **-25.7** | **-26.0** |
| Druze | L | HGDP-CEPH | **-16.1** | **-14.5** | **-14.8** |
| Spain | SE | POPRES | **-8.0** | **-12.4** | **-11.7** |
| Portugal | SE | POPRES | **-10.0** | **-14.6** | **-14.4** |
| Romania | SE | POPRES | -2.0 | -1.0 | -0.9 |
| Croatia | SE | POPRES | -0.6 | 0.7 | 0.9 |
| Bosnia-Herzegovina | SE | POPRES | -1.8 | -0.5 | -0.5 |
| Sardinia | SE | HGDP-CEPH | **-9.3** | **-9.5** | **-10.0** |
| Southern-Italy | SE | POPRES | **-9.2** | **-11.2** | **-10.9** |
| Northern-Italy | SE | POPRES | **-5.0** | **-6.0** | **-5.8** |
| Austria | ECE | POPRES | -1.7 | -0.5 | -0.4 |
| Poland | ECE | POPRES | -0.7 | 1.4 | 1.2 |
| Hungary | ECE | POPRES | -1.5 | 0.1 | 0.4 |
| Czech Republic | ECE | POPRES | -1.1 | -0.3 | 0.6 |
| Adygei | ECE | HGDP-CEPH | -- | 3.1 | 2.7 |
| Russia | ECE | POPRES | -0.7 | 0.7 | 0.5 |
| Russia | ECE | HGDP-CEPH | -0.7 | 0.7 | 0.5 |
| Swiss-French | I | POPRES | **-3.3** | **-3.6** | **-3.5** |
| France | I | POPRES | -2.8 | -2.2 | -2.4 |
| France | I | HGDP-CEPH | -2.8 | -2.2 | -2.4 |
| Basque | I | HGDP-CEPH | -3.0 | -1.5 | -1.5 |
| Belgium | I | POPRES | -2.3 | -1.0 | -1.0 |
| Orkney | I | POPRES | -0.2 | **3.2** | 2.9 |
| United Kingdom | I | POPRES | -1.4 | 1.1 | 1.1 |
| Ireland | I | POPRES | -0.9 | 2.0 | 1.9 |
| Scotland | I | POPRES | 1.6 | 3.0 | 2.9 |
| Netherlands | I | POPRES | -0.9 | 1.0 | 0.8 |
| Swiss-German | I | POPRES | -2.5 | -1.5 | -1.3 |
| Germany | I | POPRES | -2.3 | -1.0 | -0.8 |
| Sweden | I | POPRES | -0.2 | 1.9 | 1.7 |
| Ashkenazi Jews | n/a | IBD | **-11.4** | **-11.7** | **-11.4** |
| Ashkenazi Jews | n/a | Jewish HapMap | **-8.8** | **-9.6** | **-9.5** |
| Syrian Jews | n/a | Jewish HapMap | **-10.1** | **-10.2** | **-10.0** |
| Iranian Jews | n/a | Jewish HapMap | **-6.6** | **-5.9** | **-5.7** |
| Iraqi Jews | n/a | Jewish HapMap | **-9.4** | **-8.8** | **-8.8** |
| Sephardic Greek Jews | n/a | Jewish HapMap | **-12.4** | **-13.8** | **-13.8** |
| Sephardic Turkey Jews | n/a | Jewish HapMap | **-11.9** | **-13.6** | **-13.5** |
| Italian Jews | n/a | Jewish HapMap | **-10.8** | **-11.3** | **-11.6** |

Note: We analyzed data from all West Eurasian populations with at least 5 samples. Regions are abbreviated as: I – Northwest Europe, ECE – East-Central Europe, SE – Southern Europe and L – Levant. For the *4 Population Test,* we report only results for the tree shown in the table. Results for all alternate topologies show even higher violations of the tree (|Z| >> 15). Scores that are significant are highlighted in bold.
