# Supplementary material for: The History of African Gene Flow into Southern Europeans, Levantines, and Jews
Source: PLoS Genet. 2011 Apr 21;7(4):e1001373. doi: 10.1371/journal.pgen.1001373 (PMC3080861; doi:10.1371/journal.pgen.1001373)
Supplement: Table S5 — Simulation to test the effect of ascertainment bias on 3 Pop. Test results. (0.04 MB DOC) [file pgen.1001373.s018.doc]

**Table S6. *Simulation to test the effect of ascertainment bias on 3 Pop. Test Results***

| **Model** | **Divergence time (t_AB_)** | **Effective pop. size of Pop *A* (N_A_) and Pop *B* (N_B_)** | ***3 Pop. Test***  ***(P_C_-P_A_)(P_C_-P_B_)*** |
| --- | --- | --- | --- |
| **1: One chromosome from each Pop *A* and Pop *B*** | 45,000 | N_A_ = N_o_, N_B_ = 0.25N_o_ | -48.8 |
|  | 60,000 | N_A_ = N_o_, N_B_ = 0.4N_o_ | -54.7 |
|  | 100,000 | N_A_ = N_o_, N_B_ = 0.85N_o_ | -37.3 |
| **2: Both chromosomes from Pop *A*** | 45,000 | N_A_ = N_o_, N_B_ = 0.25N_o_ | -68.6 |
|  | 60,000 | N_A_ = N_o_, N_B_ = 0.4N_o_ | -90.8 |
|  | 100,000 | N_A_ = N_o_, N_B_ = 0.85N_o_ | -45.1 |
| **3: Both chromosomes from Pop *B*** | 45,000 | N_A_ = N_o_, N_B_ = 0.25N_o_ | -40.8 |
|  | 60,000 | N_A_ = N_o_, N_B_ = 0.4N_o_ | -20.4 |
|  | 100,000 | N_A_ = N_o_, N_B_ = 0.85N_o_ | -40.6 |

NOTE: Details of the demographic model used for the simulation are shown in Figure S4. Effective population size of Pop *B* (N_B_) is set such that the F_ST_(A,B) = 0.15
