# Supplementary material for: The History of African Gene Flow into Southern Europeans, Levantines, and Jews
Source: PLoS Genet. 2011 Apr 21;7(4):e1001373. doi: 10.1371/journal.pgen.1001373 (PMC3080861; doi:10.1371/journal.pgen.1001373)
Supplement: Table S6 — f4 Ancestry Estimation using different ancestral populations compared to Table 2. (0.06 MB DOC) [file pgen.1001373.s019.doc]

***Table S5. f_4_ Ancestry Estimation*** using different ancestral populations compared to Table 2

| **Population (X)** | **Dataset** | **Region** | **(San, (Mandenka, (Papuan, (CEU,X))))** | **(San,(BantuKenya, (Papuan,(CEU,X))))** | **(San,(YRI, (CHB, (CEU,X))))** |
| --- | --- | --- | --- | --- | --- |
| African Americans | HapMap3 | n/a | 81.0% ± 0.3% | 82.3% ± 0.4% | 79.0% ± 0.2% |
| Palestinian | HGDP-CEPH | L | 9.5% ± 0.4% | 9.6% ± 0.4% | 10.3% ± 0.3% |
| Bedouin-g1 | HGDP-CEPH | L | 14.8% ± 0.4% | 15.1% ± 0.4% | 15.1% ± 0.3% |
| Bedouin-g2 | HGDP-CEPH | L | 10.3% ± 0.5% | 10.4% ± 0.5% | 11.0% ± 0.4% |
| Druze | HGDP-CEPH | SE | 4.5% ± 0.4% | 4.5% ± 0.4% | 5.2% ± 0.3% |
| Spain | POPRES | SE | 2.5% ± 0.3% | 3.3% ± 0.3% | 3.5% ± 0.2% |
| Portugal | POPRES | SE | 3.2% ± 0.3% | 2.5% ± 0.3% | 2.8% ± 0.2% |
| Sardinian | HGDP-CEPH | SE | 3.0% ± 0.4% | 3.0% ± 0.4% | 3.4% ± 0.3% |
| Southern-Italy | POPRES | SE | 2.8% ± 0.3% | 2.8% ± 0.3% | 3.2% ± 0.3% |
| Northern-Italy | POPRES | SE | 1.2% ± 0.3% | 1.2% ± 0.3% | 1.3% ± 0.3% |
| Swiss-French | POPRES | I | 0.5% ± 0.2% | 0.5% ± 0.2% | 0.5% ± 0.2% |
| Ashkenazi Jews | IBD | n/a | 3.3% ± 0.3% | 2.9% ± 0.3% | 2.9% ± 0.3% |
| Ashkenazi Jews | Jewish HapMap | n/a | 3.2% ± 0.4% | 2.9% ± 0.5% | 3.0% ± 0.4% |
| Syrian | Jewish HapMap | n/a | 3.9% ± 0.5% | 4.0% ± 0.5% | 4.6% ± 0.4% |
| Iranian | Jewish HapMap | n/a | 2.6% ± 0.6% | 2.7% ± 0.7% | 2.9% ± 0.5% |
| Iraqi | Jewish HapMap | n/a | 3.8% ± 0.6% | 3.9% ± 0.6% | 4.5% ± 0.4% |
| Sephardic Greek Jews | Jewish HapMap | n/a | 4.9% ± 0.4% | 4.9% ± 0.4% | 5.3% ± 0.4% |
| Sephardic Turkey Jews | Jewish HapMap | n/a | 4.6% ± 0.4% | 4.6% ± 0.4% | 5.5% ± 0.4% |
| Italian Jews | Jewish HapMap | n/a | 5.0% ± 0.5% | 5.0% ± 0.5% | 5.2% ± 0.4% |

Note: Estimates of proportions of mixture for all West Eurasians that give statistically signal evidence of mixture in Table 1. Regions are abbreviated as: I – Northwest Europe, SE – Southern Europe and L – Levant. Mixture proportions are based on *f_4_ Ancestry Estimation* method using the phylogenetic trees specified in columns 4, 5 and 6.
