# Supplementary material for: The History of African Gene Flow into Southern Europeans, Levantines, and Jews
Source: PLoS Genet. 2011 Apr 21;7(4):e1001373. doi: 10.1371/journal.pgen.1001373 (PMC3080861; doi:10.1371/journal.pgen.1001373)
Supplement: Table S7 — ROLLOFF Simulations: Effect of variations in bin sizes and genetic map. (0.04 MB DOC) [file pgen.1001373.s020.doc]

***Table S7.*** ROLLOFF Simulations: Effect of variations in bin sizes and genetic map

| **Category** |  | **Simulation at time depth (λ) = 10 generations** | **Simulation at time depth (λ) = 100 generations** |
| --- | --- | --- | --- |
| **Genetic Map** | 0.00001 | 11 ± 1 | 113 ± 5 |
| **Rate parameter (Φ)** | 0.0001 | 11 ± 1 | 113 ± 5 |
|  | 0.001 | 11 ± 1 | 114 ± 5 |
|  | 0.1 | 11 ± 1 | 113 ± 5 |
|  | 10 | 11 ± 1 | 113 ± 5 |
| **Bin Size (cM)** | 0.01 | 11 ± 1 | 114 ± 6 |
|  | 0.025 | 11 ± 1 | 114 ± 5 |
|  | 0.1 | 11 ± 1 | 113 ± 5 |
|  | 0.4 | 11 ± 1 | 115 ± 6 |
|  | 1 | 13 ± 1 | 145 ± 4 |

Note: We simulated 10 individuals using YRI and CEU as the ancestral populations where we set the mixture proportion to be θ = 20% and the time since mixture to be λ= 10 or 100 generations. We then performed *ROLLOFF* analysis with an independent dataset of European Americans and Nigerian Yorubans as reference population. To test the effect of inaccuracies in the genetic map, we systematically change the genetic map by modeling the change based on the convolution property of a gamma distribution with rate parameter φ. A low value of φ implies significant changes to the map and a high value allows for fine scale changes. To test the effect of the bin size, we vary the bin size within the range of 0.01 - 1cM and test the effect on the estimated dates.
