# Supplementary material for: The History of African Gene Flow into Southern Europeans, Levantines, and Jews
Source: PLoS Genet. 2011 Apr 21;7(4):e1001373. doi: 10.1371/journal.pgen.1001373 (PMC3080861; doi:10.1371/journal.pgen.1001373)
Supplement: Table S8 — ROLLOFF Simulations: Effect of inaccurate ancestral populations. (0.04 MB DOC) [file pgen.1001373.s021.doc]

***Table S8***. ROLLOFF simulations: Effect of inaccurate ancestral populations

| **Reference Populations** | **F_ST_with ancestral pops. CEU and YRI** | **Estimated date for time depth λ =10 generations** | **Estimated date for time depth λ = 100 generations** |
| --- | --- | --- | --- |
| European American | 0.00 | 11 ± 1 | 113 ± 5 |
| Yoruba | 0.00 |  |  |
|  |  |  |  |
| Basque | 0.01 | 11 ± 1 | 125 ± 8 |
| Mandenka | 0.01 |  |  |
|  |  |  |  |
| Druze | 0.02 | 11 ± 1 | 124 ± 12 |
| Yoruba | 0.00 |  |  |
|  |  |  |  |
| Druze | 0.02 | 11 ± 1 | 119 ± 12 |
| Kenyan Bantu | 0.01 |  |  |
|  |  |  |  |
| Gujarati | 0.03 | 11 ± 1 | 112 ± 6 |
| Maasai | 0.03 |  |  |
|  |  |  |  |

Note: We simulated 10 individuals using YRI and CEU as the ancestral populations where we set the mixture proportion to be θ = 20% and the time since mixture to be λ= 10 or 100 generations. We then performed *ROLLOFF* analysis with the reference populations shown in column 1. Average allele frequency difference (F_ST_) between the true ancestral population (CEU and YRI) and the reference population used for *ROLLOFF* analysis is shown in column 2.
