# Supplementary material for: The History of African Gene Flow into Southern Europeans, Levantines, and Jews
Source: PLoS Genet. 2011 Apr 21;7(4):e1001373. doi: 10.1371/journal.pgen.1001373 (PMC3080861; doi:10.1371/journal.pgen.1001373)
Supplement: Table S9 — ROLLOFF Simulations: Effect of inaccurate ancestral populations in the case of low mixture proportions and old mixture dates. (0.07 MB DOC) [file pgen.1001373.s022.doc]

***Table S9.*** ROLLOFF simulations: Effect of inaccurate ancestral populations in the case of low mixture proportions and old mixture dates.

| **Reference Populations used**  **in *ROLLOFF*** | **F_ST_ with true ancestral populations** | **Simulated date given a truth of λ=55 generations (bias in parentheses)** | | | **Simulated date given a truth of λ=89 generations (bias in parentheses)** | | |
| --- | --- | --- | --- | --- | --- | --- | --- |
|  |  | **θ= 1%** | **θ= 2%** | **θ= 3%** | **θ= 3%** | **θ= 4%** | **θ= 5%** |
| **European American (*n*=100)** | 0.00 | 68 (24%) | 62 (13%) | 59 (8%) | 98 (10%) | 98 (10%) | 95 (6%) |
| **Yoruba (*n*=100)** | 0.00 |  |  |  |  |  |  |
|  |  |  |  |  |  |  |  |
| **Basque (*n*=24)** | 0.01 | 71 (29%) | 63 (15%) | 60 (10%) | 101 (13%) | 100 (15%) | 95 (7%) |
| **Mandenka (*n*=22)** | 0.01 |  |  |  |  |  |  |
|  |  |  |  |  |  |  |  |
| **Druze (*n*=42)** | 0.02 | 74 (35%) | 63 (14%) | 61 (10%) | 102 (15%) | 100 (12%) | 96 (8%) |
| **Yoruba (*n*=21)** | 0.00 |  |  |  |  |  |  |
|  |  |  |  |  |  |  |  |
| **Druze (*n*=42)** | 0.02 | 75 (36%) | 63 (14%) | 61 (11%) | 103 (16%) | 100 (13%) | 97 (9%) |
| **Kenyan Bantu (*n*=11)** | 0.01 |  |  |  |  |  |  |
|  |  |  |  |  |  |  |  |
| **Gujarati (*n*=88)** | 0.03 | 75 (36%) | 66 (20%) | 61 (12%) | 103 (16%) | 102 (15%) | 97 (9%) |
| **Maasai (*n*=143)** | 0.03 |  |  |  |  |  |  |
|  |  |  |  |  |  |  |  |

Note: We simulated 20 individuals with CEU and YRI as the ancestral populations where we set the mixture proportion (θ), time since mixture (λ) and the reference populations used for the *ROLLOFF* analysis as shown above. We repeated each simulation 100 times and estimated the average and bias for each run. The values shown in the rows are the average for the 100 simulations and bias, defined as (average-truth)/(truth).
