# Supplementary material for: The History of African Gene Flow into Southern Europeans, Levantines, and Jews
Source: PLoS Genet. 2011 Apr 21;7(4):e1001373. doi: 10.1371/journal.pgen.1001373 (PMC3080861; doi:10.1371/journal.pgen.1001373)
Supplement: Table S10 — ROLLOFF Simulations: Effect of number of admixed samples. (0.03 MB DOC) [file pgen.1001373.s023.doc]

***Table S10.*** ROLLOFF simulations: Effect of number of admixed samples

| **# of admixed samples** | **Average estimated date (bias in simulations)** |
| --- | --- |
| *n*=10 | 66 (22%) |
| *n*=20 | 56 (4%) |
| *n*=30 | 56 (4%) |
| *n*=40 | 57 (6%) |
| *n*=50 | 55 (2%) |
| *n*=80 | 54 (0%) |
| *n*=100 | 54 (0%) |

Note: We simulated *n* individuals using European Americans and Nigerians as the ancestral populations where we set the mixture proportion to be θ=2% and the time since mixture to be λ= 54 generations, and then performed *ROLLOFF* analysis with HapMap3 CEU and YRI as the reference populations. We repeated each simulation 100 times and estimated the average and bias. The values shown in the cells are the average over the 100 simulations and the bias, defined as (average-truth)/(truth).
