# Supplementary material for: The History of African Gene Flow into Southern Europeans, Levantines, and Jews
Source: PLoS Genet. 2011 Apr 21;7(4):e1001373. doi: 10.1371/journal.pgen.1001373 (PMC3080861; doi:10.1371/journal.pgen.1001373)
Supplement: Table S11 — ROLLOFF Simulations: Effect of mixture proportions. (0.03 MB DOC) [file pgen.1001373.s024.doc]

***Table S11.*** ROLLOFF simulations: Effect of mixture proportions

| **Mixture proportion** | **Average Estimated date (bias in simulations)** |
| --- | --- |
| θ=1% | 62 (15%) |
| θ=2% | 58 (7%) |
| θ=3% | 57 (6%) |
| θ=5% | 55 (2%) |
| θ=10% | 54 (0%) |
| θ=20% | 53 (2%) |
| θ=30% | 53 (2%) |
| θ=50% | 53 (2%) |

Note: We simulated 50 individuals using YRI and CEU as the ancestral populations where we set the mixture proportion to be θ (shown in the table) and the time since mixture to be λ= 54 generations. We then performed *ROLLOFF* analysis with an independent dataset of 100 European Americans and 100 Nigerian Yorubans as reference population. We repeated each simulation 100 times and estimated the average and bias. The bias is defined as (average-truth)/(truth).
