# Supplementary material for: The History of African Gene Flow into Southern Europeans, Levantines, and Jews
Source: PLoS Genet. 2011 Apr 21;7(4):e1001373. doi: 10.1371/journal.pgen.1001373 (PMC3080861; doi:10.1371/journal.pgen.1001373)
Supplement: Table S12 — ROLLOFF Simulations: Continuous admixture scenarios. (0.04 MB DOC) [file pgen.1001373.s025.doc]

**Table S13. *ROLLOFF Simulations: Continuous admixture scenarios***

|  | **Δ =1** | **Δ =5** | **Δ =10** | **Δ =20** | **Δ =30** | **Δ =100** |
| --- | --- | --- | --- | --- | --- | --- |
| ***a* =1** | 1 ± 0 | 4 ± 0 | 4 ± 1 | 6 ± 1 | 11 ± 2 | 30 ± 6 |
| ***a* =25** | 26 ± 2 | 25 ± 2 | 30 ± 3 | 33 ± 2 | 41 ± 2 | 63 ± 6 |
| ***a* =50** | 55 ± 3 | 56 ± 3 | 52 ± 3 | 59 ± 5 | 60 ± 5 | 77 ± 6 |
| ***a* =100** | 100 ± 6 | 117 ± 7 | 109 ± 7 | 102 ± 8 | 118 ± 5 | 142 ± 13 |
| ***a* =200** | 204 ± 18 | 188 ± 19 | 202 ± 17 | 253 ± 27 | 198 ± 19 | 268 ± 25 |

Note: We performed 30 simulations with CEU and YRI as the ancestral populations using the simulation method described in Note S3f. In each simulation, we varied the length of the interval *I (Δ = b-a)* during which YRI lineages migrate to the CEU population (creating an admixed population). Following the YRI mixture, there are ‘*a’* generations of random mixture between the admixed individuals. This can roughly be thought of as simulating genetic drift, since admixture. We performed *ROLLOFF* analysis using a non-overlapping dataset of 1,107 European American and 737 Nigerian Yoruba individuals as reference samples.
