# Supplementary material for: The History of African Gene Flow into Southern Europeans, Levantines, and Jews
Source: PLoS Genet. 2011 Apr 21;7(4):e1001373. doi: 10.1371/journal.pgen.1001373 (PMC3080861; doi:10.1371/journal.pgen.1001373)
Supplement: Table S13 — ROLLOFF analysis of West Eurasians: bias in the estimated date for empirically estimated parameters. (0.07 MB DOC) [file pgen.1001373.s026.doc]

***Table S12.*** ROLLOFF analysis of West Eurasians: bias in the estimated date for empirically estimated parameters

| **Population (X)** | **Dataset** | **Samples** | **West African ancestry proportion ± standard error** | **Estimated date of admixture (generations) +/- standard error** | **Simulation Results**  **Average (bias in simulations)** |
| --- | --- | --- | --- | --- | --- |
|
| African American | HapMap3 | 49 | 79.4% ± 0.3% | 6 ± 1 | 6 (0%) |
| Palestinian | HGDP-CEPH | 43 | 9.3% ± 0.4% | 34 ± 2 | 35 (3%) |
| Bedouin-g1 | HGDP-CEPH | 15 | 14.5% ± 0.4% | 34 ± 3 | 36 (6%) |
| Bedouin-g2 | HGDP-CEPH | 30 | 10.1% ± 0.4% | 33 ± 2 | 35 (6%) |
| Druze | HGDP-CEPH | 41 | 4.4% ± 0.4% | 54 ± 7 | 64 (19%) |
| Spain | POPRES | 137 | 2.4% ± 0.3% | 55 ± 3 | 55 (0%) |
| Portugal | POPRES | 134 | 3.2% ± 0.3% | 45 ± 5 | 45 (0%) |
| Sardinian | HGDP-CEPH | 27 | 2.9% ± 0.5% | 96 ± 28 | 121 (26%) |
| Southern-Italy | POPRES | 121 | 2.7% ± 0.3% | 62 ± 6 | 62 (0%) |
| Northern-Italy | POPRES | 90 | 1.1% ± 0.3% | 154 ± 27 | 128 (-17%) |
| Swiss-French | POPRES | 759 | 0.5% ± 0.2% | 71 ± 6 | n/a |
| Ashkenazi Jews | IBD | 323 | 2.8% ± 0.3% | 91 ± 11 | n/a |
| Ashkenazi Jews | Jewish HapMap | 34 | 3.2% ± 0.4% | 76 ± 13 | 99 (31%) |
| Syrian Jews | Jewish HapMap | 25 | 3.9% ± 0.5% | 99 ± 23 | 126 (27%) |
| Iranian Jews | Jewish HapMap | 24 | 2.6% ± 0.6% | 129 ± 34 | 188 (46%) |
| Iraqi Jews | Jewish HapMap | 36 | 3.8% ± 0.5% | 153 ± 22 | 191 (25%) |
| Sephardic Greek Jews | Jewish HapMap | 39 | 4.8% ± 0.4% | 82 ± 8 | 102 (24%) |
| Sephardic Turkey Jews | Jewish HapMap | 27 | 4.5% ± 0.4% | 89 ± 11 | 105 (18%) |
| Italian Jews | Jewish HapMap | 27 | 4.9% ± 0.5% | 88 ± 19 | 103 (17%) |

Note: We simulated individuals of mixed European and African ancestry where we set the sample size, mixture proportion (θ) and time since mixture (λ) to match the parameters estimated for West Eurasians. We then performed *ROLLOFF* analysis using HapMap3 Italian Toscanis (TSI) and Kenyan Luhya (LWK) as reference populations (as data for the true ancestral populations might not always be available for real samples). We repeated each simulation 100 times and estimated the average and bias. Average = mean of the estimated date for 100 simulations and Bias = (average-truth)/(truth). We were not able to perform simulations for Swiss-French and IBD Ashkenazi Jews because we did not have a sufficiently large pool of ancestral haplotypes to accommodate the large sample sizes in these groups.
