# Supplementary material for: The History of African Gene Flow into Southern Europeans, Levantines, and Jews
Source: PLoS Genet. 2011 Apr 21;7(4):e1001373. doi: 10.1371/journal.pgen.1001373 (PMC3080861; doi:10.1371/journal.pgen.1001373)
Supplement: Table S14 — 4 Population Test to distinguish between East & West African ancestry. (0.05 MB DOC) [file pgen.1001373.s027.doc]

***Table S14.*** 4 Population Test to distinguish between East & West African ancestry

| **Pop X** | **Dataset** | **Region** | **(P_CEU_-P_X_) (P_YRI_-P_LWK_)** |
| --- | --- | --- | --- |
| African Americans | HapMap3 | n/a | **-46.8** |
| Mozabite | HGDP-CEPH | n/a | **-24.5** |
| Palestinian | HGDP-CEPH | L | **-5.1** |
| Bedouin-g1 | HGDP-CEPH | L | **-7.2** |
| Bedouin-g2 | HGDP-CEPH | L | -1.9 |
| Druze | HGDP-CEPH | L | 0.4 |
| Spain | POPRES | SE | -2.5 |
| Portugal | POPRES | SE | -2.9 |
| Northern Italy | POPRES | SE | 0.9 |
| Southern Italy | POPRES | SE | 1.3 |
| Sardinian | HGDP-CEPH | SE | 1.9 |
| Swiss-French | POPRES | I | 1.0 |
| Ashkenazi Jews | IBD | n/a | 0.0 |
| Ashkenazi Jews | Jewish HapMap | n/a | 0.8 |
| Iranian Jews | Jewish HapMap | n/a | 1.0 |
| Iraqi Jews | Jewish HapMap | n/a | 2.1 |
| Italian Jews | Jewish HapMap | n/a | 1.1 |
| Sephardic Greek Jews | Jewish HapMap | n/a | 1.0 |
| Sephardic Turkey Jews | Jewish HapMap | n/a | 0.6 |
| Syrian Jews | Jewish HapMap | n/a | 0.9 |

Note: We analyzed data from all West Eurasian populations that showed evidence of African ancestry in Table1. Regions are abbreviated as: I – Northwest Europe, SE – Southern Europe and L – Levant. For the *4 Population Test,* we report only results for the tree shown in the table. Results for all alternate topologies show even higher violations of the tree (|Z| >> 15). Scores that are significant are highlighted in bold.
