# Supplementary material for: The History of African Gene Flow into Southern Europeans, Levantines, and Jews
Source: PLoS Genet. 2011 Apr 21;7(4):e1001373. doi: 10.1371/journal.pgen.1001373 (PMC3080861; doi:10.1371/journal.pgen.1001373)
Supplement: Table S15 — Estimated mixture proportion and date using East Africans as reference. (0.05 MB DOC) [file pgen.1001373.s028.doc]

**Table S15. *Estimated mixture proportion and date using East Africans as reference population***

| **Population (X)** | **Dataset** | **Region** | **Samples** | **East African ancestry proportion ± standard error** | **Estimated date of admixture (generations) +/- standard error** |
| --- | --- | --- | --- | --- | --- |
|
| African Americans | HapMap3 | n/a | 49 | 81.1% ± 0.3% | 6 ± 1 |
| Palestinian | HGDP-CEPH | L | 43 | 10.6% ± 0.3% | 33 ± 2 |
| Bedouin-g1 | HGDP-CEPH | L | 15 | 15.5% ± 0.4% | 34 ± 3 |
| Bedouin-g2 | HGDP-CEPH | L | 30 | 11.3% ± 0.4% | 34 ± 3 |
| Druze | HGDP-CEPH | L | 41 | 5.4% ± 0.3% | 56 ± 7 |
| Spain | POPRES | SE | 137 | 2.9% ± 0.2% | 57 ± 3 |
| Portugal | POPRES | SE | 134 | 3.6% ± 0.2% | 46 ± 5 |
| Sardinian | HGDP-CEPH | SE | 27 | 3.5% ± 0.3% | 93 ± 30 |
| Southern-Italy | POPRES | SE | 121 | 3.3% ± 0.3% | 62 ± 6 |
| Northern-Italy | POPRES | SE | 90 | 1.3% ± 0.3% | 158 ± 31 |
| Swiss-French | POPRES | I | 759 | 0.5% ± 0.2% | 74 ± 6 |
| Ashkenazi Jews | IBD | n/a | 323 | 3.0% ± 0.3% | 89 ± 12 |
| Ashkenazi Jews | Jewish HapMap | n/a | 34 | 3.3% ± 0.5% | 77 ± 14 |
| Syrian Jews | Jewish HapMap | n/a | 25 | 4.0% ± 0.5% | 103 ± 23 |
| Iranian Jews | Jewish HapMap | n/a | 24 | 2.7% ± 0.7% | 114 ± 30 |
| Iraqi Jews | Jewish HapMap | n/a | 36 | 3.9% ± 0.6% | 149 ± 23 |
| Sephardic Greek Jews | Jewish HapMap | n/a | 39 | 4.9% ± 0.4% | 80 ± 7 |
| Sephardic Turkey Jews | Jewish HapMap | n/a | 27 | 4.6% ± 0.4% | 91 ± 11 |
| Italian Jews | Jewish HapMap | n/a | 27 | 5.0% ± 0.5% | 89 ± 18 |

Note: Regions are abbreviated as: I – Northwest Europe, SE – Southern Europe and L – Levant. Mixture proportion estimates are based on *f_4_ Ancestry Estimation* using San, LWK, CEU and Papuan as the reference populations. The *ROLLOFF* estimated date of mixture uses CEU and LWK as the reference populations.
