# Supplementary material for: The History of African Gene Flow into Southern Europeans, Levantines, and Jews
Source: PLoS Genet. 2011 Apr 21;7(4):e1001373. doi: 10.1371/journal.pgen.1001373 (PMC3080861; doi:10.1371/journal.pgen.1001373)
Supplement: Table S16 — ROLLOFF Analysis for different jackknife block sizes: example Spain. (0.03 MB DOC) [file pgen.1001373.s029.doc]

***Table S16.*** ROLLOFF Analysis for different jackknife block sizes: example Spain

| **Jackknife Block size** | **Estimated date ± standard error** |
| --- | --- |
| 1 chromosome | 55 ± 3 |
| 5cM | 55 ± 3 |
| 10cM | 55 ± 3 |
| 20cM | 55 ± 3 |

NOTE: The *ROLLOFF* estimated date of mixture uses CEU and YRI as the reference populations.
