# Supplementary material for: The History of African Gene Flow into Southern Europeans, Levantines, and Jews
Source: PLoS Genet. 2011 Apr 21;7(4):e1001373. doi: 10.1371/journal.pgen.1001373 (PMC3080861; doi:10.1371/journal.pgen.1001373)
Supplement: Text S1 — PCA-based search for outliers and sub-structure. (0.03 MB DOC) [file pgen.1001373.s030.doc]

**Text S1. PCA-based search for outliers and sub-structure**

We performed Principal Component analysis (PCA) analysis with the unmixed ancestral West Eurasians (CEU) and ancestral Africans (YRI) and each West Eurasian population (*X*) to study the relationship of individuals within population *X* to sub-Saharan Africans. A plot of the first and second PCs separates the YRI from CEU and West Eurasians fall between these two populations, depending on their genetic proximity to Africans (Figure S1). We observe that most populations are homogenous in their relationship to sub-Saharan Africans, with a few outliers. In order to obtain sample estimates that are more representative of the bulk of the populations, a total of 140 outliers were removed based on PCA (Table S2). Here are a few notes related to the PCA.

1. We removed all samples from the POPRES Greece population from our analysis, as PCA showed that the 8 Greek individuals formed 3 separate clusters, with evidently different proportions of African relatedness. It was not clear which cluster was most characteristic of the larger Greek population, and hence we excluded the population altogether.

2. Combined PCA of CEU, YRI and all Italian populations shows that there are three significant clusters among the Italian populations: Sardinian, Northern-Italy and Southern-Italy. The “Southern-Italy” group mainly consists of individuals from the POPRES Italy population that appear to be sampled from the south of Italy based on the clustering seen in PCA. The “Northern-Italians” group contains data from POPRES Italy and Swiss-Italians and HGDP-CEPH Bergamo and Tuscany and “Sardinians” include individuals from HGDP-CEPH Sardinia and POPRES Italy population, which appear to be closely related to this group (Figure S1C). Individuals that did not fall into these three main clusters were excluded from all further analysis.

3. The HGDP-CEPH Bedouin population was divided into 2 groups – Bedouin-g1 and Bedouin-g2. The Bedouin-g1 population is more similar to the HGDP-CEPH Palestinian population (Figure S1D) compared to the other Bedouin group (Bedouin-g2).

4. PCA analysis of YRI, CEU and 389 IBD Ashkenazi Jews shows that a large number of Ashkenazi Jews cluster together forming a main groups of ~320 individuals. The rest of the individuals are very heterogeneous and we hypothesize that they have experienced recent admixture with non-Ashkenazi groups (Figure S1E). We excluded 69 individuals that do not fall in the main cluster.

5. In the case of the Jewish samples from the Jewish HapMap project, we applied a slightly different algorithm to search for outliers. In addition to performing PCA for each Jewish population *X* and CEU and YRI, we performed a combined PCA of all Jewish populations. This was done to confirm that the self-reported ancestry of the Jewish individuals correlates with the ancestry based on genetic data. In some cases, we found that there was significant heterogeneity in the population (for example: Italian Jews). Hence, we removed all individuals that did not cluster with the bulk of individuals of that group. We also identified some individuals that clustered with groups other than their own, and these individuals were also excluded from further analysis (Figure S1E).

Comparison of results before and after curation shows that the data curation does not affect the qualitative inferences (Table S3).
