# Supplementary material for: The History of African Gene Flow into Southern Europeans, Levantines, and Jews
Source: PLoS Genet. 2011 Apr 21;7(4):e1001373. doi: 10.1371/journal.pgen.1001373 (PMC3080861; doi:10.1371/journal.pgen.1001373)
Supplement: Text S2 — Robustness of inferences to the choice of ancestral populations. (0.04 MB DOC) [file pgen.1001373.s031.doc]

**Text S2. Robustness of inferences to the choice of ancestral populations**

To test whether our inferences are sensitively dependent on the ancestral populations used as reference populations, we performed all the tests substituting YRI and CEU with other related populations.

(a) We computed principal components (PCs) with EIGENSOFT [1] using the Kenyan Bantu and Adygei (a population from the Northern Caucasus) and projected various West Eurasian and African populations onto the PCs. A plot of the first and second PCs shows that West Eurasian populations form a gradient of relatedness to Kenyan Bantu, with other African populations having frequencies that are most correlated to Kenyan Bantu and the Northern European populations having frequencies that are least correlated. We observe that Southern European, Jewish and Levantine populations are close to Adygei but are slightly shifted towards Kenyan Bantu compared to Northern European populations, just as in Figure 1 (Figure S2).

(b) To assess the robustness of our inferences from Table 1*,* we repeated the *4 Population Test* with alternate tree topologies where we replaced the ancestral West Eurasian and ancestral sub-Saharan African populations. Alternate topologies tested were as follows:

(i) ((YRI, Papuan), (Adygei, *X*)) (*X* is a range of West Eurasian populations tested)

(ii) ((Mandenka, Papuan), (CEU, *X*))

(iii) ((Kenyan Bantu, Papuan), (CEU, *X*))

In all three cases, we observed significant violations of the expected tree topology for Southern European, Jewish groups and Levantine populations (Table S4). The Northern European populations showed no evidence of admixture (Table S4).

(c) To demonstrate that our estimates of African mixture proportions are not sensitive to the sub-Saharan African group we compared to, we use *f_4_ Ancestry Estimation* [2] with the alternate phylogenetic tree (San,(*Y*,(Papuan,(CEU,*X*)))), where *X* = range of West Eurasian populations that show violations of the *4 Population Test* [2] and *Y*= Mandenka or Kenyan Bantu. We also replaced the outgroup Papuan with the HapMap3 Han Chinese (CHB). Our analyses show that the tests are robust to the ancestral populations or the outgroup chosen, as the results are qualitatively similar (within 2 standard deviations) (Table S5).

***References***

1. Patterson N, Price A, Reich D (2006) Population structure and eigenanalysis. PLoS Genet 2: e190.

2. Reich D, Thangaraj K, Patterson N, Price A, Singh L (2009) Reconstructing Indian population history. Nature 461: 489-494.
