# Supplementary material for: The History of African Gene Flow into Southern Europeans, Levantines, and Jews
Source: PLoS Genet. 2011 Apr 21;7(4):e1001373. doi: 10.1371/journal.pgen.1001373 (PMC3080861; doi:10.1371/journal.pgen.1001373)
Supplement: Text S3 — Effect of SNP ascertainment bias on results of 3 Population Test. (0.03 MB DOC) [file pgen.1001373.s032.doc]

**Text S3. Effect of SNP ascertainment bias on results of *3 Population Test***

To investigate if SNP ascertainment bias can affect the results of the *3 Population Test*, we performed coalescent simulation using Hudson’s *ms* [1] to generate data for two ancestral populations, Population *A* (Pop *A*) and Population *B* (Pop *B*). For the simulation, we use a two-population demography similar to one used in reference [2] where the effective population size of Pop A is N_0_ = 10,000 and effective population size of Pop B varies from 0.25N_0_ to 0.85N_0_ such that the frequency differentiation F_ST_(A,B)=0.15 and the divergence time varies from 45,000 - 100,000 years. These simulated populations can be roughly considered as Africans and Europeans. Details of the demographic model are presented in Figure S4. In order to generate SNPs affected by ascertainment bias, we select two chromosomes and examine the alleles. If the two alleles are different, we record the data for the SNP. There are three ascertainment schemes:

(1) One chromosome is selected from each of Pop *A* and Pop *B*

(2) Both the chromosomes are selected from Pop *A*

(3) Both the chromosomes are selected from Pop *B*

We construct the genomes of individuals of Pop *C* who have mixed Pop *A* and Pop *B* ancestry by using the simulator described in the Materials and Methods section. We choose a 20%/80% mixing proportion and set the time since mixture to be 10 generations. We then perform the *3 Population Test* [3] (C; A, B). We observe that regardless of the ascertainment scheme or the demographic model chosen, there is clear evidence of mixture in Pop *C* (Table S6) and our results are not affected by ascertainment bias.

***References***

1. Hudson R (2002) Generating samples under a Wright-Fisher neutral model of genetic variation. Bioinformatics 18: 337.

2. Sun J, Mullikin J, Patterson N, Reich D (2009) Microsatellites are molecular clocks that support accurate inferences about history. Molecular biology and evolution 26: 1017.

3. Reich D, Thangaraj K, Patterson N, Price A, Singh L (2009) Reconstructing Indian population history. Nature 461: 489-494.
