# Supplementary material for: The History of African Gene Flow into Southern Europeans, Levantines, and Jews
Source: PLoS Genet. 2011 Apr 21;7(4):e1001373. doi: 10.1371/journal.pgen.1001373 (PMC3080861; doi:10.1371/journal.pgen.1001373)
Supplement: Text S4 — Robustness of the ROLLOFF method for estimating mixture dates. (0.09 MB DOC) [file pgen.1001373.s033.doc]

**Text S4. Robustness of the *ROLLOFF* method for estimating mixture dates**

***(a) ROLLOFF simulations for a scenario similar to African Americans***

We simulated genomes of 10 individuals of mixed European and African ancestry using the simulation framework described in the *Materials and Methods*. We set the time since mixture (λ) at 6 generations and the European ancestry proportion (θ) was sampled from a beta distribution with mean 20% and standard deviation 10%. These parameters were chosen to be within the ranges of values that are typical for African Americans[1]. *ROLLOFF* analysis was performed using a non-overlapping dataset of 1,107 European American and 737 Yoruba Nigerian individuals as reference populations. The analysis was restricted to 339,171 SNPs and the fine scale recombination map of Myers et al.[2] was used for specifying the genetic distance.

A plot of the admixture LD shows an approximately exponential decay of LD with genetic distance. The half-life suggests a mixture date of 6 ± 1 generation (Figure S7). The standard error was calculated using the *Weighted Block Jackknife* where we remove one chromosome in each run and measure the variance in the date to assess the stability of the inference (see Materials and Methods).

***(b) ROLLOFF analysis to test the effect of errors in the genetic map***

To test the dependence of the *ROLLOFF* estimates on the precision of the genetic map, we systematically change the genetic map by modeling the change based on the convolution property of a gamma distribution with rate parameter φ. A low value of φ implies significant changes to the map and a high value allows for fine scale changes.

Suppose we model the genetic distance (*d*_i_) between any pair of SNPs (*s*_1_ and *s*_2_) as a gamma distribution with two parameters such that *k* is the shape parameter and μ is scale parameter that is the reciprocal of the rate parameter (μ = 1/φ), then the probability distribution of distance *d*_i_ is given by


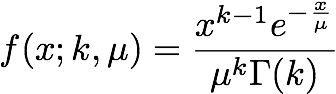
 for x, μ ≥ 0 (1)

Just by changing the rate parameter (φ), one can make fine scale changes to the map without changing the overall mean of the distribution. Using this method, we created different genetic maps by setting φ to range of values varying from 0.001 to 10 (multiples of 10). We simulated 10 individuals of mixed European and African ancestry that had 20% European and 80% African ancestry with the mixture occurring 10 and 100 generations ago. We used a non-overlapping dataset of European Americans and Nigerians as the reference populations and the inaccurate genetic map for modeling in *ROLLOFF*.

We observe that *ROLLOFF* is robust to errors in the genetic map and the estimated date falls within two standard deviations of the truth for the different maps (Table S7).

***(c) ROLLOFF analysis to test the effect of different bin sizes***

To be computationally efficient, we divide the genome into bins separated by distance *d* and estimate the correlation between all possible pairs of SNPs in each bin. To test if the bin size has an effect on the results of *ROLLOFF*, we performed simulations with variable bin sizes and estimated the date of admixture. We simulated individuals of mixed European and African ancestry that had 20% European and 80% African ancestry with the mixture occurring 10 generations ago (10 individuals) and mixture occurring 100 generations ago (10 individuals). We performed four separate *ROLLOFF* analyses using a non-overlapping dataset of European American and Nigerians as reference populations with the bin size within the range of 0.001 - 1cM (we picked 5 values in this range).

For mixture occurring 10 generations ago, we observe that all the estimated dates fall within two standard deviations of the true (simulated) time depth. However, bin sizes of 0.5cM and greater contain very few points within each bin and thus the results should be considered with caution. We repeated the analysis for mixture occurring 100 generations ago and observed similar results. Based on these results, we conclude that the optimal bin size should be within the range 0.001 – 0.5 cM. For all our analyses, we use a bin size of 0.1cM (Table S7).

***(d) Simulation searching for bias in ROLLOFF***

In many cases, genetic data for the true ancestral populations for a particular admixture event is not available either because the populations involved in the mixture are no longer extant (as the modern day population is very diverged from the ancestral population) or they have not been genotyped. In such cases, to the best that we can do is to use present-day populations that most closely resemble the true ancestral populations. To test the effect of using an inaccurate reference population on the estimation of a date by *ROLLOFF*, we constructed 10 diploid genomes of individuals of mixed ancestry with 20% European and 80% African ancestry that were simulated using CEU and YRI individuals but modeled using other reference populations. We performed these simulations for mixture occurring 10 generations ago as well as 100 generations ago. We performed *ROLLOFF* analysis with 4 sets of reference populations. These were:

(i) French Basque and Senegal Mandenka

(ii) Druze and Kenyan Bantu

(iii) HapMap3 Gujarati (GIH) and Kenyan Maasai (MKK)

(iv) Druze and Yoruba

In all cases, we observe that *ROLLOFF* can accurately estimate the date of mixture for the recent admixture date of 10 generations, even though inaccurate reference populations are used. Even at more ancient dates, the results are within two standard deviations of the true simulated date (Table S8). This shows that *ROLLOFF* is robust to somewhat inaccurate parental populations and should be useful even in cases where it is difficult to obtain genetic data from the true ancestral populations. In particular, it suggests that our method is likely to give unbiased results for West Eurasians regardless of the sub-Saharan African ancestral population chosen.

To assess if inaccurate ancestral populations give unbiased results for a real admixed population, we applied *ROLLOFF* to data from African Americans (HapMap3 ASW), using Senegal Mandenka and Basque as reference population. We estimated that the date of admixture for ASW is 6 ± 1 generation, which is consistent with previously published reports [1,3] and with the date estimated if YRI and CEU (which are likely close to the true ancestral populations) are used as reference populations. The date remains unchanged even if Druze and YRI are used as reference populations.

In addition, we carried out simulations to test the performance of *ROLLOFF* in situations of very low mixture proportions and old mixture dates as seen in Southern Europeans and Jewish groups. We simulated data for 20 individuals of mixed YRI and CEU ancestry with the mixture proportion and date of mixture selected to match Southern Europeans (1-3% mixture proportion, 55 generations ago) and Jewish groups (3-5% mixture proportion, 89 generations ago). We then ran *ROLLOFF* with the same set of inaccurate reference populations as shown above. We repeated each simulation 100 times and then computed the average date and bias (here, *bias* is defined as (average-truth)/(truth)).

We observed that in the case of low mixture proportions and old mixture dates, there is an upward bias in the estimated dates (Table S9). This effect is attenuated as the number of admixed samples increases (Table S10) and as the mixture proportion increases (Table S11) but does not seem to be significantly affected by the ancestral populations used as reference in the *ROLLOFF* analysis (Table S9).

To test how much this effect is biasing our estimated dates for West Eurasians, we performed simulations to generate data for individuals of mixed European and African ancestry where we set the mixture proportion (θ), time since mixture (λ) and number of samples to match the parameters estimated for each West Eurasian group individually. We then performed *ROLLOFF* analysis using HapMap3 Italian Toscanis (TSI) and Kenyan Luhya (LWK) as reference populations. We repeated each simulation 100 times and computed the average and bias. The bias is in general very small except in the case where the mixture dates are old and sample sizes are small, which is the case for the Druze and most of the Jewish groups, where the bias is typically at least 20% (Table 2 and Table S12). We discuss this bias and report a bias correction in the main text.

***(e) ROLLOFF analysis for double admixture scenarios***

A potential pitfall in estimating dates of admixture is that the historical mixture event may not have occurred all at once, but instead may have taken place over multiple different times (pulse migration model) so that the pattern in the data in fact reflects a range of mixture times. To explore this, we ran *ROLLOFF* to infer the date of admixture on data simulated under a double admixture scenario (where there were two distinct events of gene flow between the populations). We simulated double admixture scenarios in which a 90%/10% admixture of CEU and YRI occurred at λ= 30, followed by a 60%/40% mixture of that admixed population and YRI at λ= 6. We also simulated data for a 50%/50% admixture of CEU and YRI that occurred at λ= 30, followed by a 50%/50% mixture of that admixed population and YRI at λ= 10 and 80%/20% admixture of CEU and YRI at λ= 40, followed by a 20%/80% mixture of that admixed population and YRI at λ= 20. For each simulation, genomes of 20 admixed individuals were constructed and *ROLLOFF* analyses were performed using a non-overlapping dataset of European Americans and Nigerians as reference populations.

Applying *ROLLOFF* to the simulated data, we observed that for case 1 where the mixture occurred at λ = 30 generations followed by λ= 6 generations, the date of admixture was estimated at 34 and 5 generations, when we fitted a sum of two exponentials and 6 generations when we fitted a single exponential distribution to the decay of the correlation coefficients. Similarly, *ROLLOFF* was able to estimate the date of admixture for the simulations for double admixture of 50%/50% mixture of CEU and YRI that occurred at λ= 30, followed by a 50%/50% mixture of that admixed population and YRI at λ= 10 accurately (35 and 9 generations when output was fitted with a sum of two exponentials and 11 generations when fitted with a single exponential) (Figure S8). However, for simulations of more ancient mixture dates of 80%/20% admixture of CEU and YRI that occurred at λ = 40, followed by a 20%/80% mixture of that admixed population and YRI at λ = 20, we could only reliably estimate the date for the more recent admixture event (24 and 2 generations when output was fitted with a sum of two exponentials and 22 generations when fitted with a single exponential). Standard errors were not computed for this analysis, as it is not clear how to apply the standard Jackknife theory for analysis with a sum of exponentials.

The dates for the recent admixture event were qualitatively similar to the true time depth, when the data was fitted with a single exponential or sum of two exponentials. A caveat is that we have only simulated a limited number of double mixture scenarios. In principle, further exploration of different values of (θ, λ) might identify situations in which we could estimate the date of the older mixture event using *ROLLOFF*.

***(f)*** ***Simulations of continuous admixture***

To model continuous gene flow, we simulated recurrent mixture over a specified number of discrete generations- varying between 1 to 100 generations (graduation mixture model). In each simulation, we generated data for individuals of mixed ancestry using two ancestral populations (CEU and YRI), where the gene flow occurred in an interval *I* = [*a*,*b*] where 0 ≤ *a* ≤ *b*. In each generation during *I*, we allow a proportion *m* (computed based on mixture proportion (θ)) of YRI lineages to migrate, yielding a total of 20% average African ancestry in the resulting admixed samples (Figure S9). At generation 1, we sample African haplotypes with probability (θ) and European haplotypes with probability (1-θ). We resample ancestry at each marker with probability 1-e^-g^, where *g* is the genetic distance between markers (in Morgans). Once the ancestry is sampled, a haplotype is copied from an individual of the chosen population (YRI or CEU) and copied to the genome of the admixed individual and the process is continued until the end of chromosome is reached. After the first generation, we allow there to be uni-directional migration from YRI into the admixed individuals. The pool of ancestral haplotypes is updated in each generation with haplotypes from the previous generation and this procedure is repeated until the end of the interval *I*. Next, if parameter *a* ≠ 0, then following YRI mixture, there are *a* generations of random mixture between the admixed individuals only. This can roughly be thought of as simulating genetic drift, since admixture. This procedure is repeated to create the genomes of 20 admixed individuals and pairs of haploid individuals are combined to construct 10 diploid admixed individuals.

In order to test the performance of *ROLLOFF,* we performed 30 simulations. In each simulation, we varied the values of the length of the interval *I = b-a* and the time since mixture *(a)*. We performed *ROLLOFF* analysis using a non-overlapping dataset of 1,107 European American and 737 Nigerian Yoruba individuals as reference samples. All analyses were restricted to 339,171 SNPs and the fine scale recombination map by Myers et al. [2] was used for mapping the genetic distance.

We applied *ROLLOFF* to the simulated data, fitting a single exponential decay in each case. We observe that when the interval *I* is very small, the *ROLLOFF* result correlates to the time since the last mixture event. However, as length of the interval *I* increases, the estimated dates reflect averages of mixture dates over a range of the interval (Table S13).

***(g) ROLLOFF analysis in cases of no mixture related to the reference populations***

We performed *ROLLOFF* analysis to estimate the date of admixture in the East Asian Uygur (HGDP-CEPH- Uygur) population who are known to have both West Eurasian and East Asian ancestries [4,5]. We used extremely inaccurate and unrelated populations— African Pygmies (HGDP-CEPH- Mbuti and Biaka Pygmies) and Nigerian YRI—as reference populations to test the performance of the *ROLLOFF* in cases when there is no admixture related to the reference populations. To contrast the situation of no mixture and to ensure that there are no technical issues with the dataset, we simulated 20 individuals of mixed Pygmy and Yoruba ancestry as positive control. These samples were simulated using HGDP-CEPH Biaka and Mbuti Pygmies and HGDP-CEPH Yoruba individuals as ancestral populations with 20% Pygmy ancestry and 80% Yoruba ancestry with the mixture occurring 10 generations (10 individuals) and 100 generations ago (10 individuals). We used a dataset containing 591,320 SNPs and used Pygmies and YRI as reference populations for *ROLLOFF*.

We observed clear evidence of admixture in the simulated individuals as we see an approximately exponential decay of LD with distance in the simulated individuals, with estimated dates of mixture as 10 and 90 generations. However, we observe that the correlation is almost zero in the Uygur population (Figure S11). This is consistent with expectation as these populations do not have Pygmy and YRI ancestry or ancestry from populations closely related to Pygmy or YRI.

**References**

1. Smith M, Patterson N, Lautenberger J, Truelove A, McDonald G, et al. (2004) A high-density admixture map for disease gene discovery in african americans. The American Journal of Human Genetics 74: 1001-1013.

2. Myers S, Bottolo L, Freeman C, McVean G, Donnelly P (2005) A fine-scale map of recombination rates and hotspots across the human genome. American Association for the Advancement of Science. pp. 321-324.

3. Price A, Tandon A, Patterson N, Barnes K, Rafaels N, et al. (2009) Sensitive Detection of Chromosomal Segments of Distinct Ancestry in Admixed Populations. PLoS Genetics 5.

4. Xu S, Jin L (2008) A genome-wide analysis of admixture in Uyghurs and a high-density admixture map for disease-gene discovery. The American Journal of Human Genetics 83: 322-336.

5. Li J, Absher D, Tang H, Southwick A, Casto A, et al. (2008) Worldwide human relationships inferred from genome-wide patterns of variation. Science 319: 1100.
