# Supplementary material for: The History of African Gene Flow into Southern Europeans, Levantines, and Jews
Source: PLoS Genet. 2011 Apr 21;7(4):e1001373. doi: 10.1371/journal.pgen.1001373 (PMC3080861; doi:10.1371/journal.pgen.1001373)
Supplement: Text S5 — Searching for the source of African ancestry in West Eurasians. (0.05 MB DOC) [file pgen.1001373.s034.doc]

**Text S5. Searching for the source of African ancestry in West Eurasians**

In order to identify the source of the African ancestry in Levantine, Southern Europeans and Jewish groups, we performed Principal Component Analysis (PCA) and *4 Population Test* [1]*.* We first started by establishing axes of variation in Africa by performing - (a) PCA with 15 sub-Saharan African populations and (b) PCA with sub-Saharan Africans, South Africans (HGDP-CEPH- San) and European Americans (HapMap3- CEU). The goal of these analysis was to investigate if we can reliably distinguish ancestry from various parts of Africa and to ensure that none of the African populations have any West Eurasian ancestry. If we include samples that have West Eurasian ancestry, the PCA will be biased toward the population more closely matching the West Eurasian group related to the mixture, which would confound our results. For these analysis, we used the dataset of 10 West and South African populations from Bryc et al. [2], three sub-Saharan African populations from HGDP-CEPH [3] and five populations from HapMap3 [4].

The PCA in Figure S12 shows that most of our samples fall along two main axes of variation, which we call Chadic (e.g. Bulala, Mada and Kaba) vs Non- Chadic and East Africans (e.g. Kenyan Luhya- LWK) vs West Africans (e.g. Nigerian Yoruba - YRI). This pattern is similar to one previously observed by Bryc et al. [2]. In addition, we identified that the East African Maasai have some West Eurasian ancestry as samples vary in their proximity to CEU in the PCA, and hence we did not include them in subsequent PCA explorations.

To make a qualitative inference about the source of the African ancestry in West Eurasians—relative to the Chadic and East-West axes of variation in Africa defined in the PCA analyses—we performed PCA Projection. Specifically, we carried out PCA Projection analysis with three sub-Saharan African populations that are at the extremes of the PCA in Figure S12: Bulala, Yoruba, Luhya that we take to represent Chadic, Niger-Kordofanian, and Nilo-Saharan related ancestry respectively. We also used Asians (HapMap3- CHB) to represent non-African variation. The value of using the CHB rather than a West Eurasian population here is that the CHB are likely to be symmetrically related to all West Eurasians. Hence, including them in the analysis will not bias the results toward matching one West Eurasian group more than another.

We performed projection PCA with all three possible pairs of African populations along with CHB, and then plotted the mean values of all the samples from each West Eurasian population onto the first and second PCs. As a reference, we also project African Americans (HapMap3 ASW) and North Africans (HGDP-CEPH- Mozabite) that have inherited a mixture of both sub-Saharan African and West Eurasian ancestry, as well as Northern Europeans (CEU).

Figure S13 shows that West Eurasians without any evidence of sub-Saharan African ancestry (like CEU) all fall on a single point on the plot, as expected. However, the West Eurasians with sub-Saharan African ancestry fall along a gradient pointing toward some sub-Saharan African populations more than others. The Figure S13A and Figure S13B show that the African ancestry in West Eurasians is likely not related to Chadic Bulala population as in both cases the West Eurasians are pointing away from Bulala. However, when we perform the analysis using Luhya, Yoruba and CHB to construct the PCs, we observe that the West Eurasians are pointing to a population that is intermediate between Kenyans and Yorubans, but somewhat more closely related to the East Africans.

In order to formally test if Levantine, Southern Europeans and Jews are more closely related to Luhya (compared to Yoruba), we perform the *4 Population Test* with the tree ((LWK, YRI),(*X*, CEU) that is consistent with the data, where *X* is a West Eurasian populations (Table S14). We are not able to reject this tree when *X* is any of the Southern European or Jewish groups, and hence we cannot formally reject the hypothesis of at least some West African ancestry in these groups. However, we are able to show that the African ancestry in a couple of Levantine populations is more closely related to East Africans than West Africans. It is historically plausible that gene flow occurred from both West Africa (there were slave caravans across the western Sahara in Roman times [5]) and from East Africa, via the Egypt and Middle East [6].

It is important to note that the methods used in our study for inferring ancestry proportion as well as the date of admixture are carefully designed so that they are not sensitive to which African population is used, as long as the phylogeny is correct. Thus, our inability to pinpoint the African source population for the sub-Saharan African ancestry in West Eurasians is not expected to bias these inferences. To confirm this expectation, we performed our analysis with LWK (instead of YRI) to represent the sub-Saharan African source population and show that our results remain qualitatively similar. Results for *f_4_ Ancestry Estimation* and *ROLLOFF* using East Africans and CEU as ancestral populations are shown in Table S15.

**References**

1. Reich D, Thangaraj K, Patterson N, Price A, Singh L (2009) Reconstructing Indian population history. Nature 461: 489-494.

2. Bryc K, Auton A, Nelson M, Oksenberg J, Hauser S, et al. Genome-wide patterns of population structure and admixture in West Africans and African Americans. Proceedings of the National Academy of Sciences 107: 786.

3. Li J, Absher D, Tang H, Southwick A, Casto A, et al. (2008) Worldwide human relationships inferred from genome-wide patterns of variation. Science 319: 1100.

4. Altshuler D, Brooks L, Chakravarti A, Collins F, Daly M, et al. (2005) A haplotype map of the human genome. Nature 437: 1299-1320.

5. Gibbon E (1890) The decline and fall of the Roman Empire: WW Gibbings.

6. Richards M, Rengo C, Cruciani F, Gratrix F, Wilson J, et al. (2003) Extensive female-mediated gene flow from sub-Saharan Africa into near eastern Arab populations. The American Journal of Human Genetics 72: 1058-1064.
